# Lymphangiogenesis requires Ang2/Tie/PI3K signaling for VEGFR3 cell-surface expression

**DOI:** 10.1172/JCI155478

**Published:** 2022-08-01

**Authors:** Emilia A. Korhonen, Aino Murtomäki, Sawan Kumar Jha, Andrey Anisimov, Anne Pink, Yan Zhang, Simon Stritt, Inam Liaqat, Lukas Stanczuk, Laura Alderfer, Zhiliang Sun, Emmi Kapiainen, Abhishek Singh, Ibrahim Sultan, Anni Lantta, Veli-Matti Leppänen, Lauri Eklund, Yulong He, Hellmut G. Augustin, Kari Vaahtomeri, Pipsa Saharinen, Taija Mäkinen, Kari Alitalo

**Affiliations:** 1 Wihuri Research Institute, Biomedicum Helsinki, Helsinki, Finland.; 2 Translational Cancer Medicine Program, University of Helsinki, Helsinki, Finland.; 3 Department of Immunology, Genetics and Pathology, Uppsala University, Uppsala, Sweden.; 4 Bioengineering Graduate Program, University of Notre Dame, South Bend, Indiana, USA.; 5 Cyrus Tang Hematology Center, Collaborative Innovation Center of Hematology, State Key Laboratory of Radiation Medicine and Protection, Cam-Su Genomic Resources Center, Soochow University, Suzhou, China.; 6 Oulu Centre for Cell-Matrix Research, Faculty of Biochemistry and Molecular Medicine, Biocenter Oulu, University of Oulu, Oulu, Finland.; 7 Division of Vascular Oncology and Metastasis, German Cancer Research Center (DKFZ-ZMBH Alliance), Heidelberg, Germany.; 8 European Center for Angioscience (ECAS), Medical Faculty Mannheim, Heidelberg University, Mannheim, Germany.; 9 iCAN Digital Precision Cancer Medicine Flagship, Faculty of Medicine, University of Helsinki, Helsinki, Finland.

**Keywords:** Vascular Biology, Cardiovascular disease, Endothelial cells, Growth factors

## Abstract

Vascular endothelial growth factor C (VEGF-C) induces lymphangiogenesis via VEGF receptor 3 (VEGFR3), which is encoded by the most frequently mutated gene in human primary lymphedema. Angiopoietins (Angs) and their Tie receptors regulate lymphatic vessel development, and mutations of the *ANGPT2* gene were recently found in human primary lymphedema. However, the mechanistic basis of Ang2 activity in lymphangiogenesis is not fully understood. Here, we used gene deletion, blocking Abs, transgene induction, and gene transfer to study how Ang2, its Tie2 receptor, and Tie1 regulate lymphatic vessels. We discovered that VEGF-C–induced Ang2 secretion from lymphatic endothelial cells (LECs) was involved in full Akt activation downstream of phosphoinositide 3 kinase (PI3K). Neonatal deletion of genes encoding the Tie receptors or Ang2 in LECs, or administration of an Ang2-blocking Ab decreased VEGFR3 presentation on LECs and inhibited lymphangiogenesis. A similar effect was observed in LECs upon deletion of the PI3K catalytic p110α subunit or with small-molecule inhibition of a constitutively active PI3K located downstream of Ang2. Deletion of Tie receptors or blockade of Ang2 decreased VEGF-C–induced lymphangiogenesis also in adult mice. Our results reveal an important crosstalk between the VEGF-C and Ang signaling pathways and suggest new avenues for therapeutic manipulation of lymphangiogenesis by targeting Ang2/Tie/PI3K signaling.

## Introduction

Research in recent years has greatly increased knowledge about lymphatic vessels and vessels with hybrid lymphatic and blood vascular markers, which are now considered to play active roles in several physiological and pathological conditions. The primary function of the lymphatic vascular system is to control tissue fluid homeostasis, trafficking of immune cells, and absorption of dietary fat (1–3). Lymphangiogenesis and abnormal lymphatic vessel function have been associated with conditions such as obesity, atherosclerosis, and neurological disorders (2, 4). Importantly, inhibition of lymphangiogenesis reduces lymphatic metastasis in several mouse models, and lymphangiogenic growth factors can increase the efficacy of immune checkpoint therapy (4–6). Additionally, lymphangiogenic growth factors are currently being used in clinical trials for human lymphedema (4, 7).

In the hierarchical lymphatic vascular network, the blind-ended lymphatic capillaries have discontinuous, button-like cell-cell junctions and a thin and loose basement membrane, permitting efficient absorption of fluid from the interstitial space in tissues. This fluid, lymph, is transported back to the blood circulation via specialized collecting lymphatic vessels, which have a basement membrane and smooth muscle cell (SMC) coverage, endothelial cell zipper junctions, as well as valves to prevent lymph leakage and backflow (2, 8, 9). Vascular endothelial growth factor C (VEGF-C) and its receptor VEGF receptor 3 (VEGFR3) and coreceptor neuropilin 2 (Nrp2) in lymphatic endothelial cells (LECs) are the major drivers of lymphangiogenesis in embryos, and their deficiency leads to failure of lymphatic vessel development (10, 11). The embryonic lymphatic vascular plexus is remodeled into a functional lymphatic vessel network during late embryonic and early postnatal development, when lymphatic vessel growth still depends on VEGF-C/VEGFR3 signaling (3, 12).

Also, deletion of both angiopoietin 1 (Ang1, also known as Angpt1) and Ang2 (also known as Angpt2), their receptor Tie2, or the homologous Tie1, leads to abnormal embryonic lymphatic vessel development and function (13–16). Furthermore, blockade of Ang2 via neutralizing Abs or gene deletion results in lymphatic defects in embryos or in early postnatal pups (17–19).

In blood vessels, Ang1 is an obligatory Tie2 agonist, whereas the agonist activity of Ang2 is context dependent (13). Tie1 forms complexes with Tie2 and regulates its activity in angiogenesis and vascular remodeling (13, 20, 21). Ang binding to the Tie2-Tie1 complex promotes downstream signaling via the phosphoinositide 3 kinase/Akt (PI3K/Akt) pathway (22, 23), resulting in Akt-dependent phosphorylation and nuclear exclusion of the forkhead box O1 (FoxO1) transcription factor, leading to downregulation of its target genes, such as Ang2 (24). In inflammatory conditions, Ang2 attenuates Ang1/Tie2 signaling, leading to increased FoxO1 activity that stimulates endothelial Ang2 expression (20, 25, 26). In contrast, autocrine Ang2 was shown to act as a Tie2 agonist in stressed endothelial cells and in Ang2-overexpressing mice (20, 27).

Ang2 acts as a Tie2 agonist in lymphatic vessels, as they lack vascular endothelial protein tyrosine phosphatase (VE-PTP/PTPRβ), which dephosphorylates Tie2 (16, 28). Interestingly, mutations in Ang2 and genetic variants of Tie1 were recently associated with primary lymphedema in humans (29, 30). However, the mechanisms of Ang/Tie signaling in the lymphatic vasculature remain incompletely understood (13, 31). A more profound understanding of how the Ang/Tie pathway regulates lymphangiogenesis is crucial, as this pathway is implicated in inflammation, lymphedema, and lymphatic metastasis (13, 29, 30, 32, 33).

Here, we investigated the crosstalk of Ang2/Tie and VEGF-C/VEGFR3 signaling pathways in lymphangiogenesis using multiple transgenic and gene-targeted mouse models to manipulate Tie1, Tie2, Ang2, PI3K, and VEGF-C in postnatal and adult mice. We discovered that Ang2 and the Tie receptors controlled lymphangiogenesis by regulating cell-surface presentation and turnover of VEGFR3 via modulation of PI3K activity. Our data therefore uncover a new, translationally exploitable mechanistic link between the 2 major lymphangiogenic growth factor pathways.

## Results

### Impaired postnatal lymphatic capillary network development upon Tie1 deletion or blocking of Ang2.

**Figure 1:**
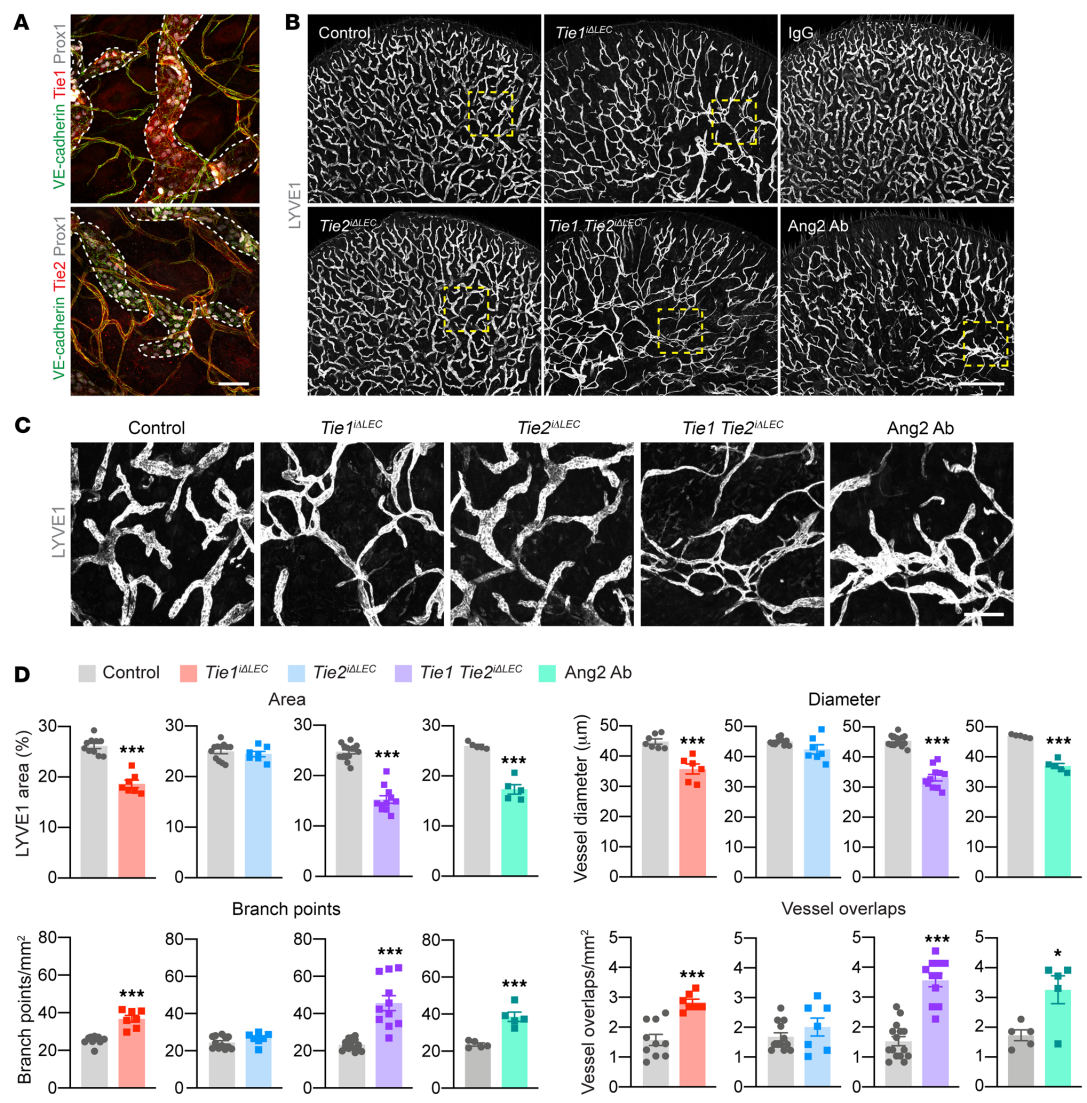
Postnatal Tie1, Ang2, or Tie1/-2 deficiency results in an abnormal cutaneous lymphatic capillary network. (**A**) Representative images of VE-cadherin, Prox1, Tie1, and Tie2 immunostaining in ear skin from P21 pups. Dashed lines indicate lymphatic capillaries. Scale bar: 50 μm. (**B**) LYVE1 staining of ventral ear skin from control, Tie1-deleted (control: *n =* 10; Tie1-deleted: *n =* 7), Tie2-deleted (control: *n =* 12; Tie2-deleted *n =* 7), Tie1/-2–deleted (control: *n =* 15; Tie1/-2–deleted: *n =* 11), lgG-treated (*n =* 5), and Ang2 Ab–treated (*n =* 5) pups on P21. Scale bar: 1 mm. (**C**) Magnification of the area outlined by the dashed boxes in **B**. Scale bar: 200 μm. (**D**) Quantification of the LYVE1-positive vessel area, vessel diameter, branch points, and vessel overlaps. Data represent the mean ± SEM. *P < 0.05 and ***P < 0.001, by 2-tailed Student’s *t* test.

To investigate the function of Tie1, Tie2, and Ang2 in postnatal lymphangiogenesis, we first analyzed the expression of Tie1 and Tie2 in cutaneous vasculature. Immunofluorescence staining of the ear skin of pups on P21 indicated that Tie1 was expressed by endothelial cells in both blood vessels and lymphatic capillaries (Figure 1A). Tie2 expression was strong in blood vessels, but very weak in lymphatic capillaries, in which Tie1 was abundant (Figure 1A).

To delete the Tie receptors specifically from the LECs of neonatal mice, we crossed mice carrying *Tie1^fl^* or *Tie2^fl^* alleles with *Prox1CreER^T2^* deletor mice and administered 4-hydroxytamoxifen (4-OHT) by daily intragastric injections at P1–P5 (Supplemental Figure 1A; supplemental material available online with this article; https://doi.org/10.1172/JCI155478DS1). To compare the functions of Tie1, Tie2, and Ang2 in postnatal lymphatic vessel development, we analyzed mice neonatally deleted of Tie1 (*Tie1^iΔLEC^*), Tie2 (*Tie2^iΔLEC^*), or both receptors (*Tie1* *Tie2^iΔLEC^*), or injected with Ang2-blocking Abs every 3 days, starting on P1 (Supplemental Figure 1B). We confirmed Tie1 and Tie2 deletions by staining the ear skin and by quantitative reverse transcription PCR (RT-qPCR) analysis of receptor mRNA in LECs and, for comparison, in blood vascular endothelial cells (BECs) isolated from Tie1- and Tie2-deleted ear skin (Supplemental Figure 1, C–H).

Immunofluorescence staining of the skin using Abs against the lymphatic vessel endothelial hyaluronan receptor 1 (LYVE1) showed that Tie1 deletion, Tie1 and Tie2 (Tie1/-2) double-deletion, and Ang2 blocking resulted in abnormal lymphatic capillaries, consisting of a disorganized lymphatic capillary network with an increased distance between lymphatic vessels and loop-like and overlapping vessel structures (Figure 1, B and C). This phenotype was similar to the phenotype described after postnatal deletion of the intracellular domain of Tie1 or constitutive Ang2 deletion (19, 34). In contrast, the lymphatic capillary network in the Tie2-deleted mice did not differ from that in control littermate mice (Figure 1, B and C) (34), in line with the low Tie2 expression in lymphatic capillaries (Figure 1A and Supplemental Figure 1D). Quantification of vascular parameters revealed reduced lymphatic vessel areas, smaller vessel diameters, increased numbers of branch points, and overlapping vessels in the Tie1-deleted, Tie1/-2 double-deleted, and Ang2 Ab–treated pups, when compared with their littermate control pups (Figure 1D).

### Defective postnatal collecting lymphatic vessel development upon combined deletion of Tie1 and Tie2.

**Figure 2:**
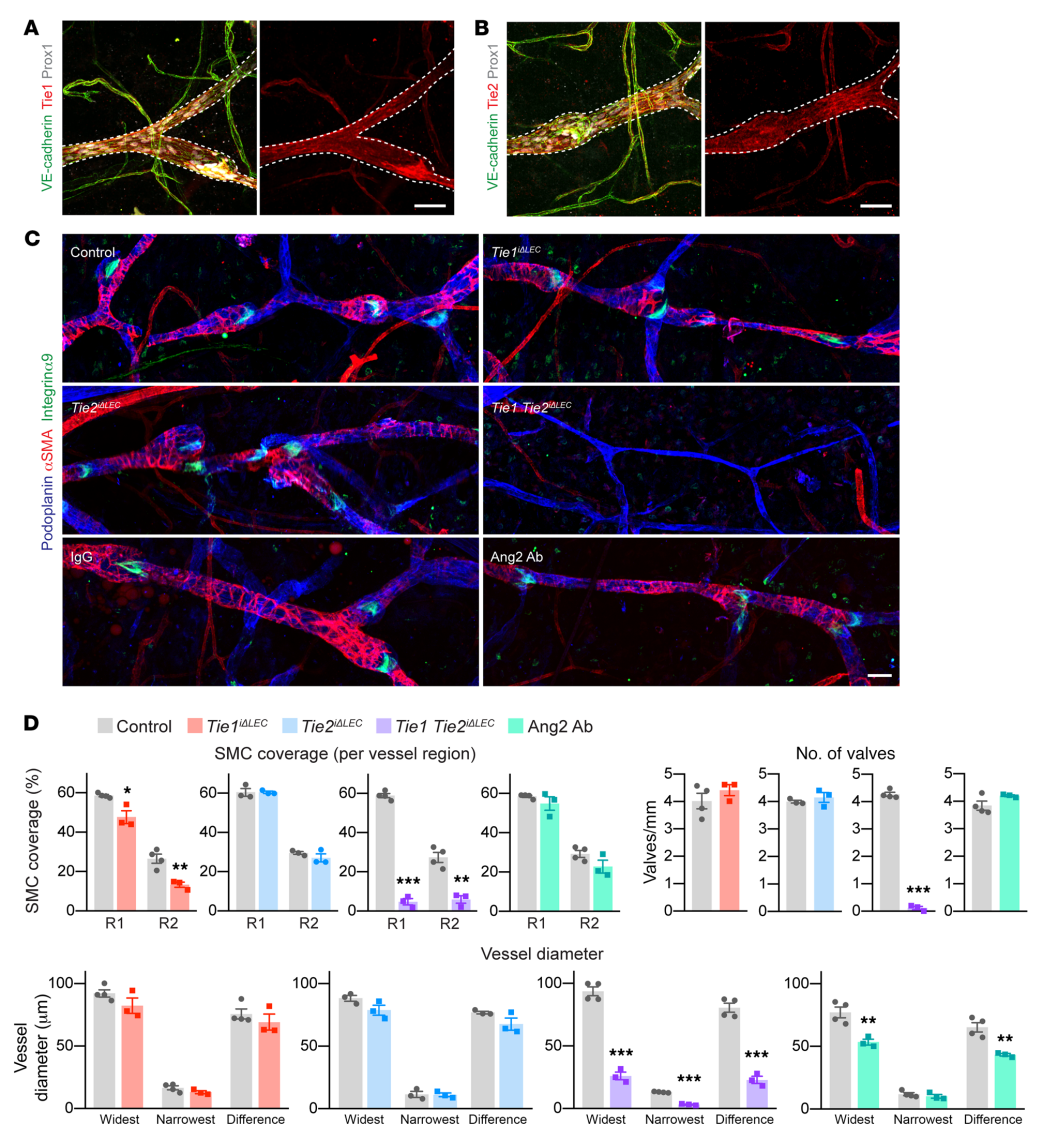
Double-deletion of Tie1 and Tie2 leads to reduced SMC coverage and a lack of valves in collecting lymphatic vessels. (**A** and **B**) Representative images of P21 ear skin immunostained for VE-cadherin, Prox1, and Tie1 or Tie2. Dashed lines indicate the collecting lymphatic vessels. Scale bars: 50 μm. (**C**) Dorsal ear skin of Tie1-deleted (control: *n =* 4; Tie1-deleted: *n =* 3), Tie2-deleted (control: *n =* 3; Tie2-deleted *n =* 3), Tie1/-2–deleted (control: *n =* 4; Tie1/-2–deleted *n =* 3), and Ang2 Ab–treated (IgG: *n =* 4; Ang2 ab: *n =* 3) P21 pups, immunostained for podoplanin, αSMA, and integrin α9. Scale bar: 50 μm. (**D**) Quantification of SMC coverage per collecting lymphatic vessel region, the number of valves, the widest and narrowest vessel diameters measured from entire collecting lymphatic vessels, and the differences between the two. R1 and R2 indicate the proximal and distal lymphatic collecting vessel regions, respectively. Data represent the mean ± SEM. *P < 0.05, **P < 0.01, and ***P < 0.001, by 2-tailed Student’s *t* test.

In contrast to lymphatic capillaries, both Tie2 and Tie1 were expressed in the collecting lymphatic vessels (Figure 2, A and B, and Supplemental Figure 1, E and F). To analyze the effects of Tie1, Tie2, and Ang2 on postnatal collecting lymphatic vessel development, lymphatic valves, and SMC coverage of the vessels, we stained cutaneous vessels in the Tie-deleted and Ang2-inhibited pups with Abs against podoplanin, integrin α9, and α-smooth muscle actin (αSMA). The Tie1-deleted pups had reduced SMC coverage, and the Ang2-inhibited mice had thinner collecting lymphatic vessels than did their respective control pups, whereas no significant differences in valve numbers were observed (Figure 2, C and D). Tie1-deleted pups had somewhat smaller collecting lymphatic vessel diameters in the proximal region and Tie2-deleted pups in the distal regions of the ears (Figure 2, C and D, and Supplemental Figure 2A). The Tie1/-2 double-deleted mice lacked valves almost completely in the collecting lymphatic vessels, which were thinner than in those of the control mice, and some were even discontinuous or had an abnormal morphology and only occasional SMCs (Figure 2, C and D, and Supplemental Figure 2B). These changes were accompanied by apoptosis of LECs, as evidenced by staining for cleaved caspase 3 (Supplemental Figure 2C). These findings were consistent with the high expression levels of both Tie1 and Tie2 in the collecting lymphatic vessels.

### Ang2-induced collecting lymphatic vessel enlargement requires Tie1 and Tie2.

**Figure 3:**
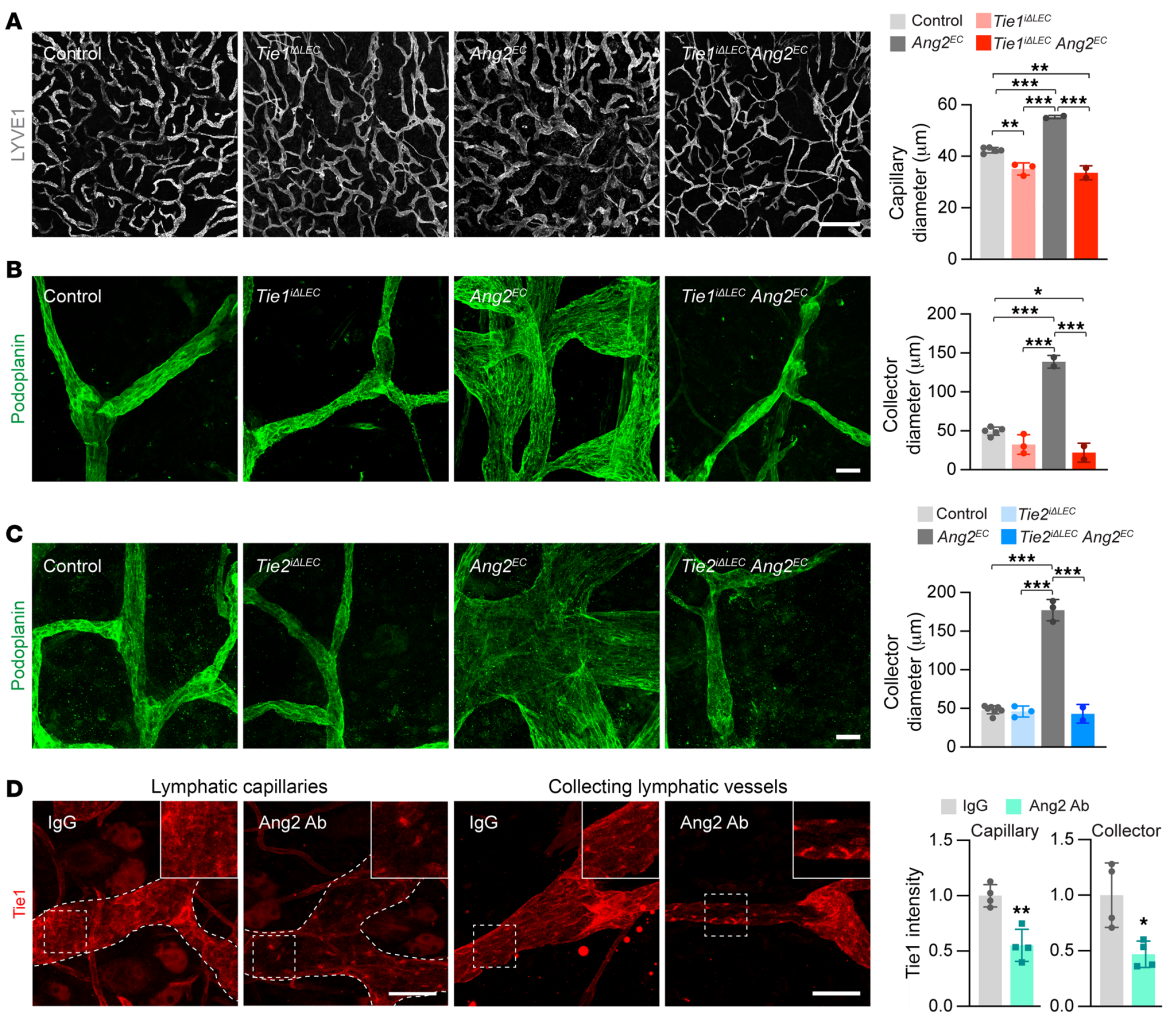
Requirement of Tie1 and Tie2 for Ang2-induced lymphatic vessel enlargement and Ang2 for Tie1 expression on the surface of LECs. (**A**) Images of LYVE1 staining of ventral ear skin in control (*n =* 5), Tie1-deleted (*Tie1^iΔLEC^*, *n =* 3), Ang2-overexpressing (*Ang2^EC^*, *n =* 2), and *Tie1^iΔLEC^* *Ang2^EC^* (*n =* 2) p21 pups. Scale bar: 500 μm. Graph shows quantification of the average lymphatic capillary diameter. (**B**) Images of podoplanin staining of collecting lymphatic vessels in dorsal ear skin from the pups indicated in **A** on P21. Scale bar: 50 μm. Graph shows quantification of the widest collecting lymphatic vessel diameter. (**C**) Images of podoplanin staining of collecting lymphatic vessels in dorsal ear skin in control (*n =* 7), Tie2-deleted (*Tie2^iΔLEC^*, *n =* 3), Ang2-overexpressing (*Ang2^EC^*, *n =* 3), and *Tie2^iΔLEC^* *Ang2^EC^* (*n =* 2) pups on P21. Scale bar: 50 μm. Graph shows quantification of the widest collecting lymphatic vessel diameter. (**D**) Images of Tie1 staining and quantification in lymphatic capillaries and collecting vessels in ear skin from IgG- (*n =* 4) and Ang2 Ab–treated (*n =* 4) pups on P21, normalized to control. Scale bars: 50 μm. Magnification: 1.87. Data represent the mean ± SEM. **P < 0.01 and ***P < 0.001, by 1-way ANOVA with Bonferroni’s post hoc test for multiple comparisons (**A**–**C**) and 2-tailed Student’s *t* test (**D**).

To further study the function of Ang2 in postnatal lymphatic growth, we used a tetracycline-regulated genetic mouse model of endothelial Ang2 overexpression (*VE-cadherin-tTA* *Tet-OS-Ang2*, hereafter referred to as *Ang2^EC^*) (33), into which we introduced the *Prox1CreER^T2^* deletor and conditional Tie1 or Tie2 alleles. We induced Ang2 expression on E12.5, deleted Tie1 or Tie2 on P1, and analyzed the lymphatic vessels on P21 by staining of the ear skin for LYVE1 or podoplanin (Figure 3, A–C). We found that Ang2 induction enlarged the lymphatic capillaries moderately and the collecting lymphatic vessels more prominently (Figure 3, A–C). However, this did not occur in the Tie1-deleted pups, indicating that Tie1 was necessary for Ang2 signaling in LECs (Figure 3, A and B). Likewise, Tie2 deletion on P1 prevented the Ang2-induced collecting lymphatic vessel enlargement (Figure 3C). Overall, these results indicated that both Tie1 and Tie2 were required for Ang2-induced collecting lymphatic vessel enlargement.

### Blocking of Ang2 leads to decreased cell-surface expression of Tie1 in lymphatic vessels.

Surprisingly, Tie1 was not stained on the surface of LECs, but only perinuclearly in both lymphatic capillaries and collecting vessels in P21 pups that had been injected with Ang2-blocking Abs every 3 days starting on P1 (Figure 3D and Supplemental Figure 3A). We found that Tie1 staining was perinuclear also in the disorganized and thin cutaneous lymphatic vessels of *Angpt2* gene–deleted mice (*Ang2^-/-^*) (Supplemental Figure 3, B–D). However, blocking of Ang2 did not affect the cell-surface localization of Tie2 in the collecting lymphatic vessels, although the Ang2-knockout pups showed somewhat reduced Tie2 levels (Supplemental Figure 3, E and F). These results indicated that Ang2 was essential for cell-surface localization of Tie1 in lymphatic vessels.

### Decreased cell-surface expression of VEGFR3 in Tie1- or Ang2-deficient lymphatic vessels.

**Figure 4:**
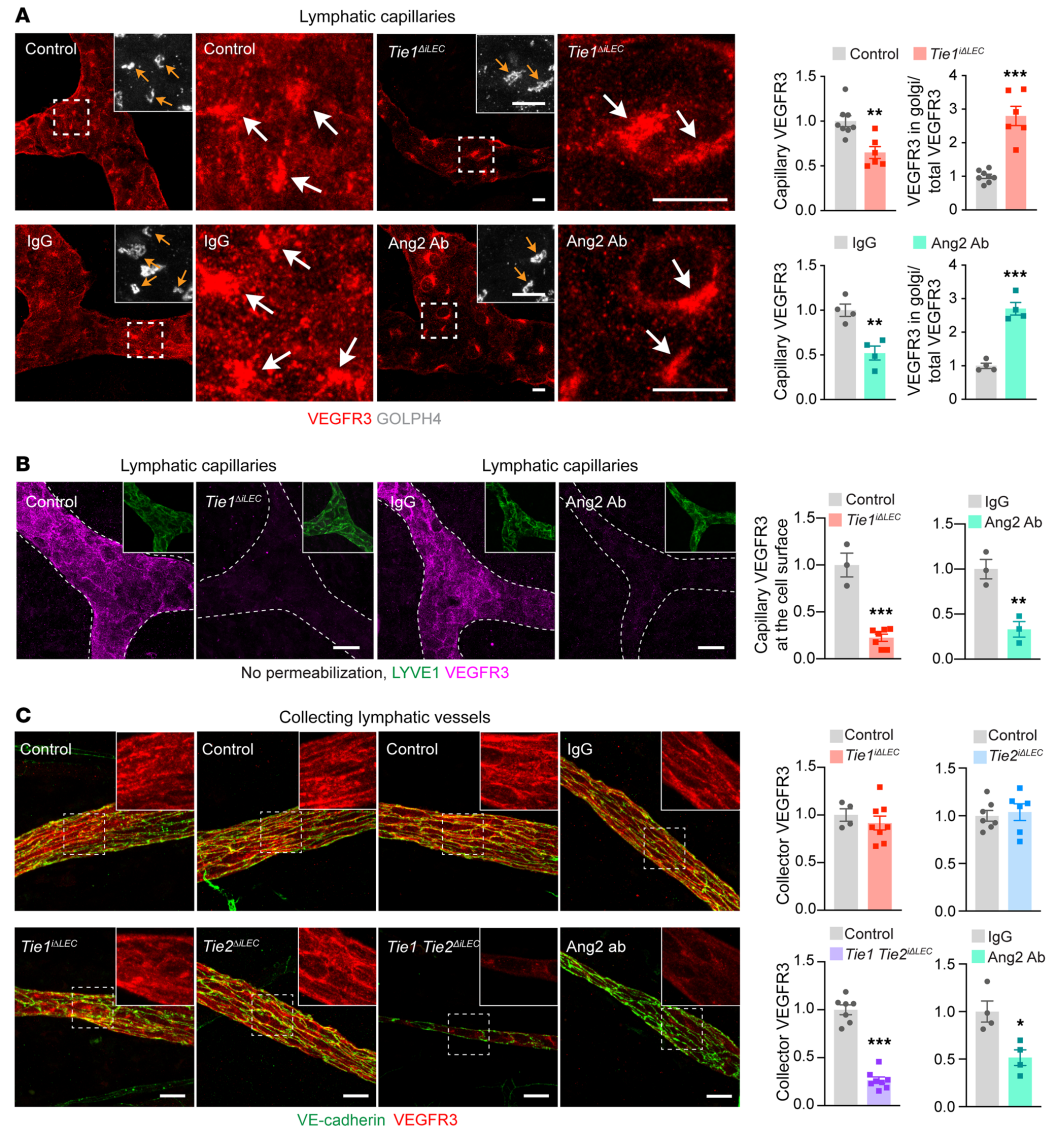
Lack of Ang2 or Tie receptors leads to loss of VEGFR3 from the LEC surface. (**A**) Images of lymphatic capillaries in ear skin from control (*n =* 8), Tie1-deleted (*n =* 6), IgG-treated (*n =* 4), and Ang2 Ab–treated (*n =* 4) P21 pups, immunostained for VEGFR3 and GOLPH4. Arrows point to VEGFR3 in the Golgi complex. Quantifications show a decrease in VEGFR3 immunofluorescence in capillary LECs and an increase in Golgi complex VEGFR3 immunofluorescence compared with total VEGFR3, normalized to control. Scale bars: 10 μm. (**B**) Cell-surface immunostaining for VEGFR3 in lymphatic capillaries of the ear skin from Tie1-deleted (control: *n =* 3; Tie1-deleted: *n =* 7) and Ang2-inhibited (*n =* 3 per group) P21 pups. Graphs show quantification of VEGFR3 immunofluorescence at the cell surface, normalized to control. Scale bars: 20 μm. Magnification: 0.40. (**C**) Immunostaining of VEGFR3 and VE-cadherin in collecting lymphatic vessels in ear skin from Tie1-deleted (control: *n =* 4; Tie1-deleted: *n =* 8), Tie2-deleted (control: *n =* 7; Tie2-deleted *n =* 6), Tie1/-2–deleted (control: *n =* 7; Tie1/-2–deleted *n =* 8), and Ang2 Ab–treated (*n =* 4 per group) P21 pups. Quantification of VEGFR3 immunofluorescence in collecting lymphatic vessels, normalized to control. Scale bars: 20 μm. Magnification: 1.86. Data represent the mean ± SEM. *P < 0.05, **P < 0.01, and ***P < 0.001, by 2-tailed Student’s *t* test.

To dissect how Tie receptor deficiency and Ang2 inhibition lead to atrophy of lymphatic vessels, we analyzed VEGFR3, which is the major lymphangiogenic growth factor receptor in LECs (35). We first stained VEGFR3 in cutaneous lymphatic capillaries in the Tie-deleted or Ang2 Ab–treated pups on P21. Surprisingly, Tie1 deletion, Ang2 inhibition, or combined Tie1/-2 deletion resulted in loss of VEGFR3 staining from the surface of LECs, leaving just a perinuclear staining pattern that colocalized with the Golgi complex marker GOLPH4 (Figure 4, A and B, and Supplemental Figure 4, A–C). Tie2 deletion resulted in a small decrease of total VEGFR3 fluorescence, but it did not affect VEGFR3 distribution or VEGFR3 staining on the surface of LECs (Supplemental Figure 4, D and E). We next stained VEGFR3 in the lymphatic capillaries of Ang2-knockout pups at P21 and found that VEGFR3 was lost from the LEC surface in a manner similar to what we observed in the Ang2 Ab–treated pups (Supplemental Figure 4, F and G). We confirmed this phenotype by using both *Prox1-CreER^T2^*– and *Cdh5-CreER^T2^*–mediated deletion of Ang2 (*Ang2^iΔLEC^* and *Ang2^iΔEC^*, respectively) in P1 pups (Supplemental Figure 5, A–D). These data indicated that the LEC-derived Ang2 acted as an autocrine factor to regulate lymphatic vessel development in postnatal mice.

To investigate the kinetics of VEGFR3 regulation by Ang2/Tie signaling in lymphatic capillaries, we administered Ang2 Abs to P12 pups and analyzed VEGFR3 expression levels at different time points after the blocking of Ang2. Already 2 days after Ang2 Ab administration, on P14, most of the VEGFR3 staining was found in the perinuclear location, and similar results were obtained on P15, P16, and P21 (Supplemental Figure 6). These results indicate that loss of VEGFR3 surface expression occurred rapidly upon Ang2 blockade.

Unlike in the lymphatic capillaries, neither Tie1 nor Tie2 deletion affected VEGFR3 localization significantly in the collecting lymphatic vessels (Figure 4C and Supplemental Figure 7). In contrast, Tie1/-2 double-deletion as well as Ang2 blockage or deletion resulted in reduced VEGFR3 immunofluorescence in the collecting lymphatic vessels (Figure 4C, Supplemental Figure 4F, and Supplemental Figure 7). Overall, these results indicate that Tie1 and Ang2 were required for proper presentation of VEGFR3 on the surface of LECs in lymphatic capillaries and that both Tie receptors and Ang2 were required for the same function in collecting lymphatic vessels.

### Effect of Ang2 and VEGF-C on cell-surface expression and internalization of VEGFR3 and Tie1.

**Figure 5:**
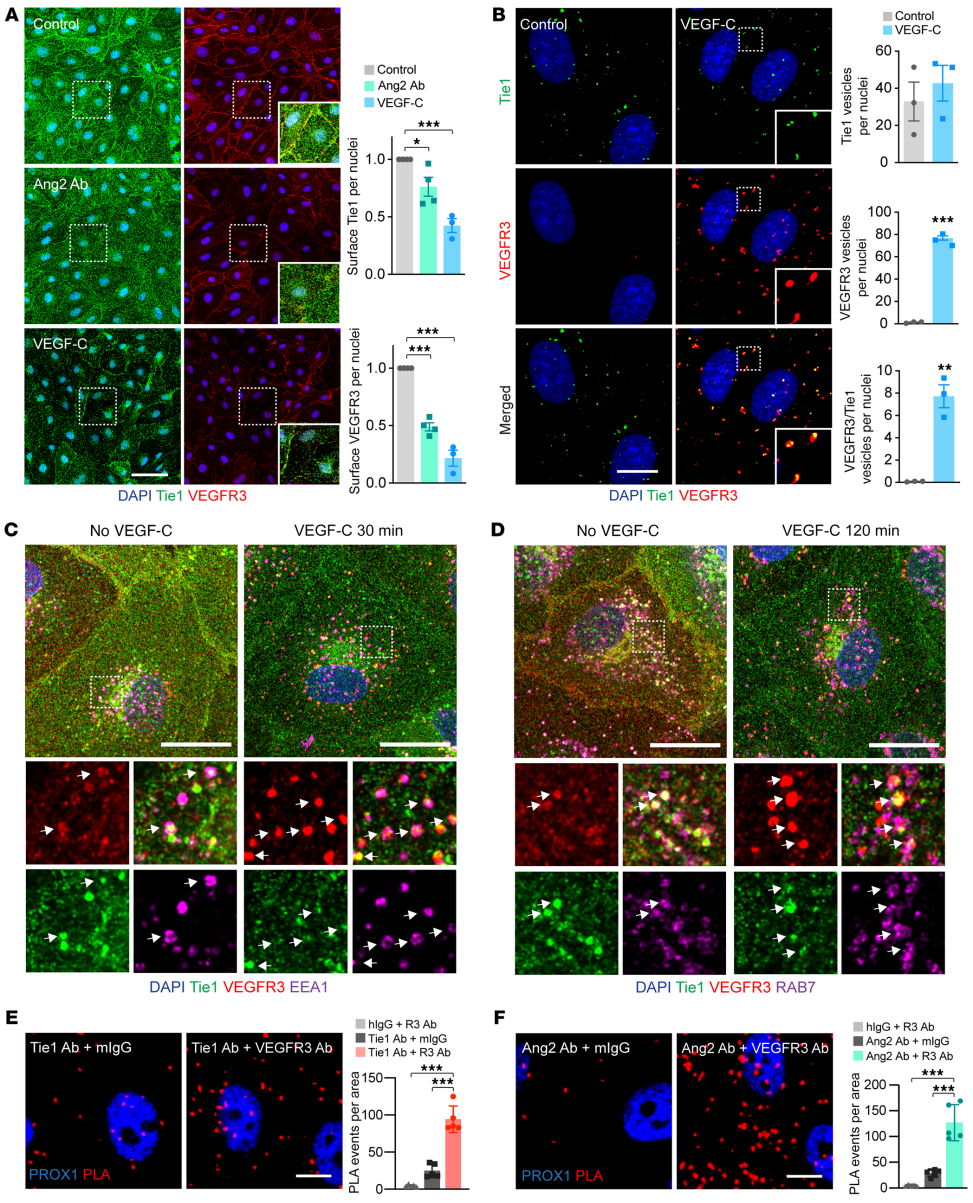
Effect of Ang2 Ab and VEGF-C on the localization of VEGFR3 and Tie1 in cultured LECs. (**A**) Cell-surface staining of VEGFR3 and Tie1 in LECs treated with a control IgG (*n =* 4) or Ang2 Ab (*n =* 4) for 30 minutes or stimulated with VEGF-C (*n =* 3) for 20 minutes. Scale bar: 50 μm. Magnification: 1.66. Graphs show quantification of VEGFR3 and Tie1 staining on the cell surface normalized to the IgG control. (**B**) Images showing colocalization of VEGFR3 and Tie1 in intracellular vesicles of LECs stimulated with VEGF-C for 20 minutes. Scale bar: 10 μm. Graph shows quantification of the number of VEGFR3- and Tie1-positive vesicles per nuclei (*n =* 3). Magnification: 2.36. (**C** and **D**) Colocalization of VEGFR3 with Tie1 and EEA1 (sorting endosomes) after 30 minutes of VEGF-C exposure (**C**) or RAB7 (late endosomes) after 120 minutes of VEGF-C exposure (**D**). The overview image shows a maximum projection. The boxed regions are shown as zoomed-in images of 1 optical slice per channel. White arrows point to some of the triple-positive (VEGFR3, Tie1, and EEA1 or RAB7) vesicles. Scale bars: 20 μm. Magnification: 3.24. (**E** and **F**) PLA immunofluorescence and quantification of PLA spots for Tie1 and VEGFR3 (**E**) and Ang2 and VEGFR3 (**F**) in permeabilized LECs (*n =* 5 fields of view; experiments were repeated 3 times with similar results). Scale bars: 10 μm. Data represent the mean ± SEM. *P < 0.05, **P < 0.01, and ***P < 0.001, by 1-way ANOVA with Dunnett’s post hoc test for multiple comparisons (**A**, **E**, and **F**) and 2-tailed Student’s *t* test (**B**).

To further study the localization of VEGFR3 and Tie1 on the cell surface, we used cultured microvascular LECs, which produce Ang2 (36). Immunofluorescence staining of nonpermeabilized LEC monolayers showed that VEGFR3 and Tie1 were enriched at cell-cell junctions in the absence of VEGF-C, and treatment with VEGF-C or anti-Ang2 Ab reduced the cell-surface presentation of both receptors (Figure 5A and Supplemental Figure 8). To study the internalization of VEGFR3 and Tie1, we treated LECs with nonactivating fluorescently labeled monoclonal anti-VEGFR3 and anti-Tie1 Abs on ice and subsequently incubated the cultures at +37°C for 20 minutes in the absence or presence of VEGF-C to allow internalization of the labeled receptors. Then, cell-surface–bound Abs were detached using a low-pH buffer, followed by paraformaldehyde fixation of the cells, enabling detection of only the internalized receptors (Figure 5B). The number of Tie1-positive vesicles was not affected by VEGF-C treatment, whereas VEGF-C increased the number of VEGFR3-positive vesicles and, interestingly, double-positive (VEGFR3 and Tie1) vesicles (Figure 5B). Next, we monitored VEGFR3 and Tie1 internalization in living cells using time-lapse microscopy. In line with the results from fixed cells, in the presence of VEGF-C, we found that the Ab-labeled VEGFR3 and Tie1 receptors were accumulated in vesicles that contained both receptors (Supplemental Video 1).

To characterize the internalization routes of endogenous Tie1 and VEGFR3, we first investigated their colocalization with markers of endosomes in LECs expressing EGFP-Ras-related protein Rab-5C (RAB5C). Thirty minutes after VEGF-C stimulation, VEGFR3 localized to EGFP-RAB5C and early endosome antigen 1–positive (EEA1-positive) sorting endosomes (Figure 5C and Supplemental Figure 9), whereas at 120 minutes, VEGFR3 localized to RAB7-positive late endosomes (Figure 5D and Supplemental Figure 9). At 240 minutes, we found considerably less VEGFR3 in the RAB7-positive late endosomes (Supplemental Figure 9), which is in line with the role of late endosomes in the transport of proteins for degradation (37). Accordingly, VEGF-C treatment resulted in colocalization of VEGFR3 and Tie1 in sorting endosomes (EEA1) at 30 minutes and in RAB7-positive late endosomes at 120 minutes (Figure 5, C and D). As a complementary approach, we labeled endogenous Tie1 and VEGFR3 on the surface of living LECs with nonactivating, fluorescently conjugated monoclonal anti-VEGFR3 and anti-Tie1 Abs and analyzed the localization 60 minutes after VEGF-C stimulation. Also here, both of the receptors were found in RAB7-positive vesicles (Supplemental Figure 10). These results show that VEGF-C drove VEGFR3 and Tie1 to the same internalization route.

To test whether VEGFR3 and Tie1 are located in close proximity to each other, we performed a proximity ligation assay (PLA) (38). The PLA signal was developed by a rolling circle polymerization of a fluorescent reporter DNA, primed with oligonucleotide-linked secondary Abs that bind anti-Tie1 or anti-VEGFR3 bound to LECs. We found a significantly stronger punctate PLA signal in permeabilized LECs treated with Tie1 and VEGFR3 Abs than in LECs treated with corresponding IgGs (Figure 5E). Interestingly, we also observed strong PLA staining with VEGFR3 and Ang2 Abs, but not with VEGFR3 plus control IgG or VEGFR3 plus Notch1 Abs (Figure 5F and Supplemental Figure 11). We thus confirmed the existence of a pool of VEGFR3 and Tie1 receptors that were in close proximity to each other and to Ang2 in cultured LECs, and that VEGFR3 and Tie1 shared the same internalization route.

### Ang2 controls the expression of mature VEGFR3 and PI3K activity.

**Figure 6:**
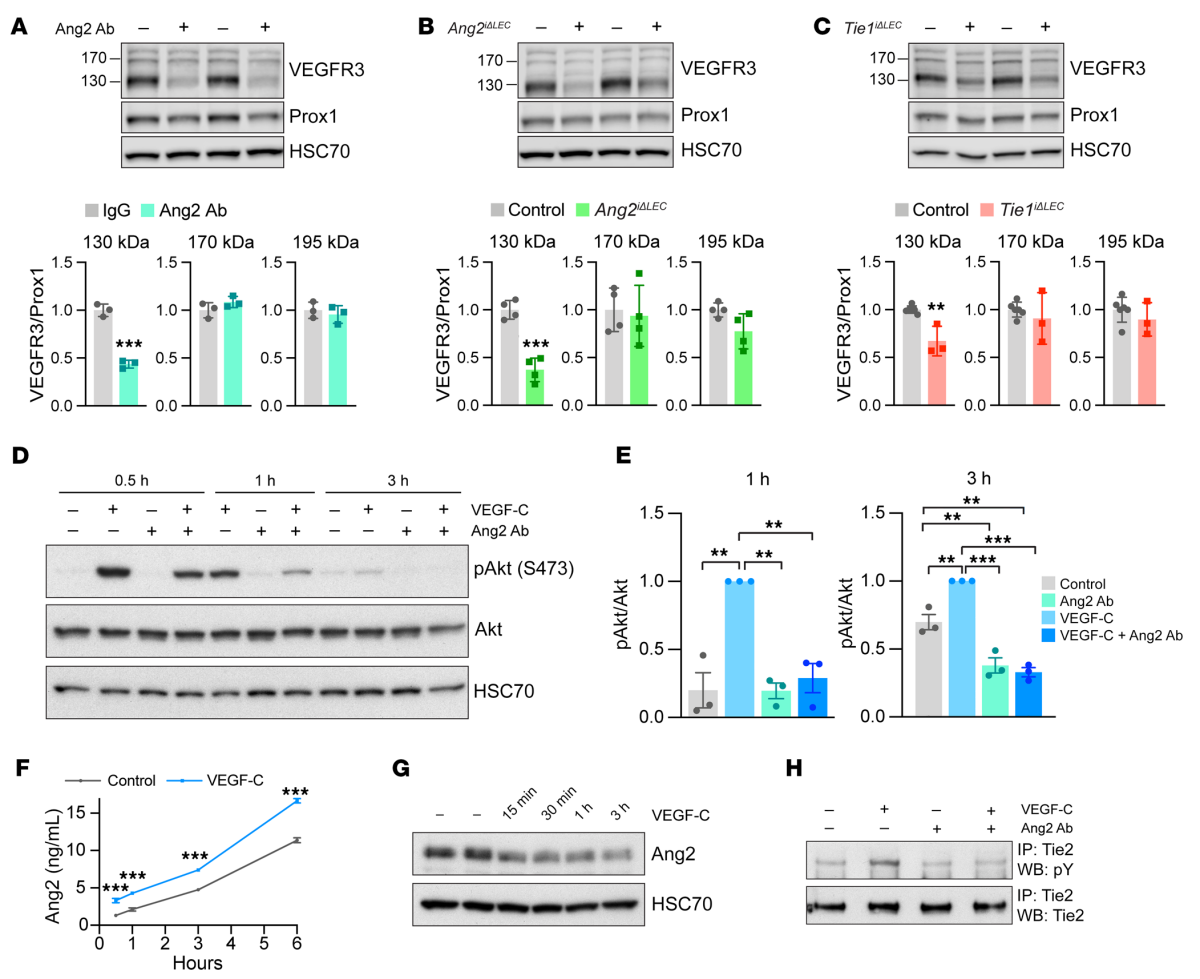
Blocking of Ang2 or deletion of Ang2 or Tie1 decreases VEGFR3 expression and signaling. (**A**–**C**) Western blots showing VEGFR3, Prox1, and HSC70 in ear lysates from control, Ang2-inhibited (*n =* 3 per group) (**A**), Ang2-deleted (*n =* 4 per group) (**B**), and Tie1-deleted (control: *n =* 6; Tie1-deleted: *n =* 3) (**C**) pups. Graphs show quantification of VEGFR3 polypeptides normalized to Prox1 and the control. (**D**) Western blots showing p-Akt, Akt, and HSC70 detection in VEGF-C and Ang2 Ab–treated LECs. (**E**) Quantification of the p-Akt/Akt ratio (*n =* 3 per group), normalized to VEGF-C-treated samples. (**F**) Ang2 concentration in LEC culture medium at the indicated time points after VEGF-C stimulation (*n =* 3 per group). (**G** and **H**) Western blots (WB) showing Ang2 and HSC70 in VEGF-C stimulated LECs (**G**) and Tie2 phosphorylation in VEGF-C–stimulated (45 min) and Ang2 Ab–treated LECs (**H**). The experiments in **G** and **H** were performed twice with similar results. Data represent the mean ± SEM (**A**–**C** and **E**) and ± SD (**F**). **P < 0.01 and ***P < 0.001, by 2-tailed Student’s *t* test (**A**–**C** and **F**) and 1-way ANOVA with Bonferroni’s post hoc test for multiple comparisons (**E**).

Because of the finding that the Ang2 Ab reduced VEGFR3 expression on the surface of LECs both in vivo and in vitro, we next analyzed VEGFR3 by Western blotting of ear skin extracts from pups treated with Ang2 Ab or with tamoxifen to delete Ang2 or Tie1 in LECs (Figure 6, A–C). In Western blots, VEGFR3 polypeptides appears as a nascent, unglycosylated polypeptide of 170 kDa, a fully glycosylated polypeptide of 195 kDa, and “mature” disulfide-linked polypeptides of 130 and 85 kDa on the cell surface, generated from the 195 kDa form by proteolytic cleavage (39). We first confirmed the presence of 195, 130, and 85 kDa forms of VEGFR3 on the surface of LECs by subjecting the cells to trypsin treatment (Supplemental Figure 12, A and B). We then compared the VEGFR3 polypeptides from Ang2 Ab–treated, Ang2-deleted, and Tie1-deleted pups by Western blotting. Interestingly, we found that the 130 kDa proteolytically cleaved mature form of VEGFR3 on the cell surface was decreased in samples from Ang2 Ab–treated, Ang2-deleted, and Tie1-deleted pups, whereas the 170 kDa and 195 kDa forms were not affected (Figure 6, A–C). In contrast, we found no significant differences in VEGFR3 polypeptides between the control and Tie2-deleted pups (Supplemental Figure 12C). These results indicate that Ang2 and Tie1 in LECs regulated the expression of mature cell-surface VEGFR3.

In order to understand the downstream effects of Ang2 deficiency, we analyzed the activation of the PI3K pathway, which is involved in receptor recycling and serves as the key signaling pathway activated downstream of the Tie receptors (22, 23, 40). We treated LECs first with Ang2 Ab, stimulated them with VEGF-C, and analyzed VEGFR3 and phosphorylated Akt (p-Akt) as a downstream indicator of PI3K activity (Figure 6D and Supplemental Figure 12D). As expected, VEGF-C treatment reduced the 195 and 130 kDa cell-surface forms of VEGFR3, which are known to be phosphorylated by VEGF-C stimulation, leading to their internalization and degradation in LECs (Supplemental Figure 12D) (41, 42). Furthermore, VEGF-C induced a robust phosphorylation of Akt, which was reduced by Ang2 Ab treatment (Figure 6, D and E). In contrast, ERK phosphorylation was not significantly affected by Ang2 Ab treatment (Supplemental Figure 12, E and F). When exploring the crosstalk between the VEGF-C and Ang pathways, we furthermore found that VEGF-C stimulation of LECs increased the release of Ang2 and a soluble Tie1 fragment into the culture medium (Figure 6, F and G, and Supplemental Figure 12G) (43). VEGF-C stimulation also increased Tie2 phosphorylation, and this was reduced in anti-Ang2 Ab–treated LECs (Figure 6H). These results indicate that Ang2 was required for expression of the mature cell-surface form of VEGFR3 and VEGF-C–induced PI3K activation downstream of Tie2.

### Pik3ca deletion recapitulates the abnormal postnatal lymphangiogenesis and reduced VEGFR3 levels found in Ang2/Tie-deleted mice.

**Figure 7:**
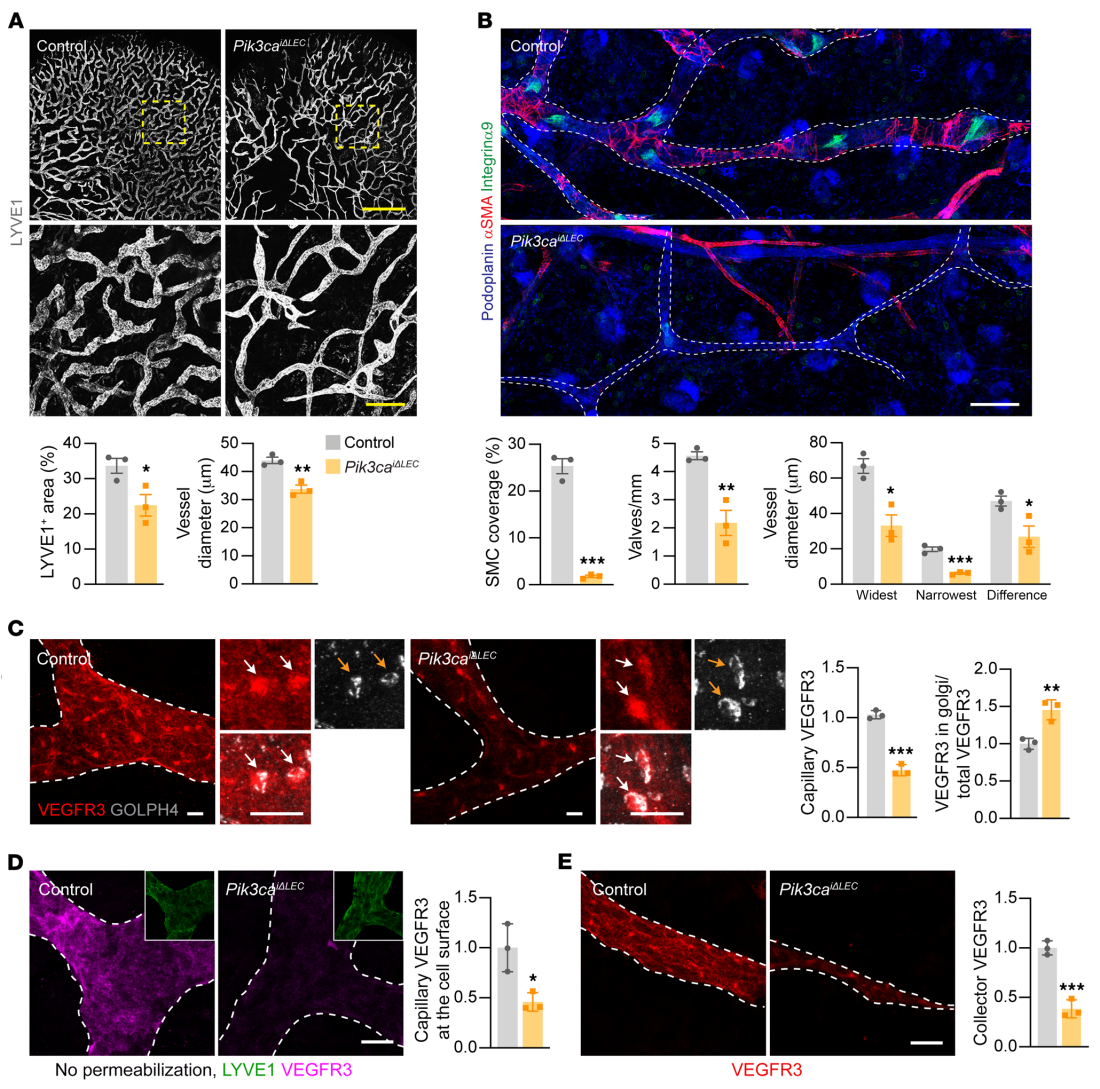
PI3K signaling regulates postnatal lymphatic vessel development and VEGFR3 expression. (**A**) LYVE1 staining of ventral ear skin from control and *Pik3ca*-deleted P21 pup (*n =* 3 per group). Scale bar: 1 mm and 200 μm (higher-magnification insets). Graphs show quantification of the LYVE1-positive vessel area and vessel diameter. (**B**) Dorsal ear skin was immunostained for podoplanin, αSMA, and integrin α9. Scale bar: 100 μm. Graphs show quantification of SMC coverage, the number of valves, and the widest and narrowest vessel diameters and the difference between the 2 in collecting lymphatic vessels. (**C**) Lymphatic capillaries were immunostained for VEGFR3 and GOLPH4. Arrows point to VEGFR3 in the Golgi complex. Scale bars: 10 μm. Graphs show quantification of total VEGFR3 and its fraction in the Golgi complex, normalized to control. (**D**) Cell-surface immunostaining for VEGFR3 in lymphatic capillaries. Graph shows quantification of VEGFR3 immunofluorescence at the cell surface, normalized to control. Scale bar: 20 μm. Magnification: 0.40. (**E**) Immunostaining for VEGFR3 in collecting lymphatic vessels. Scale bar: 20 μm. Graph shows quantification of VEGFR3 immunofluorescence in collecting lymphatic vessels, normalized to control. Data represent the mean ± SEM. *P < 0.05, **P < 0.01, and ***P < 0.001, by 2-tailed Student’s *t* test.

To study the role of PI3K signaling in postnatal lymphangiogenesis, we genetically deleted *Pik3ca*, encoding the catalytic p110α subunit of PI3K, in LECs by crossing mice carrying *Pik3ca^fl^* alleles with mice expressing the *Prox1CreER^T2^* recombinase and induced *Pik3ca* gene deletion on P1. We then studied the effect of neonatal *Pik3ca* deletion in P21 pups by analyzing their cutaneous lymphatic capillaries, collecting vessels, and VEGFR3 expression. LYVE1 staining of the ears of *Pik3ca*-deleted pups revealed a sparse and chaotic lymphatic capillary network with loop-like structures and thinner lymphatic vessels (Figure 7A). The collecting lymphatic vessels were thinner than those in control mice, lacked SMC coverage, and had a reduced number of valves, as shown by αSMA and integrin α9 stainings, respectively (Figure 7B). VEGFR3 staining was reduced in the collecting lymphatic vessels and on the LEC surface in lymphatic capillaries exhibiting a perinuclear staining pattern that colocalized with the Golgi complex (Figure 7, C–E). These results demonstrate that *Pik3ca* deletion in LECs resulted in a phenotype similar to that seen with Tie1/-2 double-deletion in the collecting lymphatic vessels and with Ang2, Tie1, and Tie1/-2 deletion in the lymphatic capillaries, suggesting that PI3K is a key downstream regulator of Ang2/Tie signaling during postnatal lymphatic vessel development.

### PI3K inhibition reduces VEGFR3 levels in lymphatic malformations driven by an oncogenic PIK3CA mutation.

**Figure 8:**
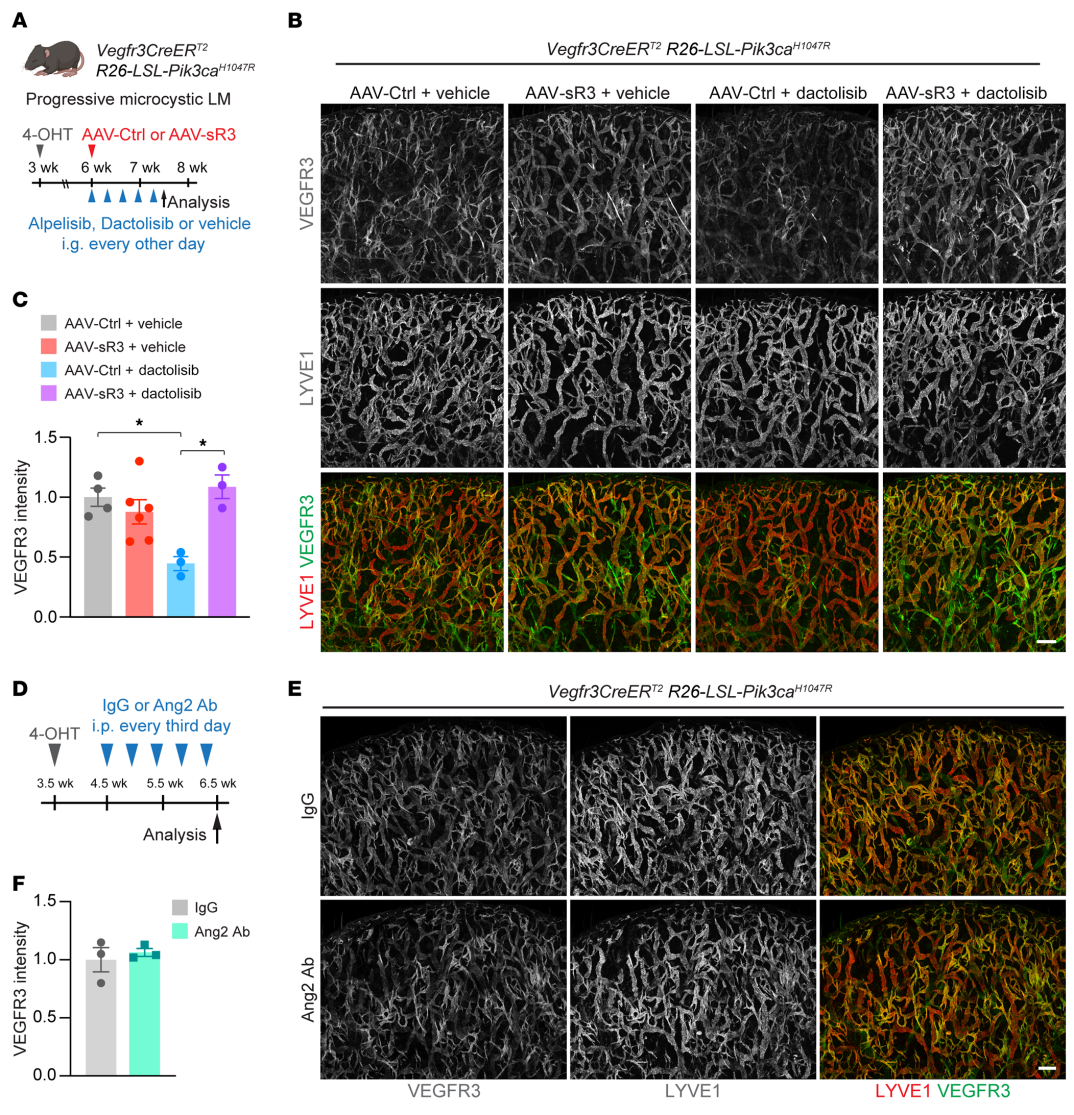
PI3K, but not Ang2, regulates VEGFR3 expression in *Pik3ca^H1047R^*-driven LMs. (**A**) Diagram showing the induction of progressive microcystic LM and its treatment by using the soluble VEGF-C trap (AAV-VEGFR3-Ig; AAV-sR3) combined or not with the PI3K pathway inhibitor dactolisib or alpelisib (BYL719). (**B**) LYVE1 and VEGFR3 staining of ears from 7.5-week-old *Vegfr3CreER^T2^* *R26-LSL-Pik3ca^H1047R^* mice treated with 4-OHT at 3 weeks of age, followed by treatment with dactolisib, AAV-sR3, and/or vehicle for 1.5 weeks. (**C**) Quantification of VEGFR3 in lymphatic vessels from *Vegfr3CreER^T2^* *R26-LSL-Pik3ca^H1047R^* mice treated with AAV-Ctrl plus vehicle (*n =* 4), AAV-sR3 plus vehicle (*n =* 6), AAV-Ctrl plus dactolisib (*n =* 3), or AAV-sR3 plus dactolisib (*n =* 3), normalized to control. (**D**) Diagram showing the induction of progressive microcystic LM and treatment with IgG or Ang2 Ab. (**E**) LYVE1 and VEGFR3 staining of ears from *Vegfr3CreER^T2^* *R26-LSL-Pik3ca^H1047R^* mice treated with 4-OHT at 3.5 weeks of age, followed by treatment with IgG or Ang2 Ab for 2 weeks. (**F**) Quantification of VEGFR3 in lymphatic vessels of *Vegfr3CreER^T2^* *R26-LSL-Pik3ca^H1047R^* mice treated with IgG or Ang2 ab (*n =* 3 per group), normalized to control. Scale bars: 200 μm. Data represent the mean ± SEM. *P < 0.05, by 1-way ANOVA with Bonferroni’s post hoc test for multiple comparisons (**C**) and 2-tailed Student’s *t* test (**F**).

Activating mutations in the *PIK3CA* gene are associated with lymphatic malformations (LMs) (44). We have previously reported that expression of the *Pik3ca^H1047R^* transgene in LECs results in progressive microcystic LMs that show increased expression of VEGFR3 in LECs (45). To further investigate the role of the PI3K pathway in VEGFR3 regulation, we induced lymphatic hyperplasia in 3-week-old *Vegfr3-CreER^T2^* *LSL-Pik3ca^H1047R^* pups (45) and then treated the mice with the PI3K pathway inhibitor dactolisib or alpelisib (BYL719) for 1.5 weeks (Figure 8A). Both the dual PI3K/mTOR inhibitor dactolisib and the selective PI3Kα inhibitor alpelisib resulted in decreased VEGFR3 expression in lymphatic vessels (Figure 8, B and C, and Supplemental Figure 13). To also inhibit the VEGF-C pathway, mice were treated with an adeno-associated viral (AAV) vector encoding a soluble VEGF-C/D-trap (AAV-sR3) (Figure 8A). The combined treatment rescued the downregulation of VEGFR3 caused by PI3K inhibition (Figure 8, B and C), suggesting that VEGF-C–induced internalization of VEGFR3 was required for the VEGFR3 downregulation.

Because the results indicated a role for Ang2/Tie signaling in the maintenance of VEGFR3 surface expression in the developing lymphatic vessels through regulation of PI3K activity, we hypothesized that high LEC-intrinsic PI3K activity in LMs is resistant to Ang2 inhibition. To test this, we induced lymphatic hyperplasia in 3.5-week-old *Vegfr3-CreER^T2^* *LSL-Pik3ca^H1047R^* pups, which were then treated with a control IgG or Ang2 Ab for 2 weeks (Figure 8D). Unlike PI3K inhibition, Ang2 Ab treatment did not reduce VEGFR3 expression in the lymphatic vessels (Figure 8, E and F). This result indicates that the Ang2 Ab could not overcome the effect of the oncogenic PI3K. In summary, the results show that, in a model of constitutively active PI3K signaling, the increase in VEGFR3 was resistant to Ang2 inhibition but sensitive to the blocking of PI3K.

### Transcriptomic changes in Tie1/-2–deleted and Ang2-inhibited LECs.

In order to determine the function of the Tie receptors in postnatal lymphangiogenesis at the transcriptional level, we compared the transcriptomes of cutaneous LECs from the Tie1- or Tie1/-2–deleted pups on P28 (deletion on P1) by single-cell RNA-Seq (scRNA-Seq) (Supplemental Figure 14A). Unsupervised clustering of 1377 sorted LECs from ear skin partitioned them into 5 clusters when visualized by uniform manifold approximation and projection for dimension reduction (UMAP) (Supplemental Figure 14B). These included capillary LECs (expressing *Lyve1*, *Reln*, *Piezo2*, *Slco2b1*, *Tppp3*, *Psck6*, and *Dlg1*), collector LECs (*Tgm2*, *Mmrn2*, *Plvap*, *Procr*, *CCdc3*, *Tsc22d1*, and *Bgn*), valve LECs (*Gdf10*, *Gjd3*, *Cldn11*, *Slc41a1*, and *Neo1*), proliferating LECs (*Mki67* and *Cenpa*), and LECs expressing IFN-induced transcripts (IFN LECs: *Irf7* and *Stat1*) (Supplemental Figure 14C and Supplemental Figure 15) (46). Analysis of the distribution of cells of the 4 genotypes (*Tie1^fl/fl^*, *Tie1^iΔLEC^*, *Tie1* *Tie2^fl/fl^*, and *Tie1* *Tie2^iΔLEC^*) in the different LEC clusters showed that the collecting vessel cluster was underrepresented among the Tie1/-2–deleted LECs, consistent with the immunofluorescence staining of the collecting lymphatic vessels (Figure 2, C and D, and Supplemental Figure 14, D and E). *Tie1* was more abundantly expressed than *Tek* (the gene encoding Tie2) in all LEC clusters, whereas *Tek* expression was highest in the collecting vessel cluster, correlating with the Tie1 and Tie2 immunostaining results (Supplemental Figure 16A). Tie1 deletion was associated with decreased Tie1 mRNA, whereas *Tek* mRNA was not altered upon Tie1 deletion. *Angpt2* mRNA expression was increased slightly in the Tie1-deleted mice and strongly in the Tie1/-2–deleted mice (Supplemental Figure 16A and Supplemental Table 1).

To determine whether Ang2 inhibition leads to similar changes, we isolated cutaneous LECs from pups treated with Ang2 Ab from P16 to P21. As in the Tie-deleted mice, the expression of Ang2 transcripts was also upregulated in mice treated with the Ang2 Ab (Supplemental Figure 16B and Supplemental Table 1). Overall, several genes were similarly regulated in the Tie1, Tie1/-2 double-deleted, and Ang2 Ab models (Supplemental Figure 17). In the Tie1-deleted and Ang2-inhibited pups, the strongest changes in gene expression were found in the capillary cluster, whereas in the Tie1/-2–deleted mice, the differentially expressed genes appeared also in the collecting and valve clusters, corresponding to the severe collecting lymphatic vessel phenotype observed in the double mutants (Supplemental Figure 17). Among genes common to all LEC subclusters, RNA encoding the major LEC transcription factor *Prox1* was not altered by the Tie deletions or Ang2 Abs (Supplemental Figure 18, A and B, and Supplemental Table 1). Despite the loss of VEGFR3 protein from the LEC surface in Tie1- or Ang2-deleted pups, VEGFR3 transcript levels were similar or slightly upregulated in the Ang2 Ab-treated and Tie1-deleted LECs, respectively (Supplemental Figure 18, A and B, and Supplemental Table 1). However, the expression of neuropilin 2 (*Nrp2*), a VEGFR3 coreceptor, and integrin α9 (*Itga9*), which has also been reported to bind VEGF-C, was downregulated in LECs from the Tie1/-2–deleted mice (Supplemental Figure 19 and Supplemental Table 1) (47, 48).

We also detected decreased VEGFR2 (*Kdr*) and increased *Lyve1* mRNA in LECs from the lymphatic vessels of the Tie1/-2–deleted and Ang2 Ab–inhibited mice, suggesting a failure of lymphatic vessel maturation (Supplemental Figure 19 and Supplemental Table 1) (34). The Tie1/-2–deleted mice also showed a significant decrease in the expression of other genes implicated in collecting lymphatic vessel formation, including the *Gata2* and *Foxc2* transcription factors and claudin 11, which are involved in lymphatic valve development (Supplemental Figure 19 and Supplemental Table 1) (49–51). Furthermore, the expression of several genes involved in intracellular trafficking and extracellular matrix biosynthesis was dysregulated in a similar manner in the Tie1-deleted, Tie1/-2–deleted, and Ang2 Ab–treated pups (Supplemental Figures 20 and 21). Thus, the transcriptomic effects of Tie receptor deletion and Ang2 inhibition in single LECs indicated profound changes in the expression of transcripts implicated in growth factor receptor signaling and lymphatic vessel formation and matrix biosynthesis.

### Tie receptors and Ang2 are required for VEGF-C–induced lymphangiogenesis in adult mice.

**Figure 9:**
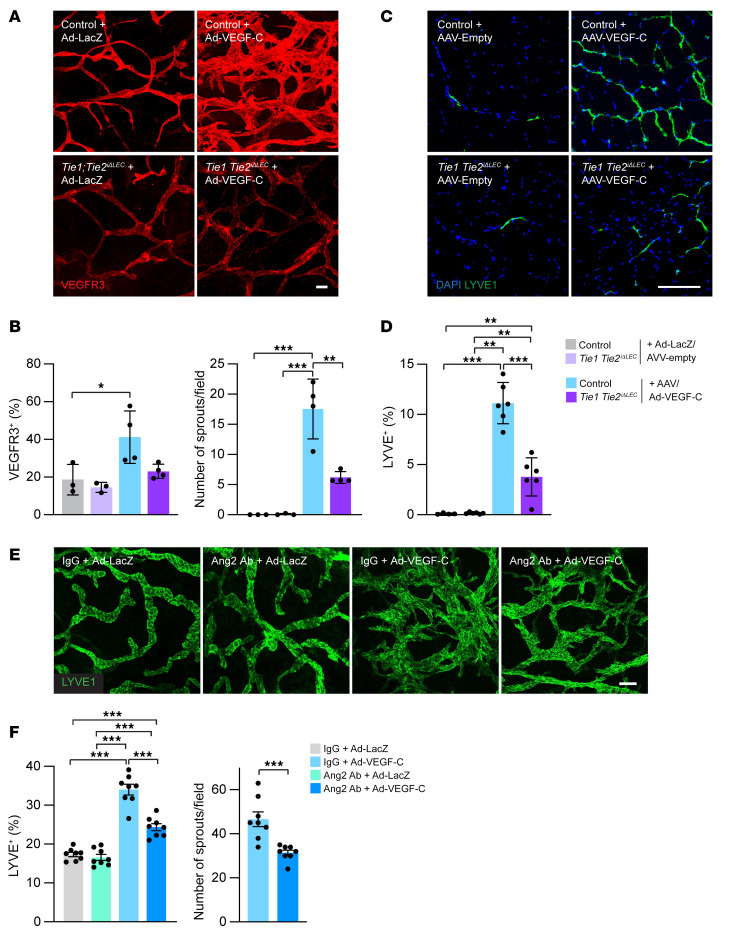
Ang2 and Tie receptors regulate VEGF-C–induced lymphangiogenesis in adult mice. (**A**) VEGFR3 staining of ear skin from control and Tie1/-2–deleted mice treated with Ad-LacZ or Ad–VEGF-C for 10 days. (**B**) Percentage of VEGFR3-positive area and number of sprouts per field (Ad-LacZ: *n =* 3 ears; Tie1;Tie2-del+Ad-LacZ: *n =* 3 ears; Ad-VEGF-C: *n =* 4 ears; Tie1;Tie2-del+Ad-VEGF-C: *n =* 4 ears). (**C**) LYVE1 staining of skeletal muscle from control and Tie1/-2–deleted mice treated with AAV-empty or AAV-VEGF-C for 4 weeks. (**D**) Percentage of LYVE1-positive area (AAV-empty: *n =* 4 muscles; Tie1;Tie2-del plus AAV-Empty: *n =* 6 muscles; AAV-VEGF-C: *n =* 6 muscles; Tie1;Tie2-del plus AAV-VEGF-C: *n =* 6 muscles). (**E**) LYVE1 staining of ear skin in mice treated for 1.5 weeks with Ad-VEGF-C, Ang2 Ab, or both. (**F**) Percentage of LYVE1-positive area and number of sprouts per field (*n =* 8 ears per group). Scale bars: 100 μm. Data represent the mean ± SEM. *P < 0.05, **P < 0.01, and ***P < 0.001, by 1-way ANOVA with Bonferroni’s post hoc test for multiple comparisons and 2-tailed Student’s *t* test.

Our previous studies have shown that, with the exception of the lacteal vessels in the gut and meningeal lymphatic vessels, the growth or survival of developing lymphatic vessels is dependent on endogenous VEGF-C only during the first couple of weeks postnatally (12, 52, 53). To determine whether the Tie receptors are required for VEGF-C–induced lymphangiogenesis in adult mice, we deleted both Tie1 and Tie2 in adult mice and then injected into their ear skin or tibialis anterior muscle, respectively, an adenoviral (Ad) or AAV vector encoding VEGF-C. We found considerably less VEGF-C–induced lymphangiogenesis in the Tie1/-2–deleted mice than in the control mice (Figure 9, A–D, and Supplemental Figure 22A). To determine whether Ang2 is also required for VEGF-C–induced lymphangiogenesis in adult mice, we injected Ad–VEGF-C into the ear skin and control IgG- or Ang2-blocking Abs intraperitoneally into the same mice. Analysis of the immunostained lymphatic vessels indicated that Ang2 inhibition reduced the VEGF-C–induced increase in lymphatic vessel area density and lymphatic sprouting (Figure 9, E and F, and Supplemental Figure 22B), showing that the Ang2 dependency was retained in adult lymphangiogenesis induced by VEGF-C. In summary, these results indicated that both the Tie receptors and endogenous Ang2 were required for VEGF-C–induced lymphangiogenesis in adult mice.

## Discussion

**Figure 10:**
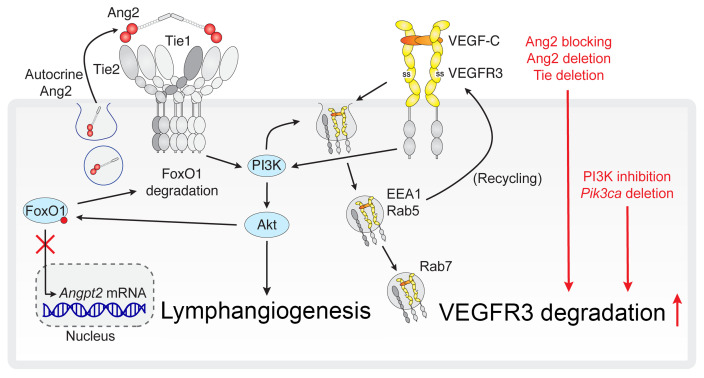
Schematic summary of how Ang2/Tie/PI3K signaling controls lymphangiogenesis via regulation of VEGFR3 cell-surface expression. Ang2 is shown as a tetramer of 2 asymmetric dimers binding to and activating the Tie2-Tie1 cluster (30, 66–68). Ang/Tie signaling promotes activation or PI3K and Akt, leading to inhibition of FoxO1 and its target genes, such as *Angpt2* (24, 27). In LECs, VEGF-C increased Ang2 release from stimulated cells and subsequent Tie2 and Akt activation. Disruption of Ang2/Tie signaling in Ang2 Ab–treated or Tie1- or Tie1/-2–deleted pups resulted in increased *Angpt2* gene expression, suggesting that Ang2 acts as an agonistic Tie2 ligand in LECs that promotes PI3K activation. VEGF-C induces internalization of VEGFR3 and its vesicular trafficking for degradation or recycling, which are regulated by PI3K (40–42). VEGF-C stimulation led to cotrafficking of VEGFR3 and Tie1 into EEA1/RAB5-positive early/sorting endosomes and, subsequently, to the RAB7-positive late endosomal degradative vesicle route. Our results show that inhibition or deletion of Ang2 or PI3K, or deletion of Tie receptors, promoted loss of VEGFR3 from the LEC surface and its increased degradation, leading to decreased lymphangiogenesis.

*ANGPT2* mutations and cosegregating *TIE1* variants were recently identified in patients with primary lymphedema (29, 30), which prompted us to investigate the specific roles of Ang2 and its Tie2-Tie1 receptor complex in the growth of lymphatic vessels postnatally and in adult tissues. Our results showed that Ang2/Tie/PI3K signaling was required for normal postnatal lymphatic vessel development via the maintenance of VEGFR3 expression on the surface of LECs. We detected Tie1 and VEGFR3 in close proximity to each other in cultured LECs, particularly at cell-cell junctions, and in the same intracellular vesicles following VEGF-C stimulation. *Pi3kca* deletion resulted in lymphatic vessel defects similar to those seen with deletion of Ang2 or both Tie1 and Tie2. Furthermore, in LMs driven by constitutively active *Pik3ca^H1047R^*, inhibition of PI3K activity, but not of Ang2, reduced VEGFR3 expression in a VEGF-C–dependent manner. Importantly, deletion of both Tie receptors or treatment with Ang2-blocking Abs also inhibited VEGF-C–induced lymphangiogenesis in adult mice. Overall, these results demonstrate that Ang2/Tie/PI3K signaling was required for VEGFR3 cell-surface expression and lymphangiogenesis (Figure 10).

### Function of the Ang2/Tie pathway in lymphatic vessel development and growth.

Unlike Ang1, Ang2 is a poor activator of Tie2 in BECs, but it can act as an agonistic ligand to activate Tie2 in LECs, because these cells lack VE-PTP, which dephosphorylates Tie2 in BECs (13, 16, 30). In the present study, blocking of Ang2, deletion of the *Angpt2* gene, or deletion of Tie1 during postnatal lymphatic vessel growth resulted in an abnormal lymphatic capillary network, whereas deletion of Tie2 alone was without effect, as previously reported (19, 34). This correlated with our finding of substantial Tie2 expression only in the collecting lymphatic vessels. Such regional specification of Tie expression occurs also in angiogenic sprouts of blood vessels, where the tip cells mainly express Tie1, which negatively regulates Tie2 surface presentation (21). In collecting lymphatic vessels, deletion of both Tie1 and Tie2 led to a more severe phenotype than deletion of either one alone, suggesting that Tie2 cooperates with Tie1 in the postnatal growth of collecting lymphatic vessels. The inhibition of cutaneous collecting lymphatic vessel enlargement after deletion of either Tie1 or Tie2 in transgenic pups expressing the endothelial cell–specific *Angpt2* transgene provided further evidence that Ang2 signals via the Tie receptors in LECs. These data indicate that Ang2 and Tie1 are the major regulators of postnatal lymphatic capillary network development and that deficiency of either one results in a similar phenotype, whereas Tie1 and Tie2 cooperate in the development of collecting lymphatic vessels.

### The Ang2/Tie pathway is required for cell-surface expression of VEGFR3.

We found that a lack of Ang2 or Tie1 led to decreased VEGFR3 staining on the surface of lymphatic capillary LECs, whereas the signal in the Golgi complexes of capillary LECs was preserved, consistent with continued biosynthesis of VEGFR3. In collecting lymphatic vessels, Ang2 deficiency or Ang2 blockage, or deletion of both Tie1 and Tie2 also reduced VEGFR3 staining intensity. Furthermore, Ang2 blockade reduced cell-surface expression of both Tie1 and VEGFR3, which were enriched at LEC cell-cell junctions in control LECs. Although a small fraction of Tie1 was proteolytically cleaved upon VEGF-C treatment, immunofluorescence staining showed that VEGF-C treatment led to colocalization of VEGFR3 and Tie1 first in RAB5- and EEA1-positive early/sorting endosomes and, subsequently, in the RAB7-positive late endosomal degradative vesicle route. The mature form of VEGFR3 was reduced in ear lysates from Ang2-inhibited, Ang2-deleted, and Tie1-deleted pups, confirming that Ang2/Tie signaling was required to sustain expression of the mature VEGFR3 protein. Changes in the VEGFR3 polypeptides induced by Ang2 Ab in vivo thus resembled those induced by VEGF-C in cultured LECs. The possibility that the attenuated Ang2/Tie receptor signaling tilts the balance of internalized VEGFR3 routing from the cell-surface recycling pathway to the late endosomal degradative pathway, needs to be addressed in future studies. Our results show that VEGFR3 and Tie1 associated with each other and at least partly shared the same internalization route, that both Tie1 and Ang2 were required to sustain cell-surface expression of the VEGF-C/D receptor VEGFR3 in capillary LECs, and that both Tie1 and Tie2 were required for VEGFR3 expression in collecting lymphatic vessels.

### Downstream of Ang2 and Tie receptors, PI3K regulates lymphatic vessel development and VEGFR3 expression.

The PI3K pathway downstream of the Tie receptor complex is involved in directing receptor tyrosine kinase–containing endocytic vesicles to recycling or degradation (13, 40). We found that Ang2 Ab treatment reduced VEGF-C–induced Akt phosphorylation downstream of PI3K, indicating impaired PI3K activation. Mechanistically, VEGF-C treatment resulted in Ang2 release from LECs and at least transiently increased Tie2 activation, which was blocked by the Ang2 Ab. These results are in line with our in vivo results showing that Tie2 was required for Ang2-induced collecting lymphatic vessel enlargement. Furthermore, these results provide a link between the 2 growth factor pathways by showing that VEGF-C–induced PI3K activation was boosted by autocrine Ang2 secreted by the LECs.

Previous work has indicated that the VEGFR3 protein is increased in the dense network of hyperbranched lymphatic vessels that develop in transgenic mice expressing a catalytically activated mutant *Pik3ca^H1047R^* (45). Furthermore, double-heterozygous deletion of VEGFR3 and the p110α catalytic subunit of PI3K results in severe lymphatic vessel defects and early postnatal lethality (54). This may be explained by our finding that postnatal p110α deletion from LECs resulted in reduced VEGFR3 surface expression and defective cutaneous lymphatic vessels, including collecting vessels that had reduced numbers of valves, as also found in the Tie1/-2 double-deleted pups. Consistent with this, in the LMs of *Pik3ca^H1047R^* mice, PI3K inhibitors reduced VEGFR3 protein levels in a VEGF-C–dependent manner. However, Ang2 Ab treatment did not affect VEGFR3 levels in the *Pik3ca^H1047R^* mice, as the constitutive mutant PI3K functions downstream of the Ang2-Tie receptor complex. Thus, although we found that Ang2 and PI3K similarly regulated VEGFR3 during postnatal lymphatic vessel development, only PI3K inhibition reduced the elevated VEGFR3 expression driven by the constitutively active PI3K. It has been reported that deletion of the *Akt1* gene in mouse embryos leads to atrophic lymphatic vessels and that rapamycin reduces VEGFR3 expression in LECs (55, 56). This corroborates the idea that the Ang2/Tie and VEGF-C/VEGFR3 pathways are linked via the PI3K/Akt/mTOR pathway. In summary, decreased PI3K activation via the Tie receptor complex was at least partially responsible for the observed loss of VEGFR3 from the cell surface that led to lymphatic vessel defects in the Tie-deleted pups.

### Transcriptomic changes upon loss of Ang2/Tie signaling.

Our scRNA-Seq of LECs isolated from Ang2 Ab–treated, Tie1-deleted, and Tie1/-2–deleted pups showed upregulation of *Angpt2* transcripts. As Tie-PI3K activation via Akt kinase activation and subsequent FoxO1 transcription factor inactivation inhibits Ang2 expression in endothelial cells (27), our results confirm that Ang2 acted as an agonistic Tie2 ligand in LECs. In addition, LECs isolated from Tie1/-2–deleted pups and Ang2 Ab–treated pups revealed downregulation of transcripts encoding Nrp2, which acts as a coreceptor of VEGFR3 and is required for VEGF-C–induced lymphatic vessel sprouting (48). Integrin α9, which binds VEGF-C, was also downregulated in the Tie1/-2–deleted pups, along with the Gata2 and Foxc2 transcription factors, which are essential for lymphatic valve development, and Cldn11, which marks lymphatic valves (47, 49–51). The fact that Gata2 and Foxc2 are both regulated by shear stress in lymphatic vessels suggests that the lymphatic vessels were dysfunctional in the Tie1/-2–deleted mice (2). Furthermore, constitutive Tie1 or Ang2 deficiency is known to result in impaired lymphatic function, and Tie1 has been suggested to be involved in mechanosensing of shear stress (14, 15, 17, 19, 57). Overall, these results indicate that the observed downregulation of VEGFR3, as well as the reduced expression of its coreceptors contributed to the observed deficiency of normal lymphangiogenesis and lymphatic maturation. Our scRNA-Seq results also implicated the Ang2/Tie pathway in the regulation of 2 key lymphatic transcription factors, extracellular matrix components, and intracellular vesicle trafficking. These results should be further investigated in future studies.

### Clinical implications of the crosstalk between Ang2/Tie and VEGF-C/VEGFR3 pathways.

Lymphatic vessels play critical roles in normal physiology, and their dysfunction leads to tissue edema. VEGF-C–induced lymphangiogenesis also participates in antitumor immune responses and tumor metastasis, inflammatory bowel diseases, and neurodegenerative and neuroinflammatory diseases (2, 3). Our study shows that Ang2/Tie signaling cooperated with the VEGF-C/VEGFR3 pathway involved in regulation of the development and growth of lymphatic capillaries and collecting vessels. Notably, our discovery that Ang2 and Tie1 were required for cell-surface VEGFR3 expression in the cutaneous lymphatic capillaries is consistent with previous findings that loss-of-function mutations of Ang2, and possibly of Tie1, predispose individuals to lymphedema and hydrops fetalis (29, 30, 58). In contrast, mutations of Ang1 and Tie2 have been found in patients with glaucoma who have increased intraocular pressure and compromised function of the Schlemm’s canal (SC), a lymphatic-like vessel that drains the intraocular fluid (59, 60). Additionally, a recent study showed that Tie1-deleted mice have a hypomorphic SC and elevated intraocular pressure (61). The inhibition of VEGF-C–mediated lymphangiogenesis that we report here could also be involved in the peripheral edema that has been reported in clinical trials using Ang2 inhibitors (62).

The finding that Ang2 blockade and Tie receptor deletion reduce VEGF-C–induced lymphangiogenesis in adult mice is of translational interest. A recent study showed the importance of agonistic Ang2/Tie2 signaling in maintaining lymphatic vessels in tumors and identified Ang2 as a therapeutic target in lymphatic metastasis, as acute treatment with Ang2-blocking Ab led to regression of the intratumoral lymphatic network (32). This finding is consistent with the atrophy and increased apoptosis we found in defective postnatal collecting lymphatic vessel development in Tie1/-2–deleted mice. Perhaps Ang2 can act as an autocrine survival factor in LECs, as it does in BECs (27). Given our findings, it would be interesting to test the VEGF-C/D trap in combination with the Ang2-blocking Ab or the PI3K inhibitor for suppression of lymphatic metastasis.

Whereas inhibition of lymphangiogenesis is desirable in LMs, lymphangiogenesis reactivation could be therapeutic in secondary lymphedema. Several strategies to therapeutically target lymphatic vessels are based on manipulation of the VEGF-C/VEGFR3 pathway (4, 63). Ad vector–based VEGF-C gene therapy is currently in a phase II clinical trial involving patients with breast cancer–associated secondary lymphedema (4, 7, 64). A more profound understanding of Ang2 and Tie1 signaling should be of clinical interest, as Ang2 mutations were already implicated in human primary lymphedema, and Tie1 may follow suit (29, 30). Perhaps increased autocrine Ang2 secretion by LECs could also improve VEGF-C–induced therapeutic lymphangiogenesis in lymphedema (7). The crosstalk between the VEGF-C and Ang signaling pathways will hopefully open new avenues for improved therapeutic manipulation of lymphangiogenesis.

## Methods

Further information can be found in the Supplemental Methods.

### Data availability.

All scRNA-Seq data have been deposited in the NCBI’s Gene Expression Omnibus (GEO) database (65) (GEO GSE202989).

### Statistics.

Results are expressed as the mean ± SEM. Statistical analysis was carried out with a 2-tailed Student’s *t* test or 1-way ANOVA, followed by Dunnett’s or Bonferroni’s post hoc test for multiple comparisons, using GraphPad Prism (GraphPad Software). A *P* value of less than 0.05 was considered statistically significant.

### Study approval.

Experimental procedures involving the use of mice were approved by the Project Authorization Board in Finland and the Uppsala Animal Experiment Ethics Board (Uppsala, Sweden). Mice were housed at a facility in individually ventilated cages with enrichment materials, following the guidelines of the Federation of European Laboratory Animal Science Associations.

## Author contributions

EAK conceived the study, designed experiments, conducted experiments, analyzed and interpreted results, and wrote the manuscript. AM, SKJ, AA, AP, YZ, SS, IL, LS, LA, ZS, EK, AS, IS, and AL designed and conducted experiments and analyzed and interpreted results. LE and YH supervised research. VML and HGA provided materials. KV, PS, and TM designed experiments, interpreted results, and supervised research. KA conceived the study, interpreted the results, and wrote the manuscript.

## Supplementary Material

Supplemental data

Supplemental video 1

## Version 1 06/28/2022

In-Press Preview

## Version 2 08/01/2022

Electronic publication

## References

[1] Pawlak JB, Caron KM (2020). Lymphatic programing and specialization in hybrid vessels. Front Physiol.

[2] Petrova TV, Koh GY (2020). Biological functions of lymphatic vessels. Science.

[3] Oliver G (2020). The lymphatic vasculature in the 21st Century: novel functional roles in homeostasis and disease. Cell.

[4] Xu W (2021). Lymphatic vasculature: an emerging therapeutic target and drug delivery route. Annu Rev Med.

[5] Fankhauser M (2017). Tumor lymphangiogenesis promotes T cell infiltration and potentiates immunotherapy in melanoma. Sci Transl Med.

[6] Song E (2020). VEGF-C-driven lymphatic drainage enables immunosurveillance of brain tumours. Nature.

[7] Hartiala P (2020). Phase 1 lymfactin study: short-term safety of combined adenoviral VEGF-C and lymph node transfer treatment for upper extremity lymphedema. J Plast Reconstr Aesthet Surg.

[8] Bazigou E, Makinen T (2013). Flow control in our vessels: vascular valves make sure there is no way back. Cell Mol Life Sci.

[9] Vaahtomeri K (2017). Lymphangiogenesis guidance by paracrine and pericellular factors. Genes Dev.

[10] Karkkainen MJ (2004). Vascular endothelial growth factor C is required for sprouting of the first lymphatic vessels from embryonic veins. Nat Immunol.

[11] Secker GA, Harvey NL (2015). VEGFR signaling during lymphatic vascular development: From progenitor cells to functional vessels. Dev Dyn.

[12] Karpanen T (2006). Lymphangiogenic growth factor responsiveness is modulated by postnatal lymphatic vessel maturation. Am J Pathol.

[13] Saharinen P (2017). Therapeutic targeting of the angiopoietin-TIE pathway. Nat Rev Drug Discov.

[14] D’Amico G (2010). Loss of endothelial Tie1 receptor impairs lymphatic vessel development-brief report. Arterioscler Thromb Vasc Biol.

[15] Qu X (2010). Abnormal embryonic lymphatic vessel development in Tie1 hypomorphic mice. Development.

[16] Souma T (2018). Context-dependent functions of angiopoietin 2 are determined by the endothelial phosphatase VEPTP. Proc Natl Acad Sci U S A.

[17] Zheng W (2014). Angiopoietin 2 regulates the transformation and integrity of lymphatic endothelial cell junctions. Genes Dev.

[18] Gale NW (2002). Angiopoietin-2 is required for postnatal angiogenesis and lymphatic patterning, and only the latter role is rescued by angiopoietin-1. Dev Cell.

[19] Dellinger M (2008). Defective remodeling and maturation of the lymphatic vasculature in angiopoietin-2 deficient mice. Dev Biol.

[20] Korhonen EA (2016). Tie1 controls angiopoietin function in vascular remodeling and inflammation. J Clin Invest.

[21] Savant S (2015). The orphan receptor Tie1 controls angiogenesis and vascular remodeling by differentially regulating Tie2 in tip and stalk cells. Cell Rep.

[22] Kontos CD (1998). Tyrosine 1101 of Tie2 is the major site of association of p85 and is required for activation of phosphatidylinositol 3-kinase and Akt. Mol Cell Biol.

[23] Kontos CD (2002). The endothelial receptor tyrosine kinase Tie1 activates phosphatidylinositol 3-kinase and Akt to inhibit apoptosis. Mol Cell Biol.

[24] Daly C (2004). Angiopoietin-1 modulates endothelial cell function and gene expression via the transcription factor FKHR (FOXO1). Genes Dev.

[25] Ghosh CC (2015). Drug repurposing screen identifies Foxo1-dependent angiopoietin-2 regulation in sepsis. Crit Care Med.

[26] Kim M (2016). Opposing actions of angiopoietin-2 on Tie2 signaling and FOXO1 activation. J Clin Invest.

[27] Daly C (2006). Angiopoietin-2 functions as an autocrine protective factor in stressed endothelial cells. Proc Natl Acad Sci U S A.

[28] Winderlich M (2009). VE-PTP controls blood vessel development by balancing Tie-2 activity. J Cell Biol.

[29] Michelini S (2020). TIE1 as a candidate gene for lymphatic malformations with or without lymphedema. Int J Mol Sci.

[30] Leppänen V-M (2020). Characterization of *ANGPT2* mutations associated with primary lymphedema. Sci Transl Med.

[31] Eklund L (2017). Angiopoietin-Tie signalling in the cardiovascular and lymphatic systems. Clin Sci (Lond).

[32] Gengenbacher N (2021). Timed Ang2-targeted therapy identifies the angiopoietin-tie pathway as key regulator of fatal lymphogenous metastasis. Cancer Discov.

[33] Holopainen T (2012). Effects of angiopoietin-2-blocking antibody on endothelial cell-cell junctions and lung metastasis. J Natl Cancer Inst.

[34] Shen B (2014). Genetic dissection of tie pathway in mouse lymphatic maturation and valve development. Arterioscler Thromb Vasc Biol.

[35] Jha SK (2018). Key molecules in lymphatic development, function, and identification. Ann Anat.

[36] Veikkola T (2003). Intrinsic versus microenvironmental regulation of lymphatic endothelial cell phenotype and function. FASEB J.

[37] Vanlandingham PA, Ceresa BP (2009). Rab7 regulates late endocytic trafficking downstream of multivesicular body biogenesis and cargo sequestration. J Biol Chem.

[38] Söderberg O (2006). et al. Direct observation of individual endogenous protein complexes in situ by proximity ligation. Nat Methods.

[39] Pajusola K (1994). et al. Signalling properties of FLT4, a proteolytically processed receptor tyrosine kinase related to two VEGF receptors. Oncogene.

[40] Bilanges B (2019). PI3K isoforms in cell signalling and vesicle trafficking. Nat Rev Mol Cell Biol.

[41] Deng Y (2015). Molecular controls of lymphatic VEGFR3 signaling. Arterioscler Thromb Vasc Biol.

[42] Lohela M (2008). Transgenic induction of vascular endothelial growth factor-C is strongly angiogenic in mouse embryos but leads to persistent lymphatic hyperplasia in adult tissues. Am J Pathol.

[43] Yabkowitz R (1999). Inflammatory cytokines and vascular endothelial growth factor stimulate the release of soluble tie receptor from human endothelial cells via metalloprotease activation. Blood.

[44] Castillo SD (2019). PIK3CA mutations in vascular malformations. Curr Opin Hematol.

[45] Martinez-Corral I (2020). Blockade of VEGF-C signaling inhibits lymphatic malformations driven by oncogenic PIK3CA mutation. Nat Commun.

[46] González-Loyola A (2021). FOXC2 controls adult lymphatic endothelial specialization, function, and gut lymphatic barrier preventing multiorgan failure. Sci Adv.

[47] Vlahakis NE (2005). The lymphangiogenic vascular endothelial growth factors VEGF-C and -D are ligands for the integrin alpha9beta1. J Biol Chem.

[48] Xu Y (2010). Neuropilin-2 mediates VEGF-C-induced lymphatic sprouting together with VEGFR3. J Cell Biol.

[49] Kazenwadel J (2015). GATA2 is required for lymphatic vessel valve development and maintenance. J Clin Invest.

[50] Sabine A (2015). FOXC2 and fluid shear stress stabilize postnatal lymphatic vasculature. J Clin Invest.

[51] Ortsäter H (2021). An inducible Cldn11-CreER(T2) mouse line for selective targeting of lymphatic valves. Genesis.

[52] Nurmi H (2015). VEGF-C is required for intestinal lymphatic vessel maintenance and lipid absorption. EMBO Mol Med.

[53] Antila S (2017). Development and plasticity of meningeal lymphatic vessels. J Exp Med.

[54] Stanczuk L (2015). cKit lineage hemogenic endothelium-derived cells contribute to mesenteric lymphatic vessels. Cell Rep.

[55] Zhou F (2010). Akt/Protein kinase B is required for lymphatic network formation, remodeling, and valve development. Am J Pathol.

[56] Luo Y (2012). Rapamycin inhibits lymphatic endothelial cell tube formation by downregulating vascular endothelial growth factor receptor 3 protein expression. Neoplasia.

[57] Qu X (2015). Tie1 is required for lymphatic valve and collecting vessel development. Dev Biol.

[58] Smeland MF Biallelic ANGPT2 loss-of-function causes severe early-onset non-immune hydrops fetalis. J Med Genet.

[59] Thomson BR (2017). Angiopoietin-1 is required for Schlemm’s canal development in mice and humans. J Clin Invest.

[60] Souma T (2016). Angiopoietin receptor TEK mutations underlie primary congenital glaucoma with variable expressivity. J Clin Invest.

[61] Du J (2022). Endothelial tyrosine kinase Tie1 is required for normal schlemm’s canal development-brief report. Arterioscler Thromb Vasc Biol.

[62] Hyman DM (2018). Phase I study of MEDI3617, a selective angiopoietin-2 inhibitor alone and combined with carboplatin/paclitaxel, paclitaxel, or bevacizumab for advanced solid tumors. Clin Cancer Res.

[63] Bui K, Hong YK (2020). Ras pathways on Prox1 and lymphangiogenesis: insights for therapeutics. Front Cardiovasc Med.

[64] https://clinicaltrials.gov/ct2/show/NCT03658967.

[65] Edgar R (2002). Gene Expression Omnibus: NCBI gene expression and hybridization array data repository. Nucleic Acids Res.

[66] Davis S (2003). Angiopoietins have distinct modular domains essential for receptor binding, dimerization and superclustering. Nat Struct Biol.

[67] Kim KT (2005). Oligomerization and multimerization are critical for angiopoietin-1 to bind and phosphorylate Tie2. J Biol Chem.

[68] Leppänen VM (2017). Structural basis of Tie2 activation and Tie2/Tie1 heterodimerization. Proc Natl Acad Sci U S A.

