# Supplementary material for: Lymphangiogenesis requires Ang2/Tie/PI3K signaling for VEGFR3 cell-surface expression
Source: J Clin Invest. 2022 Aug 1;132(15):e155478. doi: 10.1172/JCI155478 (PMC9337826; doi:10.1172/JCI155478)
Supplement: Supplemental data [file jci-132-155478-s067.pdf]

SUPPLEMENTAL MATERIAL FOR

Lymphangiogenesis requires Ang2/Tie/PI3K signaling for VEGFR3 cell surface expression

Emilia A. Korhonen, Aino Murtomäki, Sawan Kumar Jha, Andrey Anisimov, Anne Pink, Yan Zhang, Simon Stritt, Inam Liaqat, Lukas Stanczuk, Laura Alderfer, Zhiliang Sun, Emmi Kapiainen, Abhishek Singh, Ibrahim Sultan, Anni Lantta, Veli-Matti Leppänen, Lauri Eklund, Yulong He, Hellmut G. Augustin, Kari Vaahtomeri, Pipsa Saharinen, Taija Mäkinen and Kari Alitalo

SUPPLEMENTAL MATERIAL

Supplemental Methods

Mouse models and treatments. We used the *Prox1-CreER*^{T2} (1), *Cdh5-BAC-CreER*^{T2} (2), *Tie1*$^{fl/fl}$ (3, 4), *Tie2*$^{fl/fl}$ (5), *Ang2*$^{fl/fl}$ (6), *Ang2*$^{-/-}$ (7), *VE-cadherin-tTA* (8), *tetO-Ang2* (9), *Pik3ca*$^{fl/fl}$ (kindly provided by Mariona Graupera, Josep Carreras Leukaemia Research Institute, Barcelona (10)), *R26-LSL-Pik3ca*^{H1047R} (kindly provided by Dieter Saur, Technische Universität München, Munich (11)), and *Vegfr3CreER*^{T2} (kindly provided by Sagrario Ortega, CNIO, Madrid (12)) mouse lines. *Prox1-CreER*^{T2} *Tie2*$^{fl/fl}$ and *Prox1-CreER*^{T2} *Tie1*$^{fl/fl}$ *Tie2*$^{fl/fl}$ were maintained on C57BL/6J/N and *Ang2*$^{-/-}$ on C57BL/6N genetic background. All other strains were maintained on C57BL/6J genetic background. For induction of Cre mediated recombination in *Tie1*$^{fl/fl}$*, Tie2*$^{fl/fl}$ *or Tie1 Tie2*$^{fl/fl}$ pups, 4-hydroxytamoxifen (4-OHT), dissolved in ethanol (25 mg/ml), was administered to postnatal pups by intragastric injections (2.5 µl, 62.5 µg) at P1-P5 or P1-P4. For induction of Cre recombination in *Ang2*$^{fl/fl}$ pups, tamoxifen free base was administered to postnatal pups by intragastric injections (60 µg) at P1-P4 (Supplemental Figure 5) or 4-OHT was dissolved in ethanol, diluted in corn oil and administered by intraperitoneal injections (167 µg) at P12-P14 (Figure 6B). For induction of Cre recombination in *Pik3ca*$^{fl/fl}$ pups, 50 µg of 4-OHT, dissolved in ethanol, was administered by intragastric injection at P1 and P2. Lymphatic overgrowth was induced in 3-week-old *Vegfr3CreER*^{T2} *R26-LSL-Pik3ca*^{H1047R} mice by intraperitoneal injection of 100 µg of 4-OHT, dissolved in peanut oil (1mg/ml), or topical application of 100 µg of 4-OHT, dissolved in acetone (5 mg/ml), to the dorsal side of each ear. For experiments with Ang2 overexpression, Ang2 expression was repressed until E12.5 by administration of 2 mg/ml of tetracycline in 5% sucrose in the drinking water of pregnant females. For treatment with blocking Abs, pups or *Vegfr3CreER*^{T2} *R26-LSL-Pik3ca*^{H1047R} mice were injected subcutaneously or intraperitoneally every third day from P1 to P21 (or from P12 to P17 in Figure 6A and P16 to P21 for 10X scRNAseq) or 4.5 weeks to 6.5 weeks of age (or at indicated time points) with 20 mg/kg Ang2 blocking Ab (18E5, a kind gift from Eli Lilly&Co) or mouse IgG1 control Ab (13). For all the experiments involving genetic mouse models, control refers to Cre-negative floxed littermate. Thus, the comparisons between genotypes were always done between Cre-negative floxed controls and Cre-positive floxed knockout littermates (*Tie1*$^{fl/fl}$ vs. *Prox1-CreER*^{T2} *Tie1*$^{fl/fl}$, *Tie2*$^{fl/fl}$ vs. *Prox1-CreER*^{T2} *Tie2*$^{fl/fl}$, *Tie1 Tie21*$^{fl/fl}$ vs. *Prox1-CreER*^{T2} *Tie1 Tie2*$^{fl/fl}$, *Ang2*$^{fl/fl}$ vs.

Prox1-CreER^{T2} *Ang2*$^{fl/fl}$, *Ang2*$^{fl/fl}$ vs. *Cdh5-BAC-CreER*^{T2} *Ang2*$^{fl/fl}$). For the experiments with *Pik3ca*$^{fl/fl}$ mouse line, Cre-negative floxed pups or *Prox1-CreER*^{T2} *Pik3ca*$^{fl/+}$ were used as controls. For experiments with Ang2 overexpression, Cre-negative floxed littermates without or with a single transgene (*VE-cadherin-tTA* or *tetO-Ang2)* were used as controls.

For inhibition of VEGF-C signaling in *Vegfr3CreER*^{T2} *R26-LSL-Pik3ca*^{H1047R} mice, we used adeno-associated viral (AAV) vector encoding VEGF-C trap, consisting of the ligand binding domains 1–4 of VEGFR3, fused to the IgG Fc domain (AAV9-mVEGFR3$_{1–4}$-Ig) and control AAVs that encoded the domains 4-7 of VEGFR3 (AAV9-mVEGFR3$_{4-7}$-Ig) (14). At 5 weeks of age, the mice received a single intraperitoneal injection of 1×10^{11} virus particles diluted in 100 µl PBS. Dactolisib (Selleckchem S1009) was dissolved in N-methyl-2-pyrrolidone (NMP, Sigma 328634)/PEG300 (Sigma 202371) (1:9) at 8.3 mg/ml and Alpelisib (BYL719, MedChemExpress HY-15244) was dissolved in 0.5% carboxymethylcellulose (CMC, Sigma C5678) at 8.3 mg/ml. Dactolisib or Alpelisib was administered by oral gavage every second day, starting at 6 weeks of age for 5 times at the dose of 50 mg/kg body weight. NMP/PEG or 0.5% CMC was used as a control.

To study the effect of Ang2 Abs on VEGF-C mediated lymphangiogenesis, $6x10^8$ pfu of Ad-VEGF-C or control adenovirus expressing LacZ (adenoviruses kindly provided by Seppo Ylä-Herttuala (A.I. Virtanen Institute for Molecular Sciences, Kuopio) was injected into the ears of C57BL/6J mice (Janvier) (15). Mice were injected i.p. every third day with 20 mg/kg Ang2 blocking or control Ab and sacrificed after 10 days. To study the effect of Tie1 and Tie2 on VEGF-C mediated lymphangiogenesis, *Tie1* $^{fl/fl}$ *Tie2*$^{fl/fl}$ and *Prox1-CreER*^{T2} *Tie1* $^{fl/fl}$ *Tie2*$^{fl/fl}$ adult mice were treated by daily administration of tamoxifen by oral gavage (2 mg/mouse/d) for 5 days. The mice were then injected with AAV9-VEGF-C (10^{11} vp/muscle) or AAV9-Empty into the skeletal muscle (tibialis anterior) (16). 2.5 weeks after the AAV injections Ad-VEGF-C or control Ad-LacZ ($12x10^8$ pfu) was injected into the ears of the same mice. The mice were sacrificed after additional 10 days and the muscles and ears were harvested. The muscles were frozen in O.C.T. for sectioning and ears processed for whole-mount staining.

Cell culture and treatments. LECs (HDLEC-c, Human Dermal Lymphatic Endothelial Cells, PromoCell) were grown either in EBM-2 supplemented with EGM-2 MV SingleQuot Suppl&Growth Factors (Lonza) or supplemented with MV2 (PromoCell C-22121) on gelatin (0.1%) or fibronectin (1

μg/ml)-coated culture plates, ibidi coverglass bottom chambers or coverslips. LEC culture medium was supplemented with 25 ng/ml VEGF-C (17).

For lentiviral EGFP-RAB5C expression, LECs were transduced with viral particles for 5 h in the presence of polybrene (8μg/ml). Prior to treatment with VEGF-C, cells were starved for 5 h in basal MV2 media containing 0.5% FCS and fixed 72 h after transduction. For cell surface staining of VEGFR3 and Tie1, the cells were incubated overnight without VEGF-C and then incubated for 2 h in 0.5% FCS containing media. Next, the human anti-Ang2 Ab (18) or control human IgG were added for 30 min, or LECs were stimulated with VEGF-C (100 ng/ml) for 20 min, followed by cell surface immunostaining on ice.

For analysis of VEGFR3, pAkt and pERK, LEC cultures were grown to confluency in MV2 medium (PromoCell C-39221) containing the growth supplements. The medium was then replaced with MV medium (PromoCell C-39220), and the next day cells were treated with anti-Ang2 Ab for 2 h, followed by VEGF-C (100 ng/ml) stimulation for 0.5, 1 or 3 h. For analysis of cell surface VEGFR3 by Western blotting the cultures were treated with trypsin (Euroclone ECM0920D) for 10 min at 37°C, whereafter the cells were centrifuged and lysed for gel electrophoresis. For analysis of Tie2 phosphorylation, the LECs were treated with anti-Ang2 Ab for 2 h followed by VEGF-C stimulation for 45 min.

For live-microscopy, mouse monoclonal anti-human Tie1 (7E8G9F6, (19)) and VEGFR3 (9D9, ReliaTech #101-M36 or Novus Biologicals NBP1-18651) Abs were labeled with Alexa Fluor 488 and Alexa Fluor 568 protein labeling kits (Invitrogen A10235 and A10238, respectively). LECs were starved in 0.5% FCS containing media followed by incubation of cells with anti-hTie1-488, anti-hVEGFR3-568 and anti-hVE-cadherin-647 (BD Biosciences 561567) on ice for 30 min. Next cells were washed once with 0.5% FCS media and then media with 0.5% FCS and 100 ng/ml VEGF-C was added on the LECs. LECs were transferred to the Nikon Ti-E2 microscope equipped with an atmospheric chamber (37°C and 5% CO_2) microscope and imaging was started immediately upon determination of the imaging locations and the focus plane.

EGFP-RAB5C plasmid construction. We used 5’-GGGGACAAGTTTGTACAAAAAAGCAGGCTTAGGATCCATGGCGGGTCGGGGAGGCGC-3’

and 5’-GGGGACCACTTTGTACAAGAAAGCTGGGTTGGATTCAGTTGCTGCAGCACTG-3’ primers to sub-clone RAB5C (clone ID 100067131, ORFeome Library; Genome Biology Unit supported by HiLIFE and the Faculty of Medicine, University of Helsinki, and Biocenter Finland) with 5’ BAMHI restriction site to pENTR221 vector, resulting in pENTR221-BAMHI-RAB5C vector. Next we conducted a PCR of EGFP with the following primers that included the BAMHI site 5’-TAGTCTGGATCCTCCACCATGGTGAGCAAGGGCG-3’ and 5’-ATCAACGGATCCAGAACCACCACTTCCACCG-3’. The BAMHI cut PCR-product was then ligated to BAMHI cut pENTR221-BAMH1-RAB5C plasmid, resulting in pENTR221-EGFP-BAMHI-RAB5C. After sequence and restriction digestion analyses the EGFP-RAB5C was transferred to pLenti6.3. vector via Gateway LR-reaction and the correct clones were identified with restriction analyses. The viruses were produced as previously described (20).

Receptor internalization. Confluent LEC monolayers were grown overnight in media without VEGF-C and then starved in 0.5% FCS media for 2 h. For surface labeling of VEGFR3 and Tie1, coverslips were incubated with anti-hTie1-Alexa488 and anti-hVEGFR3-Alexa568 in 1% BSA diluted in endothelial basal media (EBM-2) on ice for 30 min. Coverslips were rinsed three times with blocking buffer followed by stimulation with VEGF-C (100 ng/ml) for 20 min or 1 h at 37ºC. Coverslips were washed on ice with blocking buffer, Dulbecco's Phosphate Buffered Saline (DPBS), 4 x 5 min with acid wash (100 mM glycine, 1% BSA in DPBS, pH 2.5) and finally 3 times with DPBS. Coverslips were fixed with 4% paraformaldehyde (PFA) on ice and washed twice with DPBS and twice with PBS and mounted using Abcam mounting medium containing DAPI (Abcam ab104139). For the experiment with 1 h VEGF-C stimulation, coverslips were also permeabilized and stained for RAB7 (Cell Signaling 9367S).

Immunofluorescence staining. For whole-mount staining, the ears were fixed in 1-4% PFA for 1 h at room temperature (RT) or in 4% PFA for overnight at 4°C, followed by several washes with PBS. The tissues were treated with Donkey immunomix blocking buffer (5% donkey serum, 0.2% BSA, 0.05% NaN3 and 0.3% Triton X-100 in PBS) for 1-2 h, incubated with primary Abs diluted in the blocking buffer, at 4°C for 1-3 days or overnight at RT, and washed with 0.3% Triton X-100 in PBS. For detection of the primary Abs, the whole mounts were incubated with Alexa fluorochrome conjugated secondary Abs (Life Technologies) diluted in washing solution overnight at 4°C or RT. For cell surface staining,

the tissues were blocked in 1% BSA in PBS for 1-2 h and incubated in primary Abs diluted in the blocking buffer at 4°C for 1-3 days, washed with PBS and incubated overnight with secondary Abs diluted in the blocking buffer at 4°C. The samples were mounted with Vectashield mounting medium (Vector Labs). For the ear skins from *Pik3ca*$^{fl/fl}$ and *R26-LSL-Pik3ca*^{H1047R} mice, whole-mount tissue was fixed in 4% PFA at RT for 2 h followed by permeabilization in 0.3% Triton X-100 in PBS (PBST) for 10 min and blocking in PBST plus 3% milk for 1-2 h. The ear skins were incubated with primary Abs at 4°C overnight in blocking buffer and washed in PBST before incubation with fluorescence-conjugated secondary Abs (Jackson ImmunoResearch) in blocking buffer for 2 h at RT. For cell surface staining, the ear skins were blocked in PBS plus 3% milk for 1-2 h, and incubated with Abs at 4 °C overnight in blocking buffer, washed with PBS and incubated with fluorescence-conjugated secondary Abs in blocking buffer for 2 h at RT. Stained samples were then washed and mounted in Mowiol. The following Abs were used for whole-mount stainings: VE-Cadherin (BD 555289), VE-Cadherin (eBioscience 14-1441-85), Tie1 (R&D AF619), Tie2 (R&D AF762), LYVE1 (R&D MAB2125), LYVE1 (Reliatech 103-PA50AG), Podoplanin (DSHB 8.1.1.), Podoplanin (Invitrogen 13-5381-82), Integrinα9 (R&D AF3827), SMA-Cy3 (Sigma C6198), cleaved Caspase3 (Cell Signaling 9661), VEGFR3 (R&D AF743), CD31 (Millipore MAB1398Z), Prox1 (AngioBio 11-002P), Prox1 (R&D AF2727) and GOLPH4 (Abcam ab28049).

For immunofluorescence of skeletal muscle sections (10 um), the sections were fixed in 4% PFA for 10 min, permeabilized with 0.5% Triton X-100 in PBS for 5 min and blocked in TNB (100 mM Tris, 150 mM NaCl, 0.5% blocking reagent, PerkinElmer FPI1020) for 30 min. LYVE1 (R&D MAB2125) Ab was incubated in TNB overnight, rinsed with TNT (100 mM Tris, 150 mM NaCl, 0.05% Tween-20) and incubated in secondary Abs in TNB for 1 h.

For immunofluorescence staining of LECs, coverslips or ibidi coverglass bottom chambers were fixed in 4% PFA for 10 min at RT and washed with PBS. Coverslips were permeabilized with 0.1% Triton-X in PBS for 5 min and blocked with 1% BSA-PBS, whereas ibidi coverglas bottom chambers were permeabilized with 0.15% Triton-X in PBS for 15 min and blocked in 3% BSA-PBS. Blocked coverslips and ibidi coverglass bottom chambers were stained using primary Abs for 30-60 min at RT or overnight at 4°C, washed with PBS, stained with secondary fluorescently conjugated Abs (Life Technologies) when appropriate, and mounted using Vectashield mounting medium containing DAPI (Vector Labs). For cell

surface staining, coverslips were blocked on ice for 5 to 10 min in 1%BSA-EBM-2, incubated in primary Abs diluted in the blocking buffer on ice for 30 min, washed twice with cold DPBS and fixed in 4% PFA on ice for 20 min. For detection of the primary Abs, the coverslips were stained with secondary fluorescently conjugated Abs (Life Technologies) at RT. The following Abs were used for LEC stainings: VEGFR3 (R&D AF349), 9D9/VEGFR-3 (ReliaTech 101-M36), and Tie1 (R&D AF619), RAB7 (Cell Signaling, 9367S) and EEA1 (Cell Signaling 3288).

Proximity ligation assay (PLA). We used Duolink® flowPLA Detection Kit - Red (Sigma-Aldrich DUO94001) and anti-mouse PLUS (DUO92001) and anti-human MINUS (DUO92021) PLA probes. LECs in MV2 medium with supplements were cultured on coverslips until about 90% confluent, then incubated in MV medium (with supplements) for 24 h, fixed (4% PFA, 10 min), permeabilized (0.3% Triton X-100 in PBS, 5 min) and blocked with Duolink blocking solution (DUO82007) for 1 h. Primary Abs (all abs – at 5 ug/ml final concentration in 2.5% NDS, 0.05% Tween-20, 0.1% NaN3) were incubated on the coverslips overnight. The coverslips were then rinsed 2 times with PBS and subjected to the PLA reaction development according to the manufacturer's guidelines. After short rinsing, the coverslips were stained for Prox1, post-fixed and mounted using Vectashield. The following primary or control Abs were used: human anti-hAng2 (18), human anti-hTie1 Dx2240 (available through Creative Biolabs), mouse anti-human Notch1 (Abcam ab44986), mouse monoclonal anti-human VEGFR3 9D9 (Novus Biologicals NBP1-18651 or ReliaTech 101-M36), human anti-human VEGFR3 3C5 (21), hIgG, or mIgG.

Image acquisition and quantification. All confocal images represent maximum intensity projections of Z-stacks of single tiles or multiple tile scan images. Images were acquired using Zeiss 710, Zeiss 780, Zeiss 880, Leica SP8 or Andor Dragonfly spinning disk confocal microscopy. Branchpoints and vessel overlaps in the lymphatic capillary network of ventral ears were calculated manually using Fiji ImageJ and the diameter was measured using Autotube (22). Quantification of SMA coverage, number of valves and the collecting vessel diameter was measured using Fiji ImageJ. For quantification of SMA coverage, the collecting lymphatic vessel length was measured from the base to the tip and the vessel was divided into two parts: region 1 (proximal) and region 2 (distal). Quantification of lymphatic collecting vessel diameters was measured from the whole collector or separately from region 1 and region 2. Quantification of collector VEGFR3, cell surface VEGFR3, Tie1, and Tie2 staining intensities in the ears of postnatal pups was done by measuring the mean intensity in the vessels using Fiji ImageJ.

Quantification of VEGFR3 intensity in the lymphatic capillaries and in the Golgi complex was measured with Imaris. Quantification of VEGFR3 staining intensity using tile scan images of adult ear skin was done by measuring corrected total cell fluorescence (CTCF) = integrated density – (area of selected cell × mean fluorescence of background readings) using ImageJ. Lymphatic vessel areas were measured with threshold in Fiji ImageJ or with Autotube and sprouts were quantified manually. For Ad-VEGF-C+Ang2 ab experiment (Figure 9, E-F), sprout counts were only included for Ad-VEGF-C and Ad-VEGF-C+Ang2 ab groups since lymphatic vessels in the other groups did not contain sprouts. Quantification for PLA experiments was performed by counting spots (=PLA events) per field of view. Quantification of Tie1 and VEGFR3 positive surface area percentage in LECs was done by measuring integrated fluorescence intensity using ImageJ and normalized to cell number (nuclei). Internalized VEGFR3 and VEGFR3/Tie1 vesicles were quantified using ImageJ and object-based colocalization approach. After segmentation and generation of binary masks of each channel, the masks were combined by Process>Math>AND command to find the fractions of overlapping pixels. The colocalizing vesicles were quantified by Analyse Particles command and normalized to total cell number (nuclei).

Western blotting. Precipitated cells or cells in culture vessels were lysed in 0.5% Triton X-100, 0.5% NP-40, aprotinin (10 ug/ml), leupeptin (10 ug/ml), PMSF (0.5 mM), NaF (50 mM), Na3VO4 (5 mM), β-Glycerophosphate (20 mM) and Sodium pyrophosphate (5 mM) in PBS. Insoluble cell debris was removed by centrifugation and total lysates were separated in SDS-PAGE and transferred to PVDF membrane (Millipore). For experiment shown in Figure 6H, total Tie2 protein was immunoprecipitated using mixture of goat anti-human Tie2 (R&D AF313) and mouse anti-Tie2 (Millipore 05-584) and protein G sepharose (Cytiva 17061801), followed by SDS-PAGE and transfer to PVDF membrane. Blots were probed with primary Abs, then HRP-conjugated secondary Abs (Dako), followed by ECL detection with the SuperSignal West Pico Chemiluminescent Substrate or SuperSignal West Femto Maximum Sensitivity Substrate (Thermo Scientific). Signals from Western blots were captured on film or imaged with Odyssey FC (LI-COR). For reprobing, the membranes were stripped using the Re-Blot Plus Strong Solution (Millipore) and re-probed with the respective primary Ab. Goat anti-human VEGFR3 (R&D AF349), rabbit anti-pAkt (S473) and rabbit anti-Akt (Cell Signaling Technology 9271 and 9272, respectively), rabbit anti-pERK and rabbit anti-ERK (Cell Signaling Technology 9101 and 9102, respectively), goat anti-human Ang2 (R&D AF623), goat anti-human Tie2 (R&D AF313), mouse anti-

p-Tyr (clone PY99; Santa-Cruz sc-7020) and mouse anti-HSC70 (Santa-Cruz sc-7298) Abs were used for immunoblotting.

Ear skin was lysed in tissue lysis buffer (1 mM PMSF, 2 mM Na3VO4, 50 mM NaF, 1× protease inhibitor cocktail without EDTA (Roche Applied Science), 50 mM Tris pH 7.4, 150 mM NaCl, 25 mM β-glycerolphosphate, 2 mM sodium pyrophosphate, 1 mM EDTA and 1% NP-40) and homogenized using high density zirconium oxide beads (Biotop MB2Z015) followed by sonication on ice. Insoluble tissue debris was removed by centrifugation. 40-50 ug of total lysate per sample was separated in SDS-PAGE and transferred to PVDF membrane (Millipore), probed with primary Abs followed by incubation with HRP-conjugated secondary Abs (Dako). ECL detection was done using the SuperSignal West Pico Chemiluminescent Substrate or SuperSignal West Femto Maximum Sensitivity Substrate (Thermo Scientific) followed by imaging with Odyssey FC (LI-COR). VEGFR3 (R&D AF743), Ang2 (R&D AF7186), Prox1 (R&D AF2727), HSC70 (Santa Cruz sc-7298) and β-actin (Santa Cruz sc47778) Abs were used for immunoblotting.

Enzyme-linked immunosorbent assay (ELISA). LEC cultures were grown as described above. Just before the stimulation the media in all cultures was replaced with fresh one and the cells were stimulated with VEGF-C (100 ng/ml) for various periods of time. Supernatants were collected and Ang2 or soluble Tie1 (sTie1) were measured using ELISA kits (Quantikine DANG20 for human Ang2 and R&D DuoSet DY5907 for sTie1).

RT-qPCR of isolated LECs and BECs. LECs and BECs were isolated from mouse ear skins and sorted as described for 10x scRNA-Seq (below). Collected LECs and BECs were centrifuged for 6 min at 300g, 4°C, and supernatants were discarded. Cells were then lysed and prepared for the RT reaction using the TaqMan Fast Advanced cells-to-Ct kit (Thermo Fisher A35377) according to the manufacturer's protocol. Briefly, cell pellets were resuspended in 100 µl of lysis solution and lysis was quenched with 10 µl of the stop solution. 22.5 µl of lysate was used per RT reaction, which consisted of heating to 37°C for 30min, followed by 95°C for 5 min. 20 µl q-PCR reactions were performed using 9 µl of RT material, Taqman probes (Prox1: Mm00435969_m1; Tie1: Mm01180904_g1; Tek: Mm00443243_m1; GAPDH: 4352932E) and Taqman Fast Advanced MasterMix. q-PCR was run at 50°C for 2 min, 95°C for 20 sec,

followed by 40 cycles of 95°C for 1 sec, 60°C for 20 sec. Results were analyzed by the ΔΔCt method and normalized to their littermate controls.

scRNA-Seq analysis of LECs. Control, Tie1-deleted and Tie1/-2-deleted samples were analyzed using Smart-seq and IgG and Ang2 Ab treated samples with 10x. Ear skins of euthanized mice were minced and dissociated by incubation with 5 mg/ml collagenase II, 0.2 mg/ml DNase I and 0.2% FBS in PBS for 15 min at 37°C and 750 rpm. Digests were quenched by the addition of EDTA (final concentration 2 mM), subsequently filtered through a 40 µm filter and washed for 5 min at 300 g and 4°C with FACS buffer (PBS, 0.5% FBS, and 2 mM EDTA). Fc-receptor binding was blocked by incubation with rat anti-mouse CD16/CD32 (clone 93, 0.5 µg/ml) for 10 min on ice. Thereafter, samples were incubated with fluorophore-conjugated anti-CD31 (clone 390, 1.0 µg/ml), anti-PDPN (clone 8.1.1 1 µg/ml), anti-CD45 (clone 30-F11, 4 µg/ml), anti-CD11b (clone M1/70, 4 µg/ml) and anti-TER-119 (clone TER-119, 4 µg/ml) Abs for 20 min (Smart-seq2) or 30 min (10x) on ice. Samples were filtered through a 40 µm filter and supplemented with 1 µM SytoxBlue (Smart-seq2) or DAPI (10x) to label dead cells. For Smart-seq2 live $CD31^+$ $PDPN^+$ $CD45^-$ $CD11b^-$ $Ter119^-$ LECs were sorted on a BD FACS Aria III. For 10x live $CD31^+$ $PDPN^+$ $CD45^-$ $CD11b^-$ $Ter119^-$ LECs and $CD31^+$ $PDPN^-$ $CD45^-$ $CD11b^-$ $Ter119^-$ BECs were sorted on a BD Influx and pooled.

Smart-seq2 library preparation and sequencing were performed as described previously (23, 24). The sequencing reads were mapped to the mouse reference genome GRCm38 (mm10) with TopHat (version 2.1.1). Duplicated reads were removed using samtools software (version 0.1.18). The raw read counts for each gene were then calculated using featureCounts function from the Subread package (version 1.4.6-p5).

For 10x, cell concentration and viability were determined in a Luna-FX7 with Acridine Orange/Propidium Iodide Stain (F23001, Logos Biosystems). Approximately 4,000 sorted cells were further processed for scRNA-Seq using the Chromium Next GEM Single Cell 3′ Reagent Kit, version 3.1 (Dual Index), and the Gel Bead Kit, version 3.1 (10× Genomics), according to the manufacturer's instructions. Libraries were sequenced to an average depth of 50,000 reads on a NovaSeq 6000 Sequencing System (Illumina), and raw sequencing data were processed with Cell Ranger v2.1.1 pipelines (FiMM).

Count matrix was further processed using Seurat package (v4.0.1), according to the instructions on the Satija lab website (https:// satijalab.org/Seurat). All default parameters were unchanged unless otherwise specified for data analysis. For quality control, genes that were expressed in less than three cells and cells with less than 200 genes were excluded from the analysis. The counts were log normalized and 2000 highly variable genes were selected for further analysis. The individual R object were then merged and dimensionally reduced with Canonical Correlation Analysis (CCA) method in "FindIntegrationAnchors" and "IntegrateData" functions. The integrated data was dimensionally reduced using Principal Component Analysis (PCA), and "FindConservedMarkers" function was used to identify cluster markers for each cell clusters. For 10x data, endothelial cells were clustered using 35 dimensions and a 0.6 resolution. Clusters were visualized using Uniform Mannifold Approximation and Projection (UMAP) plot. The differentially expressed genes across cells in each cluster were identified using the "FindMarker" function. Since we were interested in LECs, clusters containing LEC populations were extracted and further processed. For Smart-seq2 and extracted LECs from ECs in 10x, clustering of cells was performed using 15 dimensions and a 0.4 resolution. Clusters were annotated using established LECs markers.

Heatmap of the top 10 selected differentially expressed genes for each cluster were generated and visualized using "DoHeatmap" function in Seurat package.

Supplemental References

1. Bazigou E, et al. Genes regulating lymphangiogenesis control venous valve formation and maintenance in mice. *J Clin Invest.* 2011;121(8):2984–2992.

2. Okabe K, et al. Neurons limit angiogenesis by titrating VEGF in retina. *Cell.* 2014;159(3):584–596.

3. D'Amico G, et al. Tie1 deletion inhibits tumor growth and improves angiopoietin antagonist therapy. *J Clin Invest.* 2014;124(2):824–834.

4. D'Amico G, et al. Loss of endothelial tie1 receptor impairs lymphatic vessel development-brief report. *Arterioscler Thromb Vasc Biol.* 2010;30(2):207–209.

5. Savant S, et al. The Orphan Receptor Tie1 Controls Angiogenesis and Vascular Remodeling by Differentially Regulating Tie2 in Tip and Stalk Cells. *Cell Rep.* 2015;12(11):1761–1773.

6. Shen B, et al. Genetic dissection of tie pathway in mouse lymphatic maturation and valve development.

Arterioscler Thromb Vasc Biol. 2014;34(6):1221–1230.
7. Kapiainen E, et al. The Amino-Terminal Oligomerization Domain of Angiopoietin-2 Affects Vascular Remodeling, Mammary Gland Tumor Growth, and Lung Metastasis in Mice. *Cancer Res.* 2021;81(1):129–143.
8. Sun JF, et al. Microvascular patterning is controlled by fine-tuning the Akt signal. *Proc Natl Acad Sci. U S A.* 2005;102(1):128–133.
9. Holopainen T, et al. Effects of angiopoietin-2-blocking antibody on endothelial cell-cell junctions and lung metastasis. *J Natl Cancer Inst.* 2012;104(6):461–475.
10. Graupera M, et al. Angiogenesis selectively requires the p110alpha isoform of PI3K to control endothelial cell migration. *Nature.* 2008;453(7195):662–666.
11. Eser S, et al. Selective requirement of PI3K/PDK1 signaling for Kras oncogene-driven pancreatic cell plasticity and cancer. *Cancer Cell.* 2013;23(3):406–420.
12. Martinez-Corral I, et al. Vegfr3-CreER (T2) mouse, a new genetic tool for targeting the lymphatic system. *Angiogenesis.* 2016;19(3):433–445.
13. Li Z, et al. Angiopoietin-2 blockade ameliorates autoimmune neuroinflammation by inhibiting leukocyte recruitment into the CNS. *J Clin Invest.* 2020;130(4):1977–1990.
14. Fang S, et al. Critical requirement of VEGF-C in transition to fetal erythropoiesis. *Blood.* 2016;128(5):710–720.
15. Enholm B, et al. Adenoviral expression of vascular endothelial growth factor-C induces lymphangiogenesis in the skin. *Circ Res.* 2001;88(6):623–629.
16. Anisimov A, et al. Activated forms of VEGF-C and VEGF-D provide improved vascular function in skeletal muscle. *Circ Res.* 2009;104(11):1302–1312.
17. Jeltsch M, et al. CCBE1 enhances lymphangiogenesis via a disintegrin and metalloprotease with thrombospondin motifs-3-mediated vascular endothelial growth factor-C activation. *Circulation.* 2014;129(19):1962–1971.
18. Kallio P, et al. Blocking angiopoietin-2 promotes vascular damage and growth inhibition in mouse tumors treated with small doses of radiation. *Cancer Res.* 2020;80(12):2639-2650.
19. Kivivuori S-M, et al. Expression of vascular endothelial growth factor receptor 3 and Tie1 tyrosine kinase receptor on acute leukemia cells. *Pediatr Blood Cancer.* 2007;48(4):387–392.
20. Vaahtomeri K, et al. Locally Triggered Release of the Chemokine CCL21 Promotes Dendritic Cell Transmigration across Lymphatic Endothelia. *Cell Rep.* 2017;19(5):902–909.

21. Persaud K, et al. Involvement of the VEGF receptor 3 in tubular morphogenesis demonstrated with a human anti-human VEGFR-3 monoclonal antibody that antagonizes receptor activation by VEGF-C. *J Cell Sci.* 2004;117(Pt 13):2745–2756.

22. Montoya-Zegarra JA, et al. AutoTube: a novel software for the automated morphometric analysis of vascular networks in tissues. *Angiogenesis.* 2019;22(2):223–236.

23. Vanlandewijck M, et al. A molecular atlas of cell types and zonation in the brain vasculature. *Nature* 2018;554(7693):475–480.

24. Picelli S, et al. Full-length RNA-seq from single cells using Smart-seq2. *Nat Protoc.* 2014;9(1):171–181.

Supplemental Video Legends

Supplemental Video 1. VEGFR3 and Tie1 co-localize upon VEGF-C treatment. Internalization of surface labeled VEGFR3 (red) and Tie1 (green) was monitored live in conditions of 100 ng/ml VEGF-C. Frame frequency is 1/minute. VE-cadherin (blue) was captured every 10th frame. Scale bar is 20 µm.

Supplemental Figure Legends

Supplemental Figure 1. Analysis of Tie-receptor deletion in lymphatic vessels of ear skin. (**A**) Diagram of LEC-specific deletion of Tie-receptors by 4-OHT intragastric injection in control, $Tie1^{i\Delta LEC}$, $Tie2^{i\Delta LEC}$ and $Tie1\ Tie2^{i\Delta LEC}$ pups and analyses at P21. (**B**) Diagram showing Ang2 inactivation in wild-type pups by s.c. or i.p. administration of Ang2 Ab every third day starting at P1 and termination at P21. (**C-D**) Representative images of ear skin immunostained for VE-cadherin, LYVE1 and Tie1 (**C**) or Tie2 (**D**) in control and Tie1-deleted (**C**) and control and Tie2-deleted (**D**) pups at P21. Dashed lines indicate lymphatic capillaries. Scale bars, 50 µm. (**E-F**) Representative images of control, Tie1-deleted (**E**) and Tie2-deleted (**F**) P21 ear skin immunostained for CD31, Prox1 and Tie1 (**E**) or Tie2 (**F**). Dashed lines indicate collecting lymphatic vessels. Scale bars, 50 µm. (**G-H**) qRT-PCR from isolated LECs (**G**) and BECs (**H**) from ear skins of control and Tie1/-2-deleted adult mice. Mean ± SEM, 2-tailed Student's t-test. ***P<0.001.

Supplemental Figure 2. Abnormal collecting lymphatic vessels in the ear skin of Tie1/-2-deleted mice. (**A**) Quantification of widest and narrowest vessel diameters measured separately from proximal region (R1) or distal region (R2) of the collecting lymphatic vessel, and the difference between the two (n numbers are indicated in Figure 2 legend). (**B**) Podoplanin and αSMA staining showing collecting lymphatic vessels in ear skin of Tie1/-2-deleted P21 pups. Arrows point to occasional smooth muscle cells attached to the abnormally shaped vessels. BV, blood vessel. Scale bar, 100 µm. (**C**) Podoplanin and cleaved caspase3 staining showing apoptotic LECs in the Tie1/-2-deleted pups (Control: n=4; Tie1/-2-deleted: n=5). Scale bars, 20 µm. Mean ± SEM, 2-tailed Student's t-test. *P<0.05, **P<0.01, ***P<0.001.

Supplemental Figure 3. Ang2 regulates Tie1 localization in lymphatic vessels. (**A**) Tie1 and Prox1 immunostaining in lymphatic capillaries and collecting vessels of IgG- and Ang2 Ab-treated pups (n=4 per group). Arrows point to perinuclearly localized Tie1. Scale bars, 20 µm. (**B**) Tie1 immunostaining in lymphatic capillaries and collecting vessels in dorsal ear skin of WT and *Ang2*$^{-/-}$ (n=4 per group) pups at P21. Scale bars, 20 µm. Quantification of Tie1 in lymphatic capillaries and collecting vessels, normalized to control. (**C**) LYVE1 staining in ventral ear skin of WT and *Ang2*$^{-/-}$ (n=3 per group) pups at P21. Scale bar, 200 µm. (**D**) Magnification of the areas outlined by the dashed boxes in (**C**). Scale bar, 200 µm. (**E**) Tie2 and Prox1 immunostaining of collecting lymphatic vessels in ear skin of IgG- and Ang2 Ab-treated (n=4 per group) pups at P21. Quantification of Tie2 in collecting lymphatic vessels, normalized to control. Scale bar, 20 µm. (**F**) Tie2 immunostaining of collecting lymphatic vessels in ear skin of WT and *Ang2*$^{-/-}$ (n=4 per group) pups at P21. Scale bar, 20 µm. Quantification of Tie2 in collecting lymphatic vessels, normalized to control. Mean ± SEM, 2-tailed Student's t-test. **P<0.01.

Supplemental Figure 4. VEGFR3 expression in lymphatic vessels of mice deleted of Tie receptors or Ang2. (**A-B**) VEGFR3 and GOLPH4 stainings in Figure 4A are shown here as individual and merged images from the Tie1-deleted (**A**) and Ang2 Ab-treated (**B**) P21 pups. Scale bars, 10 µm. (**C-D**) VEGFR3 immunostaining in lymphatic capillaries of ear skin in control, Tie1/-2-deleted (control: n=7; Tie1/-2-deleted: n=8) (**C**) and Tie2-deleted (control: n=7; Tie2-deleted: n=6) (**D**) pups at P21, normalized to control. Scale bars, 20 µm. Quantification of total VEGFR3 and its fraction in the Golgi complex normalized to control. (**E**) VEGFR3 and LYVE1 staining of fixed but not permeabilized lymphatic capillaries in ear skin of control (n=4) and Tie2-deleted pups at P21 (n=6). Scale bar, 20 µm.

Quantification of VEGFR3 staining on the LEC surface in lymphatic capillaries, normalized to control. (**F**) VEGFR3 and VE-cadherin immunostaining of lymphatic capillaries and collecting vessels in ear skin of WT and *Ang2*$^{-/-}$ (n=3 per group) pups at P21. Scale bars, 20 µm. (**G**) VEGFR3 and LYVE1 staining of fixed but not permeabilized lymphatic capillaries in the ear skin of WT and *Ang2*$^{-/-}$ (n=4 per group) pups at P21. Scale bar, 20 µm. Quantification of VEGFR3 staining on the cell surface of lymphatic capillaries, normalized to control. Mean ± SEM, 2-tailed Student's t-test. *P<0.05, ***P<0.001.

Supplemental Figure 5. VEGFR3 expression in lymphatic vessels of Ang2-deleted mice. (**A**) Diagram showing the schedule of EC- and LEC-specific deletion of Ang2. (**B**) Analysis of Ang2 expression in ear skin of control and *Ang2*$^{i\Delta EC}$ P21 pups by Western blotting (n=3 per group). (**C-D**) Representative images of lymphatic capillaries in ear skins of control, *Ang2*$^{i\Delta EC}$ (**C**) and *Ang2*$^{i\Delta LEC}$ (**D**) pups at P21, immunostained for LYVE1 and VEGFR3. Scale bars, 200 µm (LYVE1) and 50 µm (VEGFR3).

Supplemental Figure 6. VEGFR3 redistribution after various periods of Ang2 Ab treatment. VEGFR3 immunostaining of lymphatic capillaries in ear skin of pups treated with Ang2 Abs for the indicated periods (P12 - P14, - P15, - P16 or - P21, n=2 per group). Scale bars, 50 µm.

Supplemental Figure 7. VEGFR3 staining on the LEC surface in collecting lymphatic vessels of Tie1-deleted, Tie2-deleted or Ang2 Ab-treated pups. VEGFR3 staining of fixed but not permeabilized collecting lymphatic vessels in ear skin of control (n=8) vs. Tie1-deleted (n=6), control (n=4) vs. Tie2-deleted (n=6) and IgG (n=4) vs. Ang2 Ab-treated (n=4) pups at P21. Quantification of VEGFR3 staining on the cell surface of collecting lymphatic vessels, normalized to control. Scale bars, 20 µm. Mean ± SEM, 2-tailed Student's t-test. **P<0.01.

Supplemental Figure 8. Accumulation of VEGFR3 on the surface of LECs and particularly their cell junctions in the absence of VEGF-C. LECs were incubated overnight with or without VEGF-C, or starved overnight of VEGF-C and stimulated with VEGF-C for 30 min and stained for cell surface VEGFR3. Scale bar, 20 µm.

Supplemental Figure 9. VEGFR3 localization in intracellular vesicles in VEGF-C stimulated LECs. (A) Co-localization of VEGFR3 with EGFP-RAB5C and RAB7 in LECs stimulated with VEGF-C for 30, 120 and 240 minutes. The boxed region is shown as two zoom-in images of 1 optical slice. The upper zoom-in image shows VEGFR3, EGFP-RAB5C and RAB7 fluorescence and the lower only the EGFP-RAB5C and RAB7 fluorescence to highlight the vesicles. The white arrows point to some of the VEGFR3 loaded EGFP-RAB5C positive vesicles and the yellow arrows to the VEGFR3 loaded RAB7-positive vesicles. (**B**) The perinuclear EGFP-RAB5C vesicles are EEA1 positive sorting endosomes. Immunofluorescence images of VEGFR3, EEA1 and DAPI stained and EGFP-RAB5C expressing LECs stimulated with VEGF-C for 30 minutes. Scale bars: 20 µm.

Supplemental Figure 10. Colocalization of VEGFR3 and Tie1 with Rab7-positive intracellular vesicles. Receptor internalization, as described in the supplemental materials for VEGFR3 and Tie1 with fluorescently labeled Abs followed by staining for Rab7 in LECs stimulated with VEGF-C for 1 h. The insets are shown without DAPI. The experiment was performed twice with similar results. Scale bar, 10 µm.

Supplemental Figure 11. Analysis of Tie1+VEGFR3 and Notch1+VEGFR3 proximity. PLA and quantification of PLA spots using Tie1+VEGFR3, Notch1+VEGFR3 and their control Abs in permeabilized LECs. n=5 fields of view. Scale bar, 10 µm. Mean ± SEM, 1-way ANOVA with Bonferroni's post hoc test for multiple comparisons. ***P<0.001.

Supplemental Figure 12. Analysis of different VEGFR3 forms, pERK and soluble Tie1. (**A**) Western blot for VEGFR3 and HSC70 in control and trypsin treated LECs. (**B**) Quantification of the VEGFR3 polypeptides, normalized to control sample. (**C**) Western blots showing VEGFR3, Prox1 and HSC70 in ear lysates of control and Tie2-deleted pups at P21. Quantification of VEGFR3 polypeptides normalized to control. (**D-E**) Western blots showing VEGFR3 (**D**) (HSC70 in Figure 6D), pERK and ERK (**E**) in VEGF-C and Ang2 Ab-treated LECs. (**F**) Quantification of the pERK/ERK ratio (n=4 per group), normalized to VEGF-C-treated samples. (**G**) Soluble Tie1 concentration in the medium of VEGF-C stimulated LECs at the indicated time points (n=3 per group). Mean ± SEM (**B**, **C**, **F**) and ± SD (**G**), 2-tailed Student's t-test (**B**, **C** and **G**) and 1-way ANOVA with Bonferroni's post hoc test for multiple comparisons (**F**). *P<0.05, **P<0.01, ***P<0.001.

Supplemental Figure 13. PI3K inhibition regulates VEGFR3 expression in *Pik3ca*^{H1047R}-driven LMs. LYVE1 and VEGFR3 staining of ears from *Vegfr3CreER*^{T2} *R26-LSL-Pik3ca*^{H1047R} mice treated with 4-OHT at 3 weeks of age, then subjected to a 1.5-week treatment with Alpelisib (BYL719) or vehicle, followed by analysis at 7.5 weeks of age. Scale bar, 200 µm. Quantification of VEGFR3 in the lymphatic vessels of *Vegfr3CreER*^{T2} *R26-LSL-Pik3ca*^{H1047R} mice treated with AAV-Ctrl + vehicle (n=4) and AAV-Ctrl + Alpelisib (n=3), normalized to control. The control samples are the same as in Figure 8, A-C. Mean ± SEM, 2-tailed Student's t-test. *P<0.05.

Supplemental Figure 14. Transcriptomic changes in Tie1- and Tie1/2-deficient LECs. (**A**) Schematic overview of LEC isolation from the ear skin at P28 for single-cell RNA sequencing by SMART-seq2. (**B**) UMAP of the LEC clusters. (**C**) Dot plot showing the cluster defining genes. (**D**) UMAP showing LEC clusters with or without neonatal Tie1 or Tie1/-2 deletion. (**E**) The fraction of cells in each cluster for each genotype.

Supplemental Figure 15. Heatmap showing expression of 10 selected differentially expressed genes in each cluster.

Supplemental Figure 16. Expression of Tie1, Tie2 and Ang2 in Tie1-deleted, Tie1/-2-deleted and Ang2-inhibited LECs. (**A-B**) Violin plots showing the expression of Tie1, Tie2 and Ang2 after Tie1 and Tie1/-2 deletion (**A**) or Ang2-inhibition (**B**). Significant changes are indicated in Supplemental Table 1.

Supplemental Figure 17. Differentially expressed genes in Tie1-deleted, Tie1/-2-deleted and Ang2 Ab-treated LECs. Venn diagram showing the number of up- and downregulated genes between the indicated groups. The threshold was set to 0.25 for avg_log2FC and p_valj_adj < 0.05.

Supplemental Figure 18. Expression of Prox1 and VEGFR3 in Tie1-deleted, Tie1/-2-deleted and Ang2-inhibited LECs. (**A-B**) Violin plots showing the expression of Prox1 and VEGFR3 after Tie1 and Tie1/-2 deletion (**A**) or Ang2-inhibition (**B**).

Supplemental Figure 19. Expression of common LEC marker genes in Tie1-deleted, Tie1/-2-deleted and Ang2-inhibited LECs. (**A-C**) Dot plots showing the expression of common LEC marker genes in Tie1-deleted (**A**), Tie1/-2-deleted (**B**) or Ang2-inhibited (**C**) LECs. Significant changes are indicated in Supplemental Table 1.

Supplemental Figure 20. Downregulated and upregulated cellular components in Tie1/-2-deleted and Ang2 Ab-treated capillary LECs. GO Cellular Component analysis of significantly (adjusted $P < 0.05$) downregulated genes and selected significantly (adjusted $P < 0.05$) upregulated genes after Tie1/-2 deletion and Ang2 Ab treatment in capillary LECs.

Supplemental Figure 21. Differentially expressed genes in Tie1-deleted, Tie1/-2-deleted and Ang2-inhibited LECs. (**A-C**) Dot plots showing the expression of differentially expressed genes in Tie1-deleted (**A**), Tie1/-2-deleted (**B**) or Ang2-inhibited (**C**) LECs. Significant changes are indicated in Supplemental Table 1.

Supplemental Figure 22. Low magnification images of VEGF-C treated ears in adult mice. (**A**) VEGFR3 staining of ear skin in control and Tie1/-2-deleted mice treated with Ad-LacZ or Ad-VEGF-C for 10 days (Ad-LacZ: n=3 ears; Tie1/-2-deleted +Ad-LacZ: n=3 ears; Ad-VEGF-C: n=4 ears; Tie1/-2-deleted+Ad-VEGF-C: n=4 ears). (**B**) LYVE1 staining of ear skin in mice treated for 1.5 weeks with Ad-VEGF-C, Ang2 Ab, or both (n=8 ears per group). Scale bars, 1000 µm.

Supplemental Table 1. Gene lists in Tie1-deleted, Tie1/-2-deleted and Ang2-inhibited pups. The table indicates log2FC and adjusted p values for selected genes shown in dot plots and violin plots from the scRNA-Seq data.

Capillary LEC			Collecting LEC			Valve LEC		
Tie1$^{i\Delta LEC}$ **vs** ***Tie1***$^{fl/fl}$			***Tie1***$^{i\Delta LEC}$ **vs** ***Tie1***$^{fl/fl}$			***Tie1***$^{i\Delta LEC}$ **vs** ***Tie1***$^{fl/fl}$		
**Genes**	**log2FC**	**Adjusted pvalue**	**Genes**	**log2FC**	**Adjusted pvalue**	**Genes**	**log2FC**	**Adjusted pvalue**
Tie1	-1,80122289	9,04E-36	*Tie1*	-2,032955812	4,39E-10	*Tie1*	-2,324775667	9,08E-08
Tek			*Tek*			*Tek*	-0,317520468	1
Angpt2	1,11650078	0,00027718	*Angpt2*	1,009640346	1	*Angpt2*	1,227671606	1
Lyve1			*Lyve1*	0,515446006	1	*Lyve1*	0,533494928	1
Prox1			*Prox1*	0,760635808	1	*Prox1*		
Pdpn	-0,326309062	1	*Pdpn*	-0,281050772	1	*Pdpn*		
Flt4	0,473541728	0,01614139	*Flt4*	0,312913946	1	*Flt4*	1,067002335	1
Kdr			*Kdr*			*Kdr*		
Nrp2			*Nrp2*			*Nrp2*	0,430557051	1
Gata2			*Gata2*	-0,346254076	1	*Gata2*		
Itga9			*Itga9*	0,720961227	1	*Itga9*	0,491703327	1
Nfatc1	0,511850884	0,08253078	*Nfatc1*	0,478786103	1	*Nfatc1*	0,422594824	1
Foxc2			*Foxc2*			*Foxc2*	0,418352646	1
Cldn11	-0,375958711	3,99E-15	*Cldn11*			*Cldn11*	-0,306049329	1
Fn1	0,886021221	9,65E-08	*Fn1*	0,603598607	1	*Fn1*	0,494858512	1
Col4a2	0,509772992	0,03645011	*Col4a2*			*Col4a2*		
Hspg2	0,931239006	2,27E-06	*Hspg2*			*Hspg2*		
Thbs1			*Thbs1*			*Thbs1*		
Dcn			*Dcn*			*Dcn*		
Comp	-0,518668304	2,36E-12	*Comp*	-0,930970864	0,23818642	*Comp*		
Col1a1			*Col1a1*			*Col1a1*		
Col1a2			*Col1a2*			*Col1a2*		
Eln			*Eln*			*Eln*		
Fndc1			*Fndc1*	-0,628911875	0,00614803	*Fndc1*	-0,628911875	0,006148025
Clu	-0,790243982	2,13E-17	*Clu*	-0,967499167	0,16943996	*Clu*		
Bgn	-1,103352271	7,60E-11	*Bgn*			*Bgn*		
Thbd	-0,396624516	1,31E-05	*Thbd*			*Thbd*		
Nsg1			*Nsg1*			*Nsg1*		
Sdc1	-0,485354749	0,00011243	*Sdc1*			*Sdc1*		
Lamb1			*Lamb1*			*Lamb1*		
Dab2			*Dab2*			*Dab2*		
Ccl21a	-0,342757423	0,00669603	*Ccl21a*			*Ccl21a*		
Tgm2			*Tgm2*			*Tgm2*		

**Capillary LEC**			**Collecting LEC**			**Valve LEC**		
Tie1 Tie2 $^{i\Delta LEC}$ **vs** ***Tie1 Tie2*** $^{fl/fl}$			***Tie1 Tie2*** $^{i\Delta LEC}$ **vs** ***Tie1 Tie2*** $^{fl/fl}$			***Tie1 Tie2*** $^{i\Delta LEC}$ **vs** ***Tie1 Tie2*** $^{fl/fl}$		
**Genes**	**log2FC**	**Adjusted pvalue**	**Genes**	**log2FC**	**Adjusted pvalue**	**Genes**	**log2FC**	**Adjusted pvalue**
Tie1	-1,792937952	4,77E-36	*Tie1*	-1,346094384	0,01470523	*Tie1*	-1,974126779	1,34E-09
Tek	-1,401234076	1,64E-36	*Tek*	-1,45020368	0,364607276	*Tek*	-1,742559564	3,13E-12
Angpt2	3,192965075	4,38E-56	*Angpt2*	3,328798521	2,81E-09	*Angpt2*	2,357029175	9,16E-12
Lyve1	0,740390958	1,67E-18	*Lyve1*	3,363382632	7,50E-10	*Lyve1*	2,337971984	7,22E-06
Prox1			*Prox1*	0,667806095	1	*Prox1*	-0,555406925	1
Pdpn	0,366298091	1	*Pdpn*			*Pdpn*	1,288034502	0,504868106
Flt4			*Flt4*	0,497436983	1	*Flt4*	0,607103297	1
Kdr	-0,971143444	3,44E-11	*Kdr*	-1,049493859	1	*Kdr*	-0,561771158	1
Nrp2	-1,372004104	6,31E-19	*Nrp2*	-1,441585655	0,009810272	*Nrp2*	0,280741309	0,009570855
Gata2	-0,782311142	1,31E-11	*Gata2*	-0,842434451	1	*Gata2*	-0,84515366	1
Itga9	-0,597799744	0,000107547	*Itga9*	-0,313968404	1	*Itga9*	-1,369790991	0,000607462
Nfatc1	0,265327103	1	*Nfatc1*			*Nfatc1*	0,358351224	1
Foxc2	-0,960748291	3,65E-18	*Foxc2*	-0,665975272	1	*Foxc2*	-0,689338439	1
Cldn11	-1,044482654	2,20E-55	*Cldn11*	-1,129365601	1,37E-05	*Cldn11*	-2,380300592	6,44E-11
Fn1	1,985304436	7,80E-40	*Fn1*	1,75583849	0,000123294	*Fn1*	0,433409991	1
Col4a2	0,696723029	5,36E-05	*Col4a2*			*Col4a2*		
Hspg2	0,604386925	0,000295169	*Hspg2*			*Hspg2*	1,312837164	0,040892691
Thbs1	2,664792098	9,31E-31	*Thbs1*	2,465802429	0,012532137	*Thbs1*	1,57303303	0,002829822
Dcn	-1,869406547	9,25E-72	*Dcn*	-1,55695066	6,82E-11	*Dcn*	-1,100149563	1,58E-18
Comp	-1,909492186	5,30E-65	*Comp*	-2,749605226	2,48E-08	*Comp*	-1,583820129	4,14E-17
Col1a1	-1,146358771	1,10E-64	*Col1a1*	-0,326059719	6,78E-06	*Col1a1*	-0,436852676	8,95E-17
Col1a2	-1,190926612	1,34E-64	*Col1a2*	-0,620773306	1,64E-07	*Col1a2*	-0,740567823	3,82E-18
Eln	-2,442204	2,44E-52	*Eln*	-1,815127997	0,000440931	*Eln*	-1,096721879	1,65E-10
Fndc1	-2,511690499	4,12E-47	*Fndc1*	-1,919888164	1,78E-05	*Fndc1*	-1,198822351	7,20E-11
Clu	-0,886304224	3,34E-46	*Clu*	-2,025461594	3,26E-07	*Clu*	-1,428158582	6,06E-12
Bgn	-0,814469323	5,22E-31	*Bgn*			*Bgn*		
Thbd	-0,532080338	2,24E-14	*Thbd*	-1,487935721	0,027564267	*Thbd*	-0,87564913	0,003475684
Nsg1	-1,214937196	8,40E-17	*Nsg1*			*Nsg1*	-1,372806243	6,85E-06
Sdc1	-0,848085411	4,04E-11	*Sdc1*			*Sdc1*		
Lamb1	-0,555094514	0,033553947	*Lamb1*			*Lamb1*		
Dab2	-1,384087897	3,20E-24	*Dab2*	-2,028599908	1,56E-05	*Dab2*	-0,993520093	1,98E-06
Ccl21a	-0,97780657	2,28E-22	*Ccl21a*			*Ccl21a*		
Tgm2	-1,320851078	1,23E-15	*Tgm2*	-1,208063533	0,004095226	*Tgm2*		

Capillary LEC			Collecting LEC			Valve LEC		
Ang2 Ab vs IgG			Ang2 Ab vs IgG			Ang2 Ab vs IgG		
**Genes**	**log2FC**	**Adjusted pvalue**	**Genes**	**log2FC**	**Adjusted pvalue**	**Genes**	**log2FC**	**Adjusted pvalue**
Tie1			*Tie1*			*Tie1*		
Tek	-0,242674673	1	*Tek*			*Tek*	-0,206637773	1
Angpt2	2,197228352	2,57E-64	*Angpt2*	1,710539294	3,62E-15	*Angpt2*	1,345064838	3,42E-09
Lyve1	0,654937773	2,65E-19	*Lyve1*	0,789449756	1	*Lyve1*	0,19714307	1
Prox1			*Prox1*	0,330930989	1	*Prox1*		
Pdpn	-0,290045501	0,000265906	*Pdpn*			*Pdpn*	0,195686951	1
Flt4			*Flt4*	-0,313278713	1	*Flt4*	-0,183338073	1
Kdr	-0,250975275	0,002940412	*Kdr*	-0,250670578	1	*Kdr*	-0,255125884	1
Nrp2	-0,653007916	2,08E-11	*Nrp2*	-0,625268098	0,034089986	*Nrp2*	-0,165798648	1
Gata2	-0,256899643	1	*Gata2*			*Gata2*	-0,240446284	1
Itga9	-0,356790297	0,054616654	*Itga9*			*Itga9*		
Nfatc1	0,151658058	1	*Nfatc1*	0,395734248	1	*Nfatc1*		
Foxc2	-0,284060708	0,013438187	*Foxc2*			*Foxc2*		
Cldn11			*Cldn11*			*Cldn11*	-0,246953397	1
Fn1	1,22281961	7,15E-32	*Fn1*	0,423270328	1	*Fn1*	-0,19274462	1
Col4a2			*Col4a2*			*Col4a2*		
Hspg2	0,333035527	1,59E-05	*Hspg2*			*Hspg2*		
Thbs1	1,188221989	4,29E-20	*Thbs1*	1,180028003	0,006471399	*Thbs1*		
Comp	-0,299209988	0,000676886	*Comp*			*Comp*		
Col1a1			*Col1a1*			*Col1a1*		
Col1a2			*Col1a2*			*Col1a2*		
Eln	-0,984066249	5,17E-42	*Eln*	-0,66498831	5,79E-05	*Eln*	-0,646928486	0,00031905
Fndc1	-0,608950114	3,76E-09	*Fndc1*	-0,830459042	7,14E-05	*Fndc1*		
Clu			*Clu*			*Clu*		
Bgn	-0,381372972	0,00018957	*Bgn*			*Bgn*		
Thbd	-0,366933584	0,18240096	*Thbd*			*Thbd*		
Nsg1	-0,726818155	6,11E-20	*Nsg1*			*Nsg1*		
Sdc1	-0,355539823	4,49E-06	*Sdc1*			*Sdc1*		
Lamb1			*Lamb1*			*Lamb1*		
Dab2	-0,488204887	6,23E-07	*Dab2*			*Dab2*		
Ccl21a	-0,837277104	8,36E-19	*Ccl21a*			*Ccl21a*		
Tgm2	-0,582820743	5,31E-15	*Tgm2*			*Tgm2*		

Supplemental Figure 1

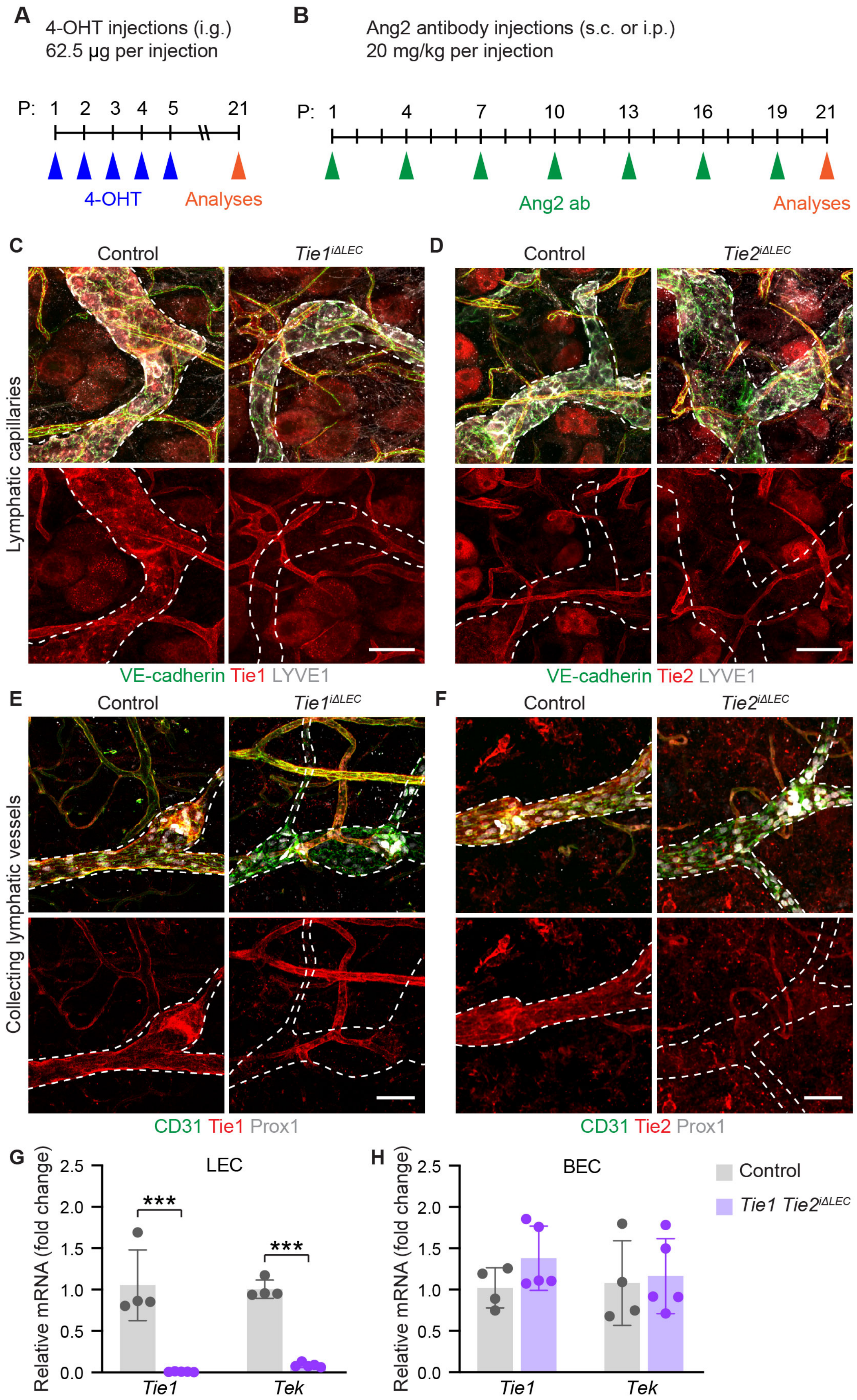

Supplemental Figure 1. Analysis of Tie-receptor deletion in lymphatic vessels of ear skin. (**A**) Diagram of LEC-specific deletion of Tie-receptors by 4-OHT intragastric injection in control, *Tie1*$^{i\Delta LEC}$, *Tie2*$^{i\Delta LEC}$ and *Tie1 Tie2*$^{i\Delta LEC}$ pups and analyses at P21. (**B**) Diagram showing Ang2 inactivation in wild-type pups by s.c. or i.p. administration of Ang2 Ab every third day starting at P1 and termination at P21. (**C-D**) Representative images of ear skin immunostained for VE-cadherin, LYVE1 and Tie1 (**C**) or Tie2 (**D**) in control and Tie1-deleted (**C**) and control and Tie2-deleted (**D**) pups at P21. Dashed lines indicate lymphatic capillaries. Scale bars, 50 µm. (**E-F**) Representative images of control, Tie1-deleted (**E**) and Tie2-deleted (**F**) P21 ear skin immunostained for CD31, Prox1 and Tie1 (**E**) or Tie2 (**F**). Dashed lines indicate collecting lymphatic vessels. Scale bars, 50 µm. (**G-H**) qRT-PCR from isolated LECs (**G**) and BECs (**H**) from ear skins of control and Tie1/-2-deleted adult mice. Mean ± SEM, 2-tailed Student's t-test. ***P<0.001.

Supplemental Figure 2

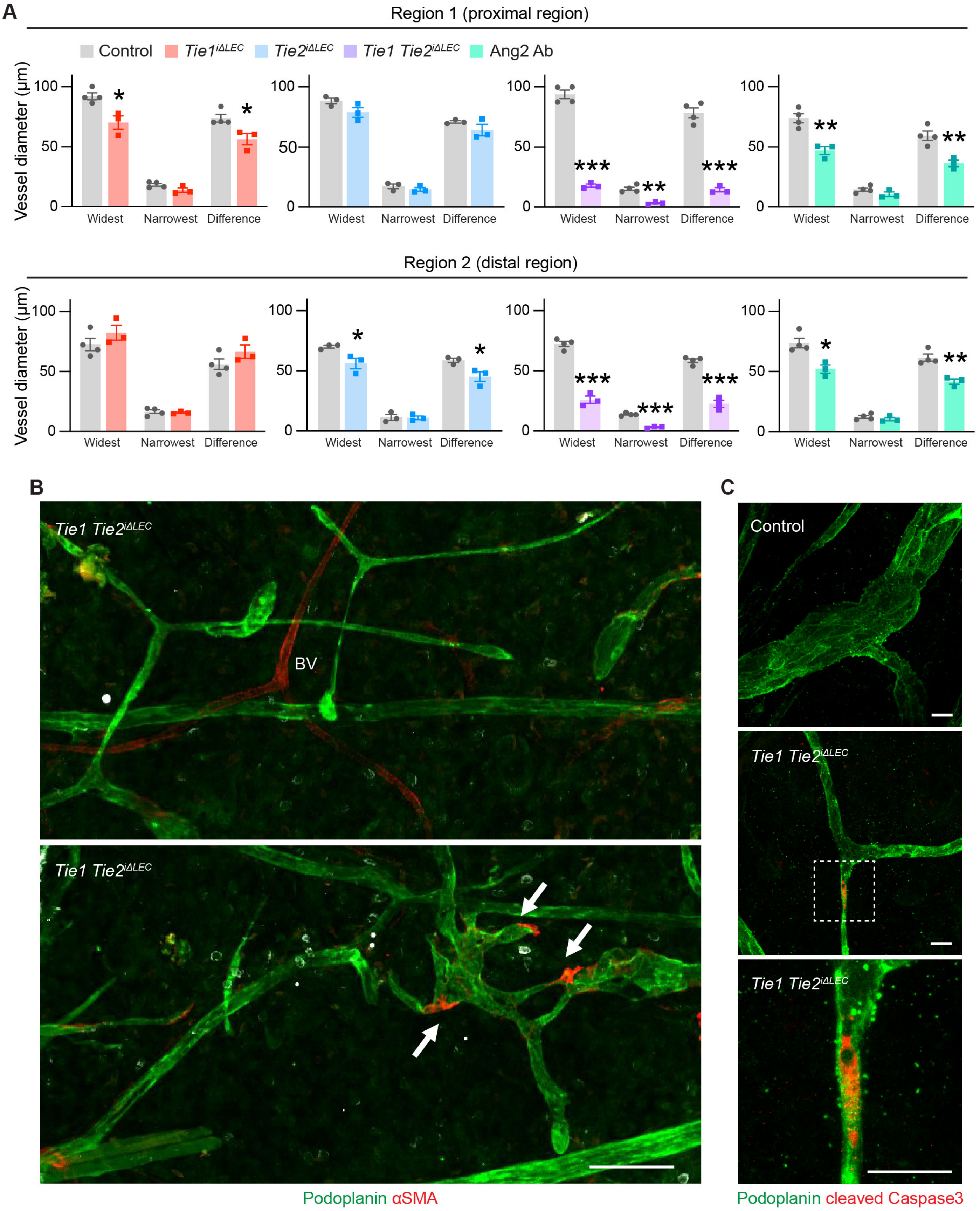

Supplemental Figure 2. Abnormal collecting lymphatic vessels in the ear skin of Tie1/-2-deleted mice. (**A**) Quantification of widest and narrowest vessel diameters measured separately from proximal region (R1) or distal region (R2) of the collecting lymphatic vessel, and the difference between the two (n numbers are indicated in Figure 2 legend). (**B**) Podoplanin and αSMA staining showing collecting lymphatic vessels in ear skin of Tie1/-2-deleted P21 pups. Arrows point to occasional smooth muscle cells attached to the abnormally shaped vessels. BV, blood vessel. Scale bar, 100 μm. (**C**) Podoplanin and cleaved caspase3 staining showing apoptotic LECs in the Tie1/-2-deleted pups (Control: n=4; Tie1/-2-deleted: n=5). Scale bars, 20 μm. Mean ± SEM, 2-tailed Student's t-test. *P<0.05, **P<0.01, ***P<0.001.

Supplemental Figure 3

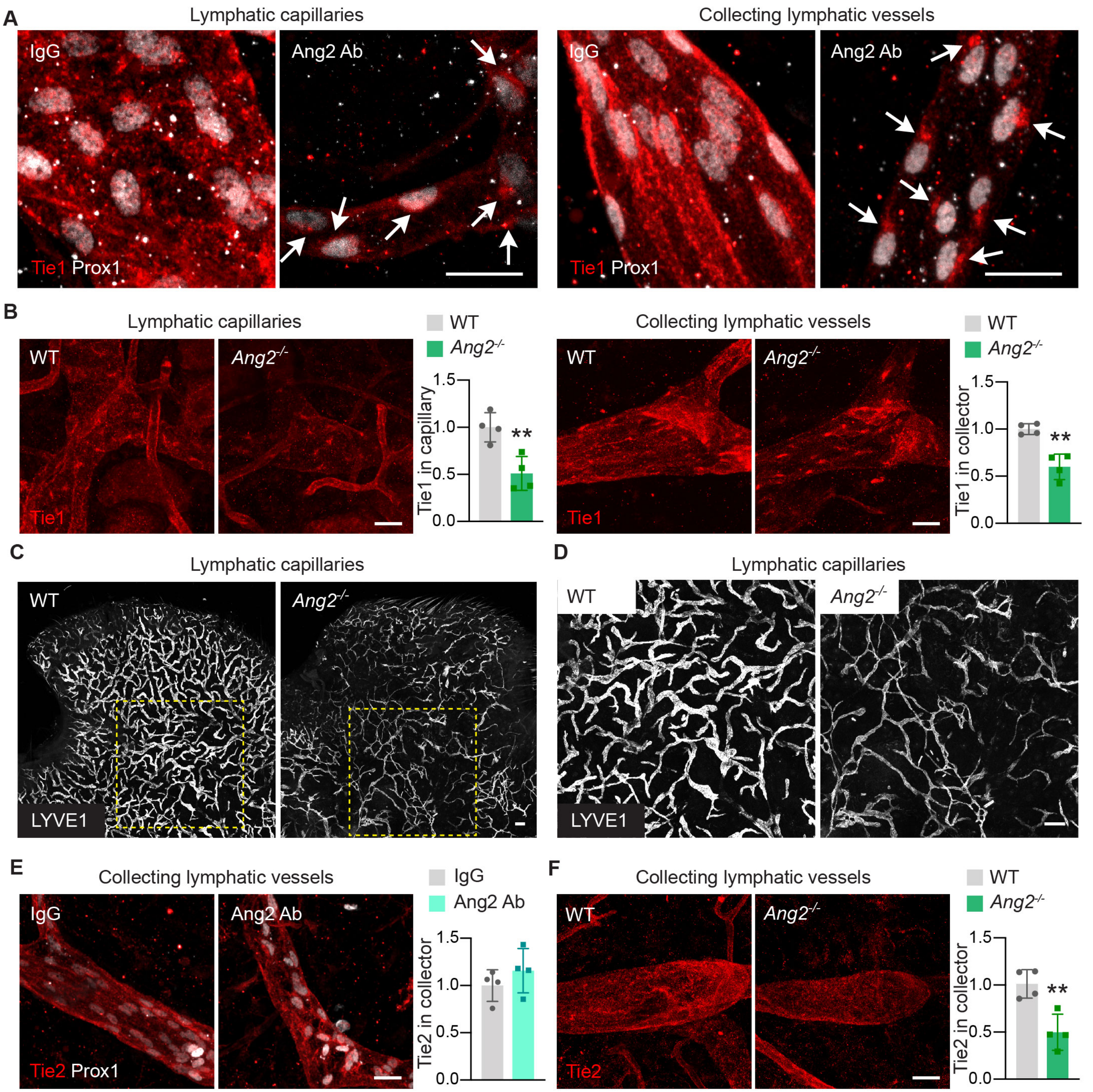

Supplemental Figure 3. Ang2 regulates Tie1 localization in lymphatic vessels. (**A**) Tie1 and Prox1 immunostaining in lymphatic capillaries and collecting vessels of IgG- and Ang2 Ab-treated pups (n=4 per group). Arrows point to perinuclearly localized Tie1. Scale bars, 20 µm. (**B**) Tie1 immunostaining in lymphatic capillaries and collecting vessels in dorsal ear skin of WT and *Ang2*$^{-/-}$ (n=4 per group) pups at P21. Scale bars, 20 µm. Quantification of Tie1 in lymphatic capillaries and collecting vessels, normalized to control. (**C**) LYVE1 staining in ventral ear skin of WT and *Ang2*$^{-/-}$ (n=3 per group) pups at P21. Scale bar, 200 µm. (**D**) Magnification of the areas outlined by the dashed boxes in (**C**). Scale bar, 200 µm. (**E**) Tie2 and Prox1 immunostaining of collecting lymphatic vessels in ear skin of IgG- and Ang2 Ab-treated (n=4 per group) pups at P21. Quantification of Tie2 in collecting lymphatic vessels, normalized to control. Scale bar, 20 µm. (**F**) Tie2 immunostaining of collecting lymphatic vessels in ear skin of WT and *Ang2*$^{-/-}$ (n=4 per group) pups at P21. Scale bar, 20 µm. Quantification of Tie2 in collecting lymphatic vessels, normalized to control. Mean ± SEM, 2-tailed Student's t-test. **P<0.01.

Supplemental Figure 4

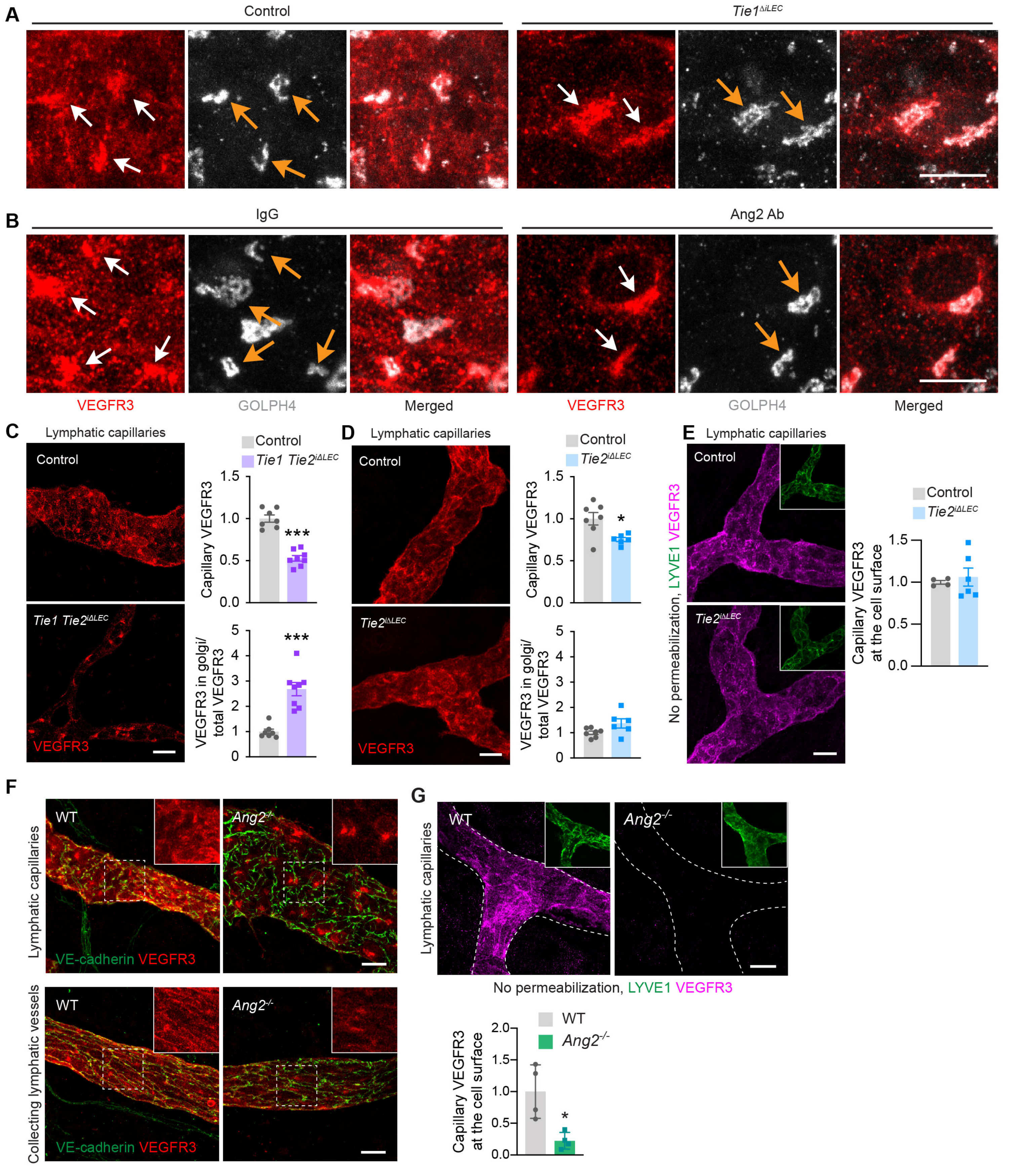

Supplemental Figure 4. VEGFR3 expression in lymphatic vessels of mice deleted of Tie receptors or Ang2. (**A-B**) VEGFR3 and GOLPH4 stainings in Figure 4A are shown here as individual and merged images from the Tie1-deleted (**A**) and Ang2 Ab-treated (**B**) P21 pups. Scale bars, 10 µm. (**C-D**) VEGFR3 immunostaining in lymphatic capillaries of ear skin in control, Tie1/-2-deleted (control: n=7; Tie1/-2-deleted: n=8) (**C**) and Tie2-deleted (control: n=7; Tie2-deleted: n=6) (**D**) pups at P21. Scale bars, 20 µm. Quantification of total VEGFR3 and its fraction in the Golgi complex normalized to control. (**E**) VEGFR3 and LYVE1 staining of fixed but not permeabilized lymphatic capillaries in ear skin of control (n=4) and Tie2-deleted pups at P21 (n=6). Scale bar, 20 µm. Quantification of VEGFR3 staining on the LEC surface in lymphatic capillaries, normalized to control. (**F**) VEGFR3 and VE-cadherin immunostaining of lymphatic capillaries and collecting vessels in ear skin of WT and *Ang2*$^{-/-}$ (n=3 per group) pups at P21. Scale bars, 20 µm. (**G**) VEGFR3 and LYVE1 staining of fixed but not permeabilized lymphatic capillaries in the ear skin of WT and *Ang2*$^{-/-}$ (n=4 per group) pups at P21. Scale bar, 20 µm. Quantification of VEGFR3 staining on the cell surface of lymphatic capillaries, normalized to control. Mean ± SEM, 2-tailed Student's t-test. *P<0.05, ***P<0.001.

Supplemental Figure 5

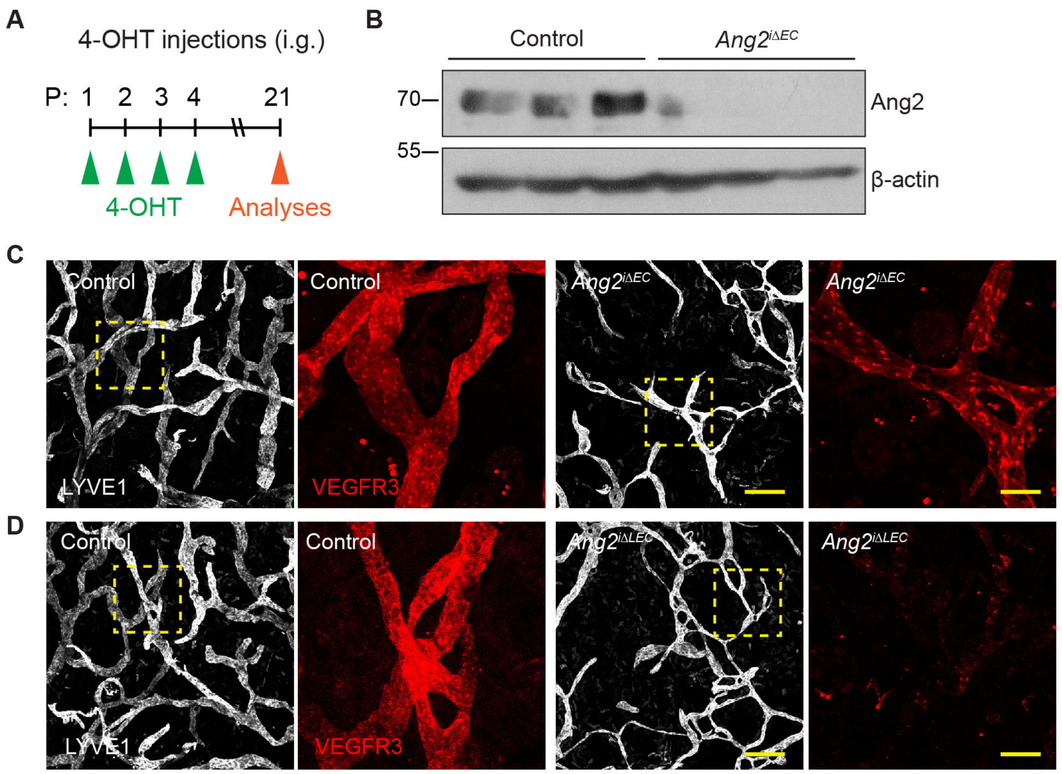

Supplemental Figure 5. VEGFR3 expression in lymphatic vessels of Ang2-deleted mice. (**A**) Diagram showing the schedule of EC- and LEC-specific deletion of Ang2. (**B**) Analysis of Ang2 expression in ear skin of control and *Ang2*$^{i\Delta EC}$ P21 pups by Western blotting (n=3 per group). (**C-D**) Representative images of lymphatic capillaries in ear skins of control, *Ang2*$^{i\Delta EC}$ (**C**) and *Ang2*$^{i\Delta LEC}$ (**D**) pups at P21, immunostained for LYVE1 and VEGFR3. Scale bars, 200 μm (LYVE1) and 50 μm (VEGFR3).

Supplemental Figure 6

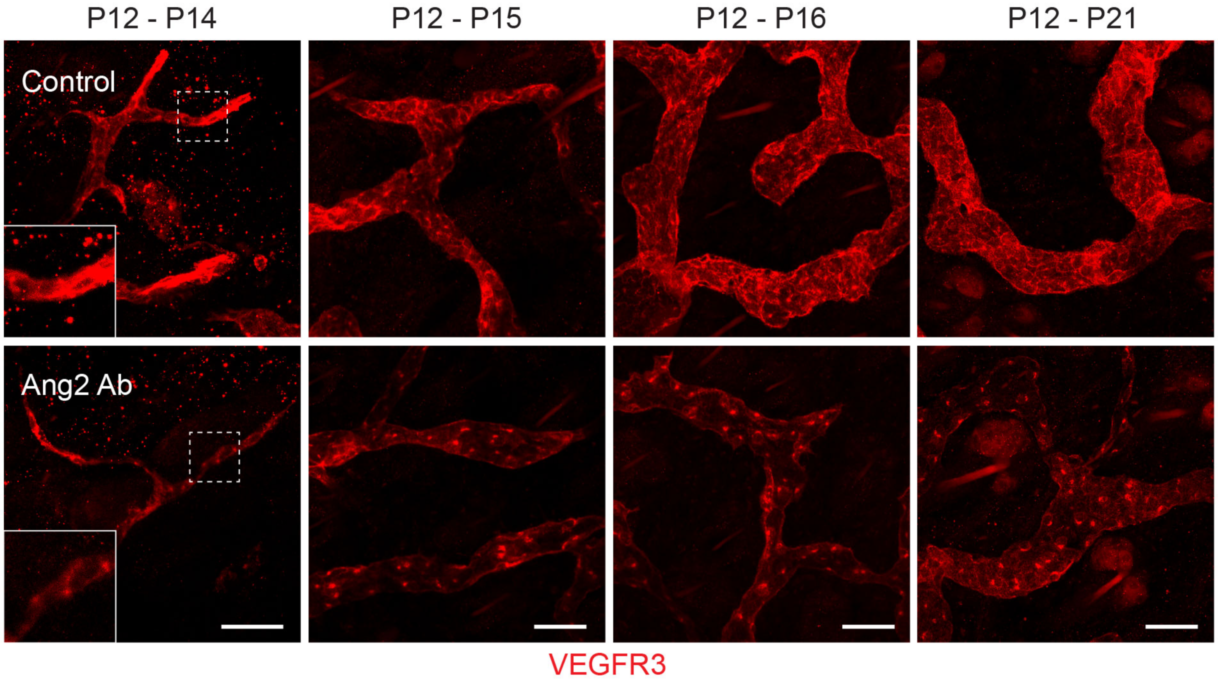

Supplemental Figure 6. VEGFR3 redistribution after various periods of Ang2 Ab treatment. VEGFR3 immunostaining of lymphatic capillaries in ear skin of pups treated with Ang2 Abs for the indicated periods (P12 - P14, - P15, - P16 or - P21, n=2 per group). Scale bars, 50 µm.

Supplemental Figure 7

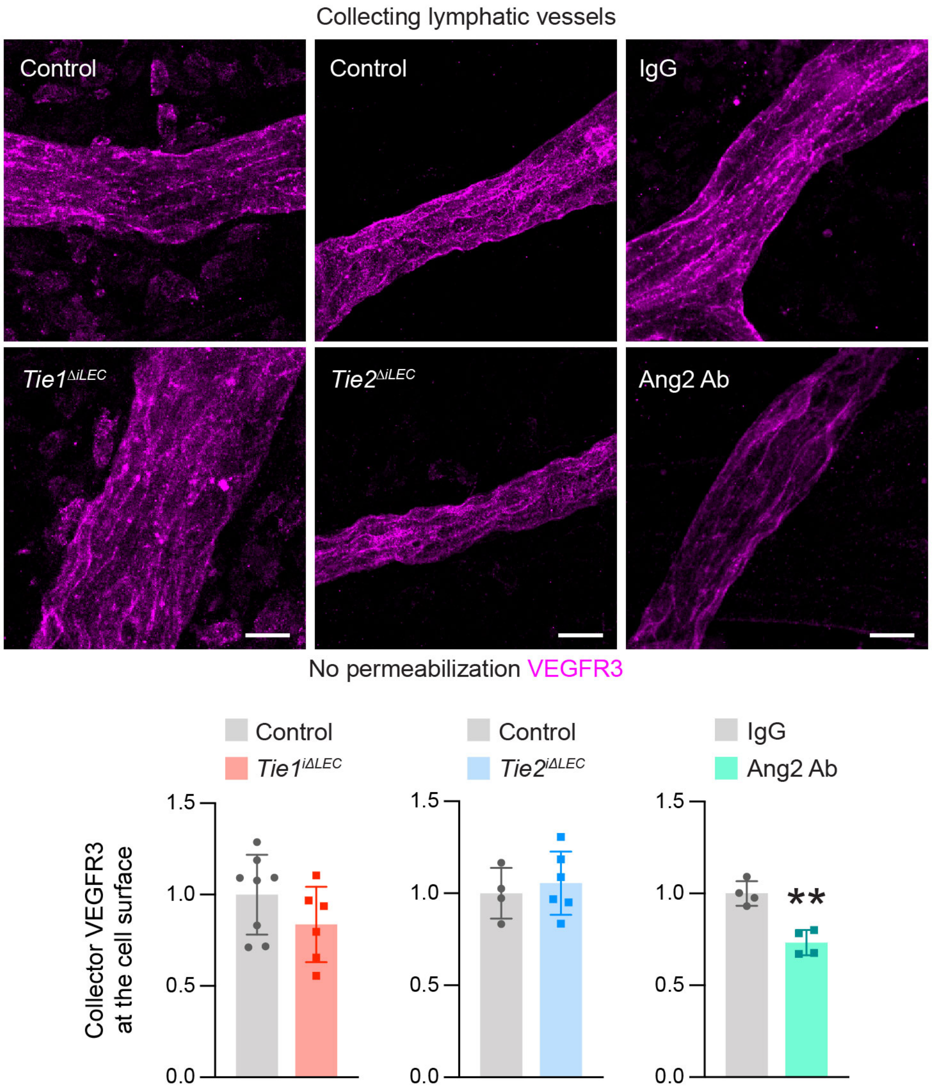

Supplemental Figure 7. VEGFR3 staining on the LEC surface in collecting lymphatic vessels of Tie1-deleted, Tie2-deleted or Ang2 Ab-treated pups. VEGFR3 staining of fixed but not permeabilized collecting lymphatic vessels in ear skin of control (n=8) vs. Tie1-deleted (n=6), control (n=4) vs. Tie2-deleted (n=6) and IgG (n=4) vs. Ang2 Ab-treated (n=4) pups at P21. Quantification of VEGFR3 staining on the cell surface of collecting lymphatic vessels, normalized to control. Scale bars, 20 μm. Mean ± SEM, 2-tailed Student's t-test. **P<0.01.

Supplemental Figure 8

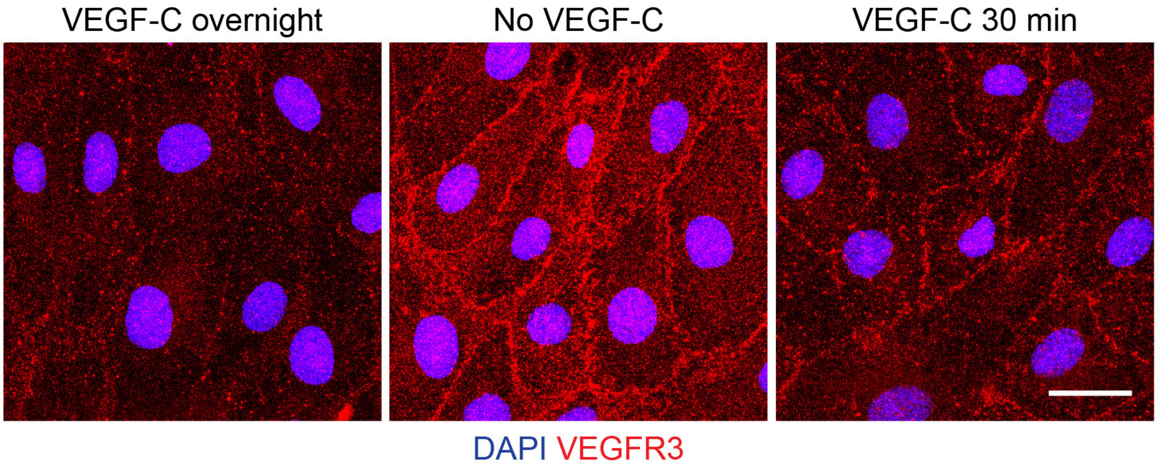

Supplemental Figure 8. Accumulation of VEGFR3 on the surface of LECs and particularly their cell junctions in the absence of VEGF-C. LECs were incubated overnight with or without VEGF-C, or starved overnight of VEGF-C and stimulated with VEGF-C for 30 min and stained for cell surface VEGFR3. Scale bar, 20 μm.

Supplemental Figure 9

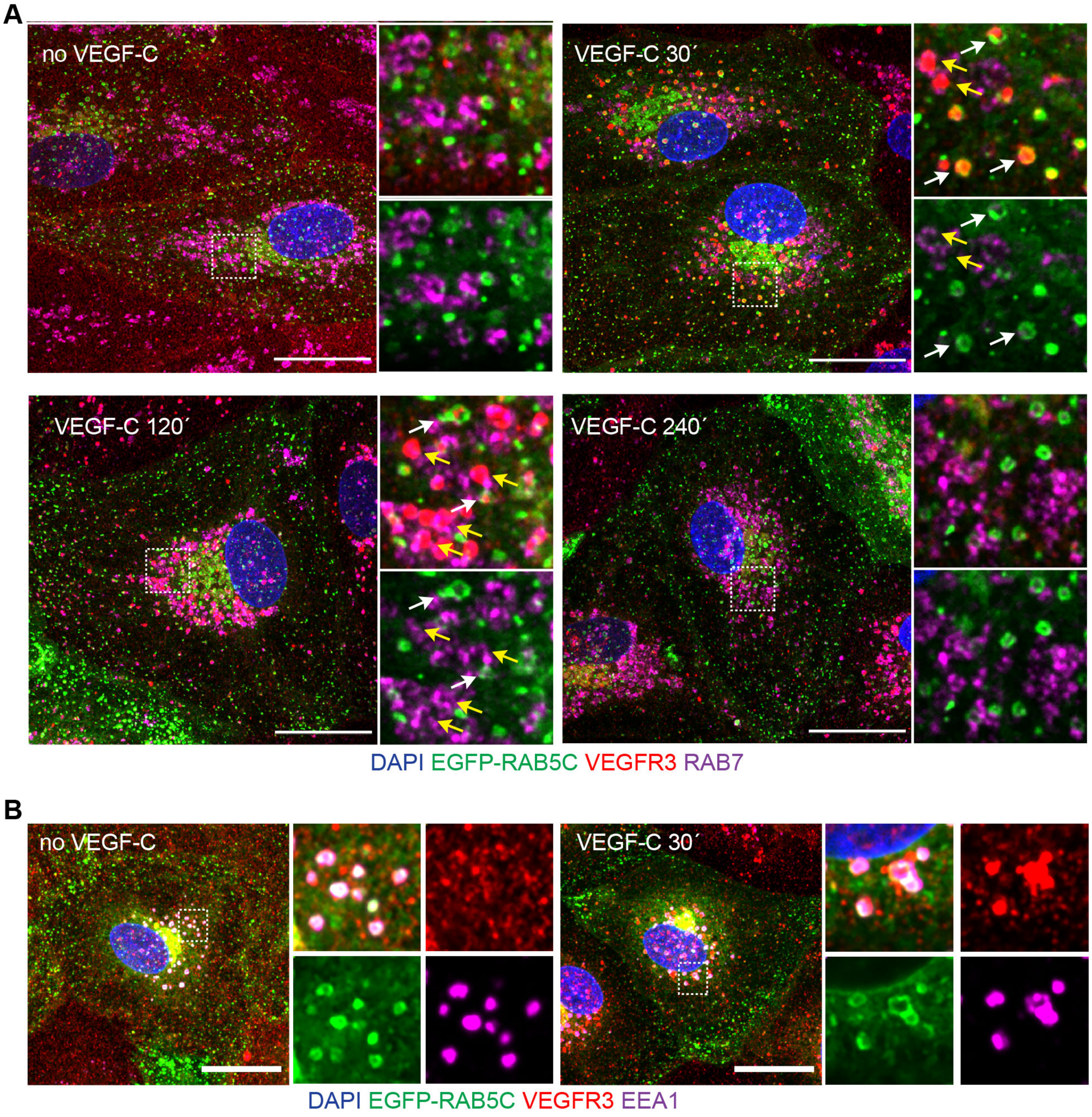

Supplemental Figure 9. VEGFR3 localization in intracellular vesicles in VEGF-C stimulated LECs. (**A**) Co-localization of VEGFR3 with EGFP-RAB5C and RAB7 in LECs stimulated with VEGF-C for 30, 120 and 240 minutes. The boxed region is shown as two zoom-in images of 1 optical slice. The upper zoom-in image shows VEGFR3, EGFP-RAB5C and RAB7 fluorescence and the lower only the EGFP-RAB5C and RAB7 fluorescence to highlight the vesicles. The white arrows point to some of the VEGFR3 loaded EGFP-RAB5C positive vesicles and the yellow arrows to the VEGFR3 loaded RAB7-positive vesicles. (**B**) The perinuclear EGFP-RAB5C vesicles are EEA1 positive sorting endosomes. Immunofluorescence images of VEGFR3, EEA1 and DAPI stained and EGFP-RAB5C expressing LECs stimulated with VEGF-C for 30 minutes. Scale bars: 20 µm.

Supplemental Figure 10

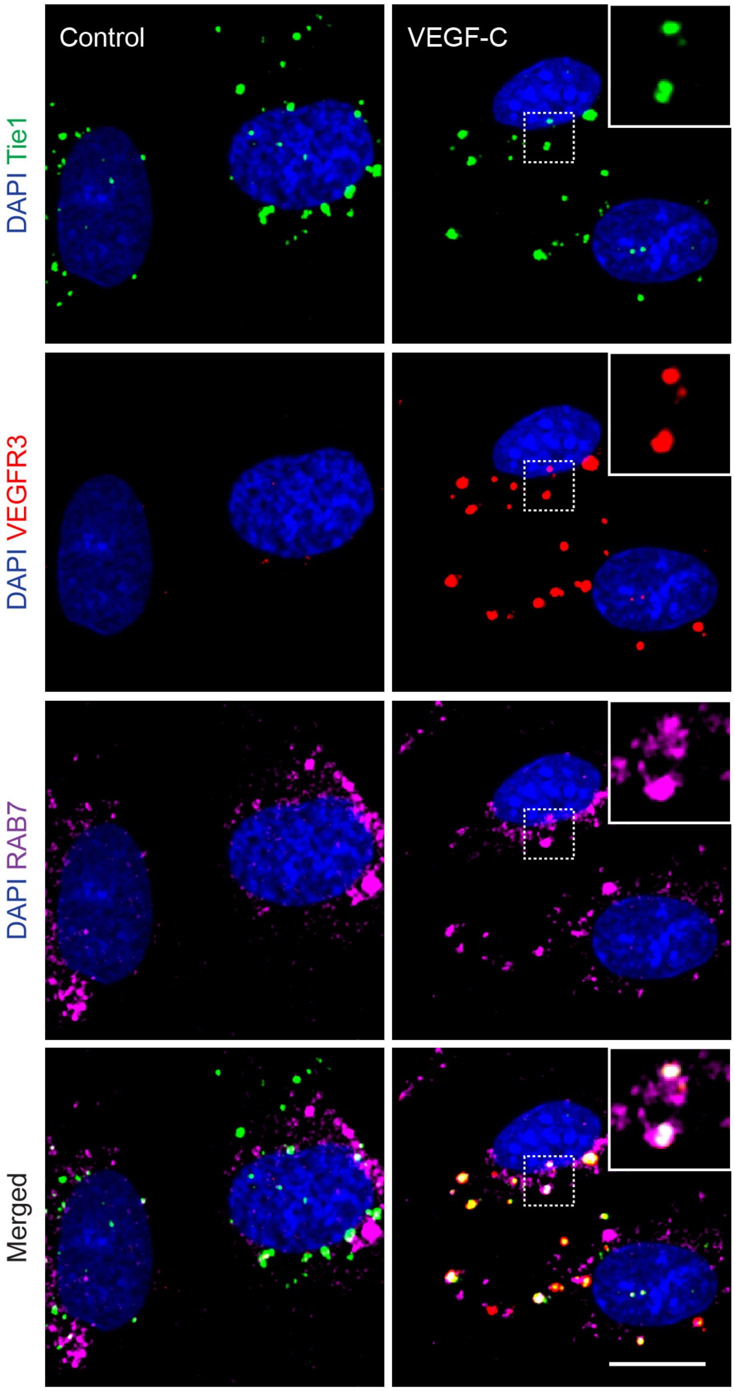

Supplemental Figure 10. Colocalization of VEGFR3 and Tie1 with Rab7-positive intracellular vesicles. Receptor internalization, as described in the supplemental materials for VEGFR3 and Tie1 with fluorescently labeled Abs followed by staining for Rab7 in LECs stimulated with VEGF-C for 1 h. The insets are shown without DAPI. The experiment was performed twice with similar results. Scale bar, 10 μm.

Supplemental Figure 11

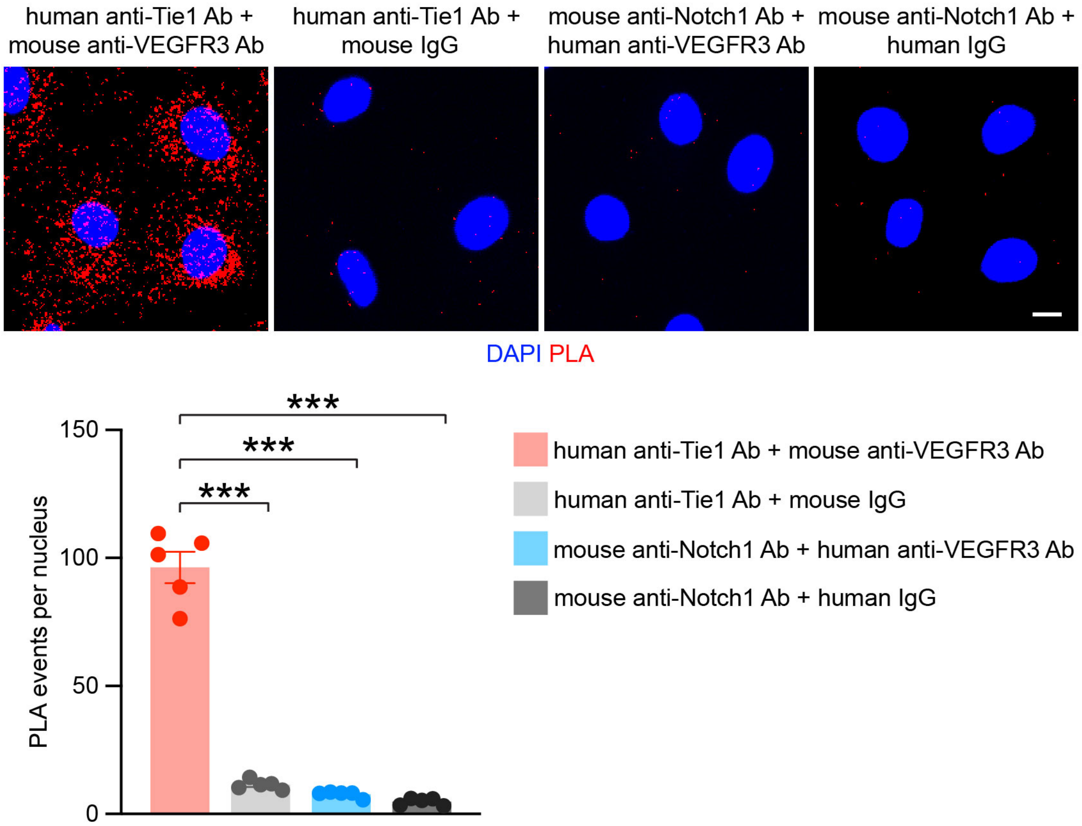

Supplemental Figure 11. Analysis of Tie1+VEGFR3 and Notch1+VEGFR3 proximity. PLA and quantification of PLA spots using Tie1+VEGFR3, Notch1+VEGFR3 and their control Abs in permeabilized LECs. n=5 fields of view. Scale bar, 10 µm. Mean ± SEM, 1-way ANOVA with Bonferroni's post hoc test for multiple comparisons. ***P<0.001.

Supplemental Figure 12

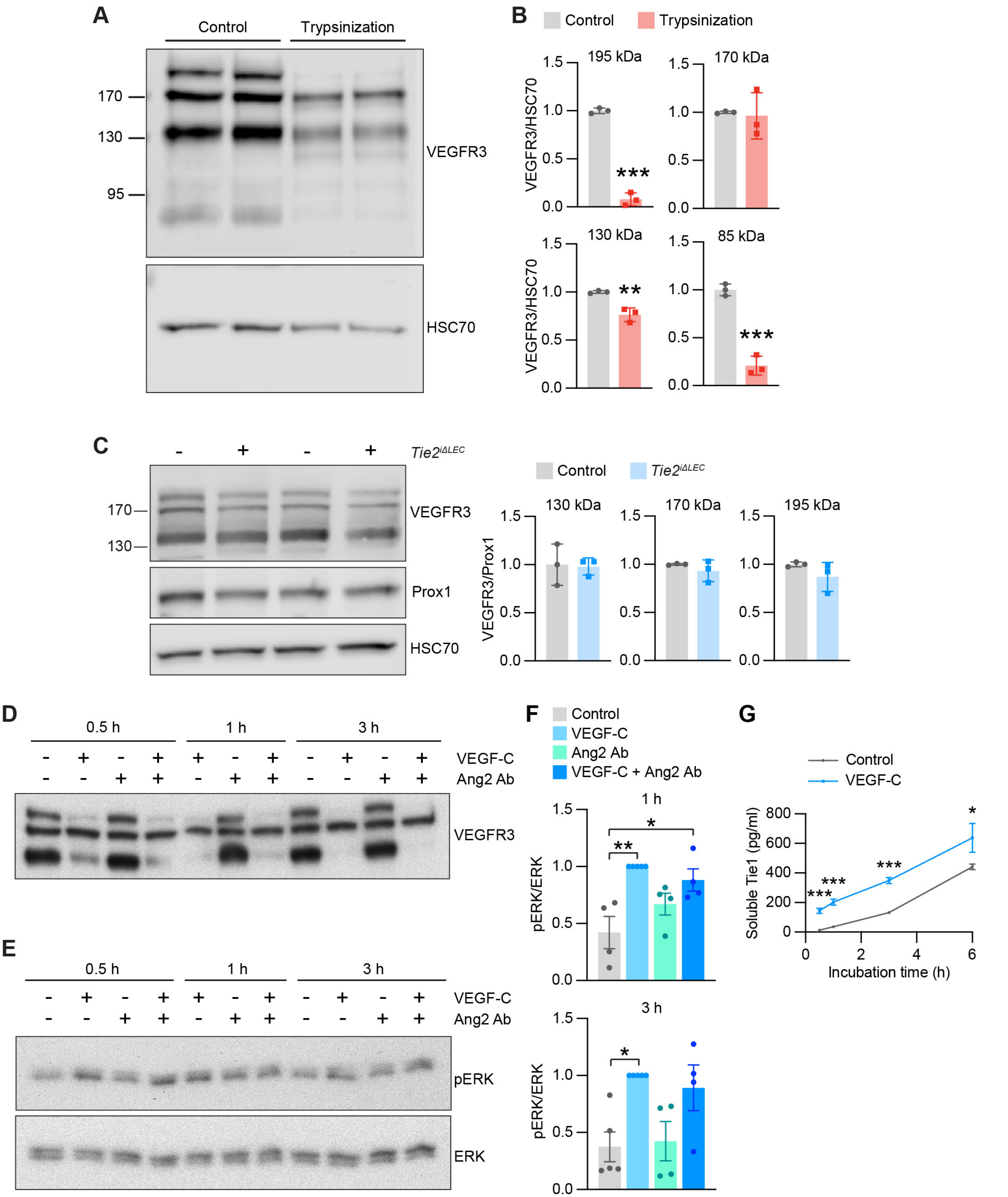

Supplemental Figure 12. Analysis of different VEGFR3 forms, pERK and soluble Tie1. (**A**) Western blot for VEGFR3 and HSC70 in control and trypsin treated LECs. (**B**) Quantification of the VEGFR3 polypeptides, normalized to control sample. (**C**) Western blots showing VEGFR3, Prox1 and HSC70 in ear lysates of control and Tie2-deleted pups at P21. Quantification of VEGFR3 polypeptides normalized to control. (**D-E**) Western blots showing VEGFR3 (**D**) (HSC70 in Figure 6D), pERK and ERK (**E**) in VEGF-C and Ang2 Ab-treated LECs. (**F**) Quantification of the pERK/ERK ratio (n=4 per group), normalized to VEGF-C-treated samples. (**G**) Soluble Tie1 concentration in the medium of VEGF-C stimulated LECs at the indicated time points (n=3 per group). Mean ± SEM (**B**, **C**, **F**) and ± SD (**G**), 2-tailed Student's t-test (**B**, **C** and **G**) and 1-way ANOVA with Bonferroni's post hoc test for multiple comparisons (**F**). *P<0.05, **P<0.01, ***P<0.001.

Supplemental Figure 13

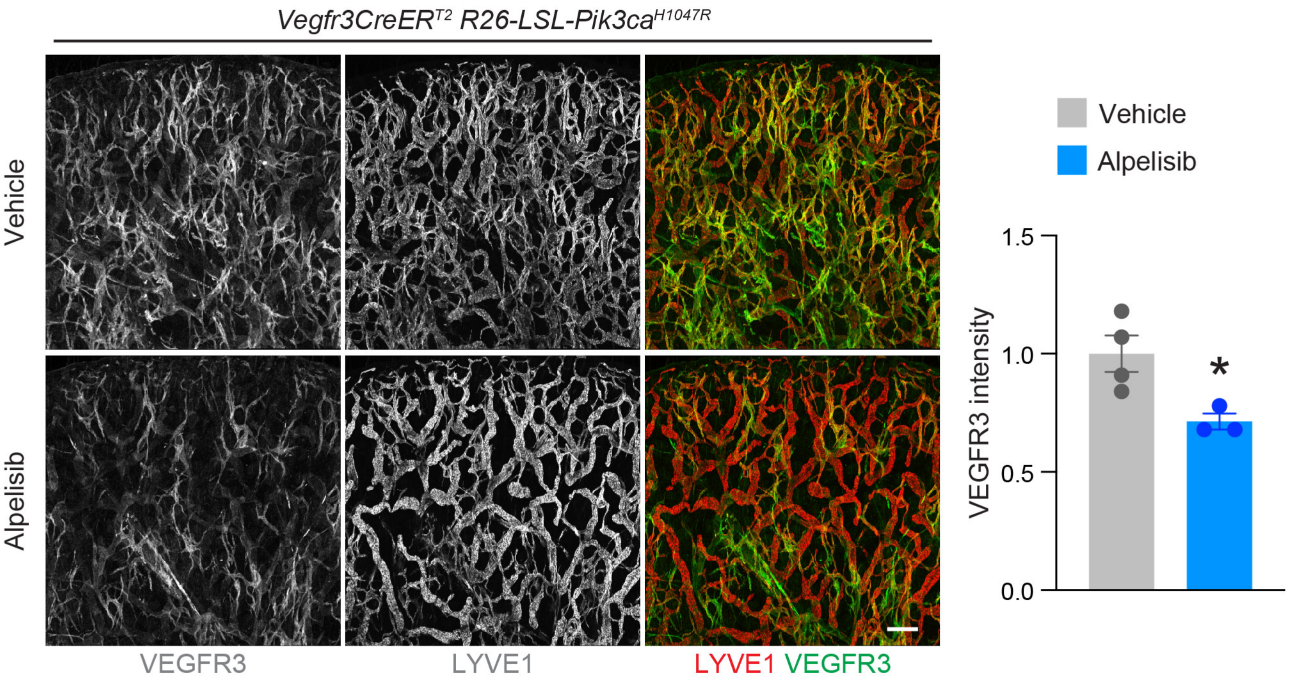

Supplemental Figure 13. PI3K inhibition regulates VEGFR3 expression in *Pik3ca^{H1047R}*-driven LMs. LYVE1 and VEGFR3 staining of ears from *Vegfr3CreER^{T2} R26-LSL-Pik3ca^{H1047R}* mice treated with 4-OHT at 3 weeks of age, then subjected to a 1.5-week treatment with Alpelisib (BYL719) or vehicle, followed by analysis at 7.5 weeks of age. Scale bar, 200 µm. Quantification of VEGFR3 in the lymphatic vessels of *Vegfr3CreER^{T2} R26-LSL-Pik3ca^{H1047R}* mice treated with AAV-Ctrl + vehicle (n=4) and AAV-Ctrl + Alpelisib (n=3), normalized to control. The control samples are the same as in Figure 8, A-C. Mean ± SEM, 2-tailed Student's t-test. *P<0.05.

Supplemental Figure 14

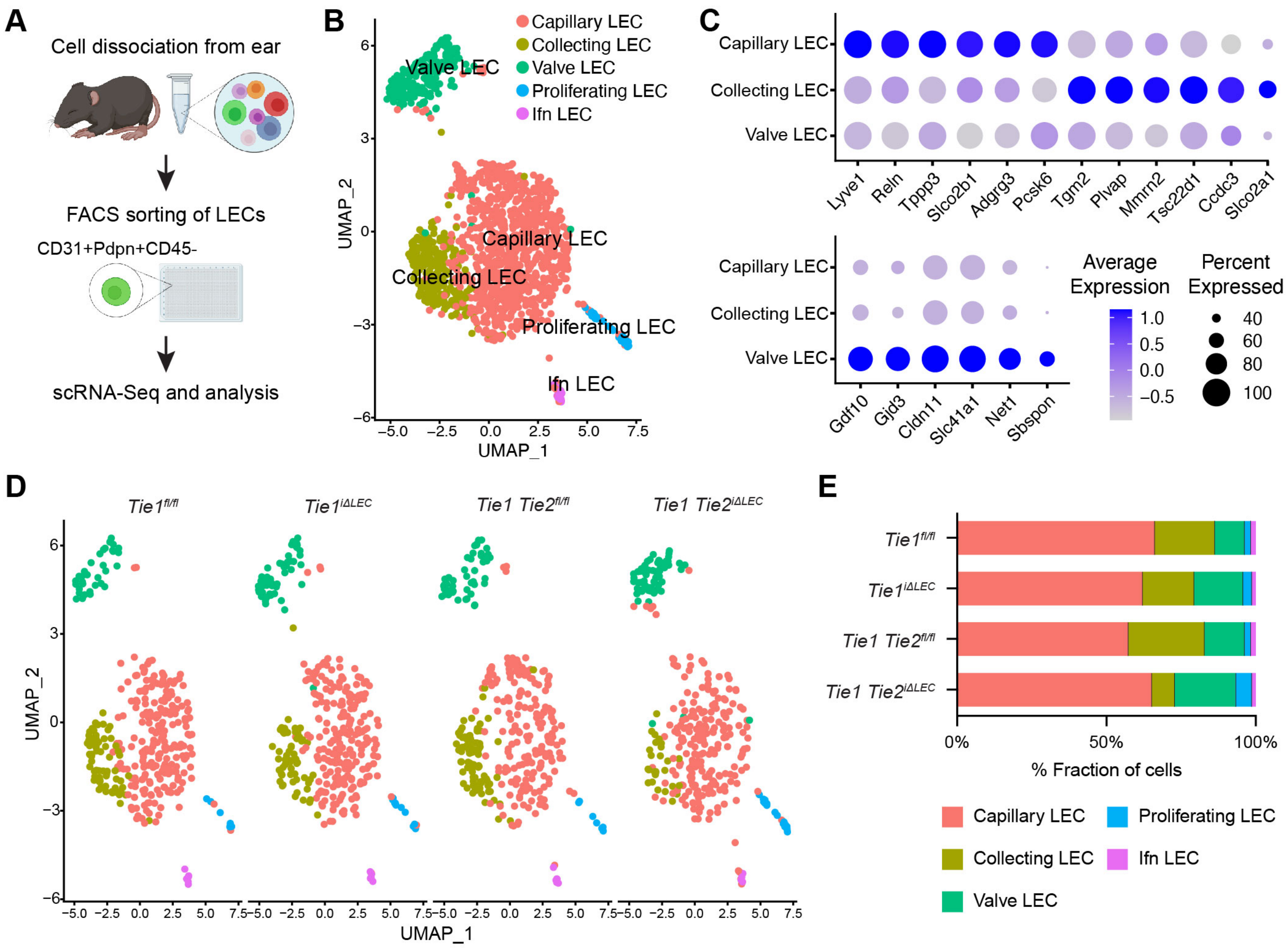

Supplemental Figure 14. Transcriptomic changes in Tie1- and Tie1/-2-deficient LECs. (**A**) Schematic overview of LEC isolation from the ear skin at P28 for single-cell RNA sequencing by SMART-seq2. (**B**) UMAP of the LEC clusters. (**C**) Dot plot showing the cluster defining genes. (**D**) UMAP showing LEC clusters with or without neonatal Tie1 or Tie1/-2 deletion. (**E**) The fraction of cells in each cluster for each genotype.

Supplemental Figure 15

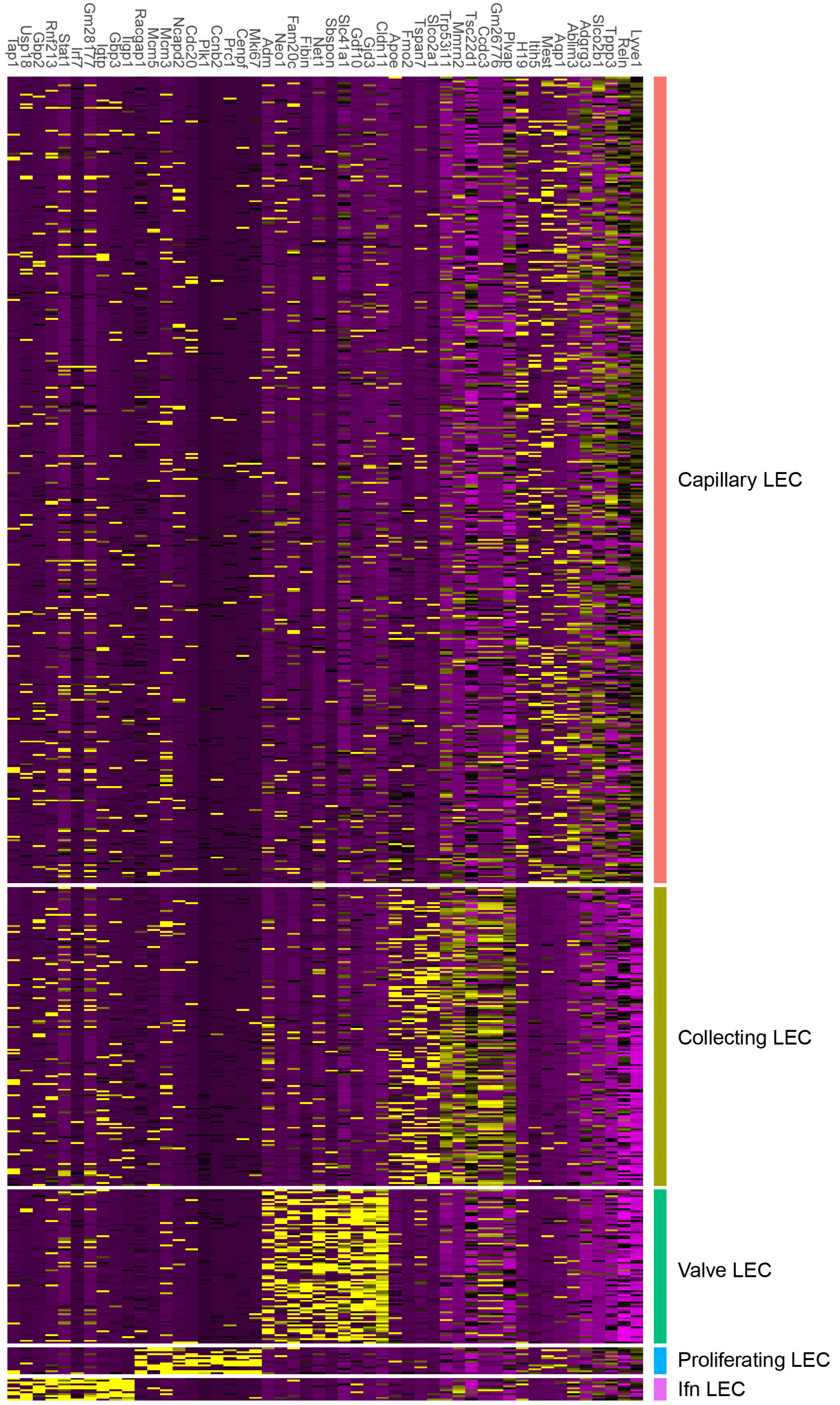

Supplemental Figure 15. Heatmap showing expression of 10 selected differentially expressed genes in each cluster.

Supplemental Figure 16

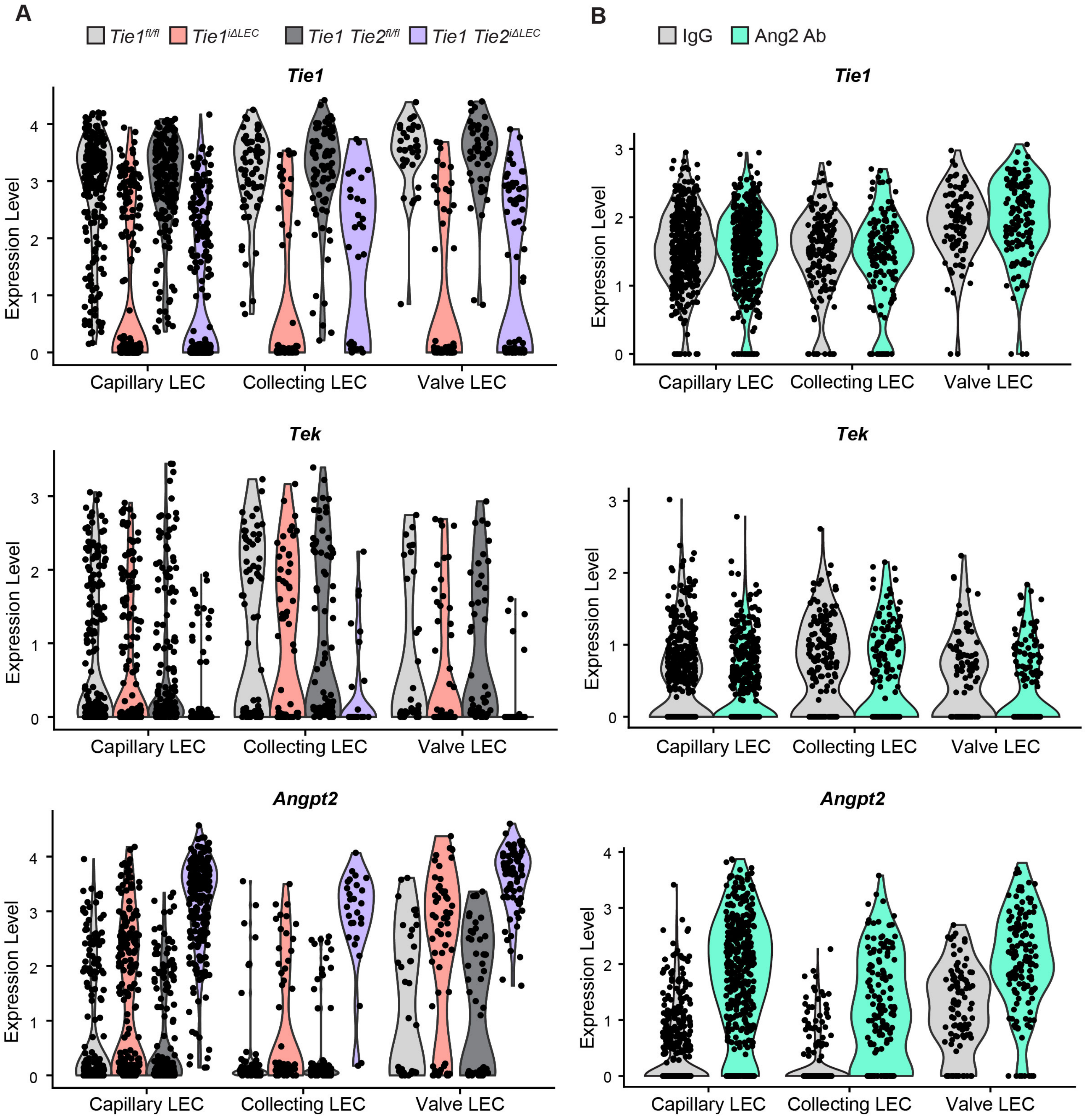

Supplemental Figure 16. Expression of Tie1, Tie2 and Ang2 in Tie1-deleted, Tie1/-2-deleted and Ang2-inhibited LECs. (**A-B**) Violin plots showing the expression of Tie1, Tie2 and Ang2 after Tie1 and Tie1/-2 deletion (**A**) or Ang2-inhibition (**B**). Significant changes are indicated in Supplemental Table 1.

Supplemental Figure 17

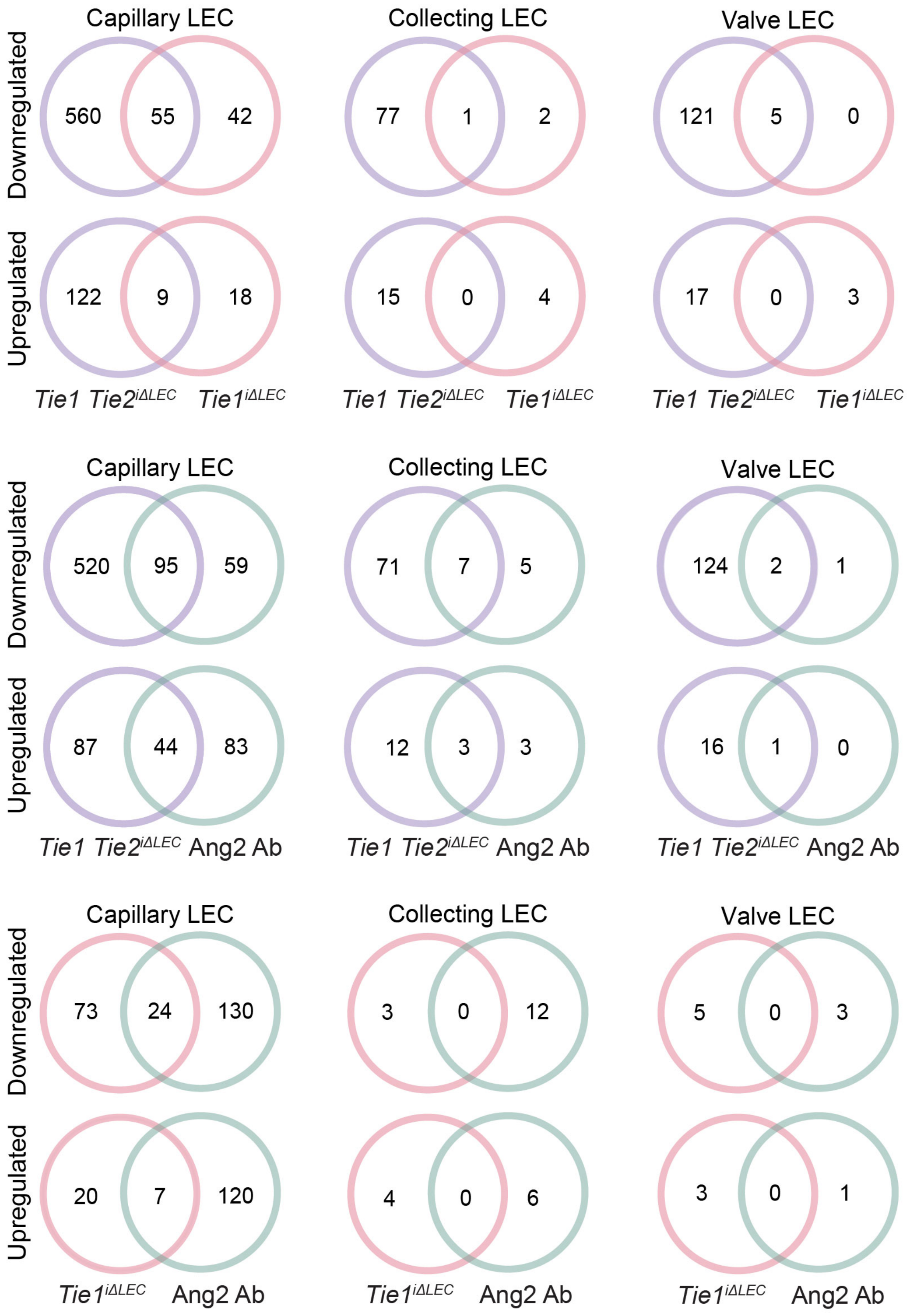

Supplemental Figure 17. Differentially expressed genes in Tie1-deleted, Tie1/-2-deleted and Ang2 Ab-treated LECs. Venn diagram showing the number of up- and downregulated genes between the indicated groups. The threshold was set to 0.25 for avg_log2FC and p_val-j_adj < 0.05.

Supplemental Figure 18

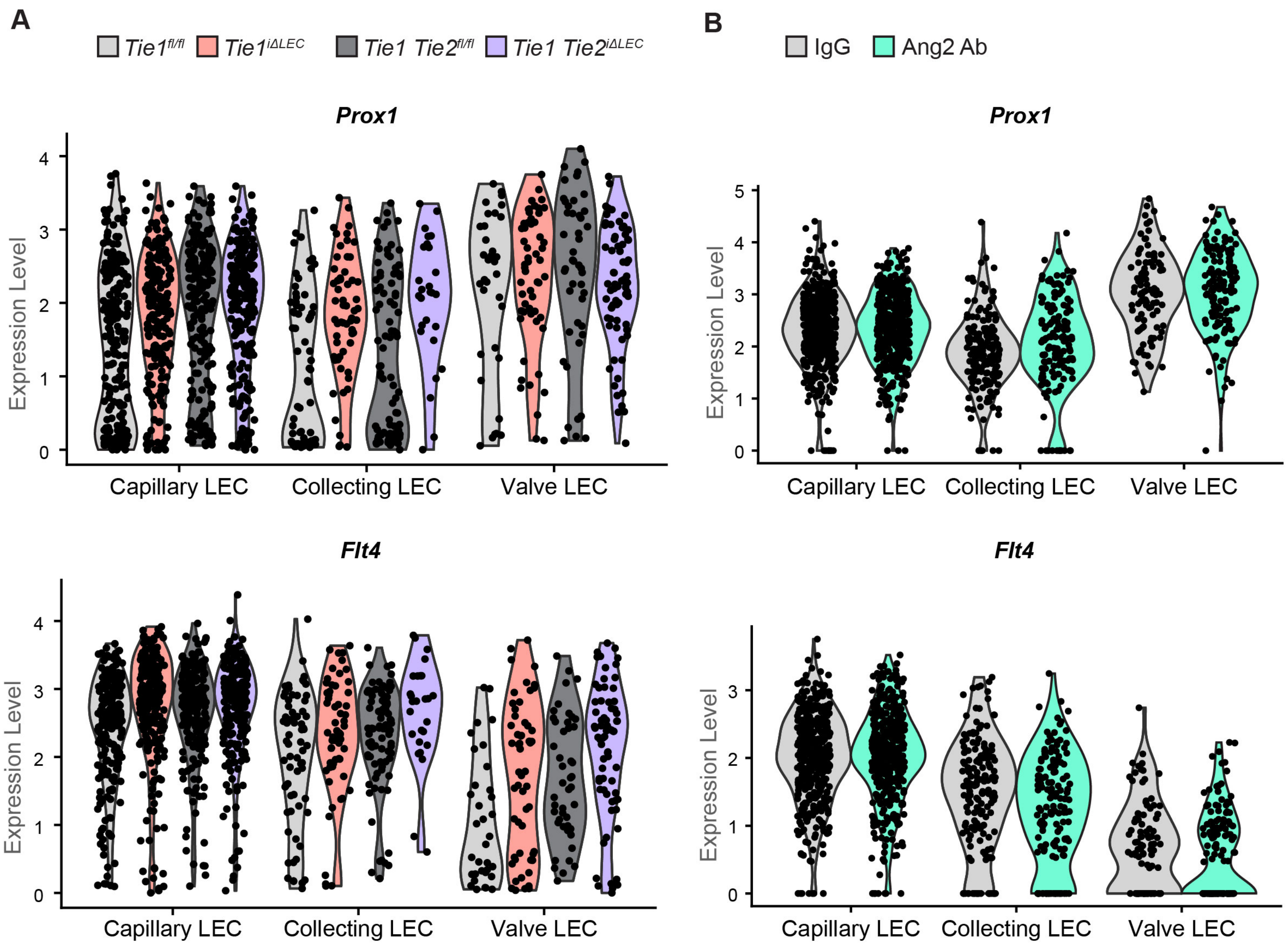

Supplemental Figure 18. Expression of Prox1 and VEGFR3 in Tie1-deleted, Tie1/-2-deleted and Ang2-inhibited LECs. (**A-B**) Violin plots showing the expression of Prox1 and VEGFR3 after Tie1 and Tie1/-2 deletion (**A**) or Ang2-inhibition (**B**).

Supplemental Figure 19

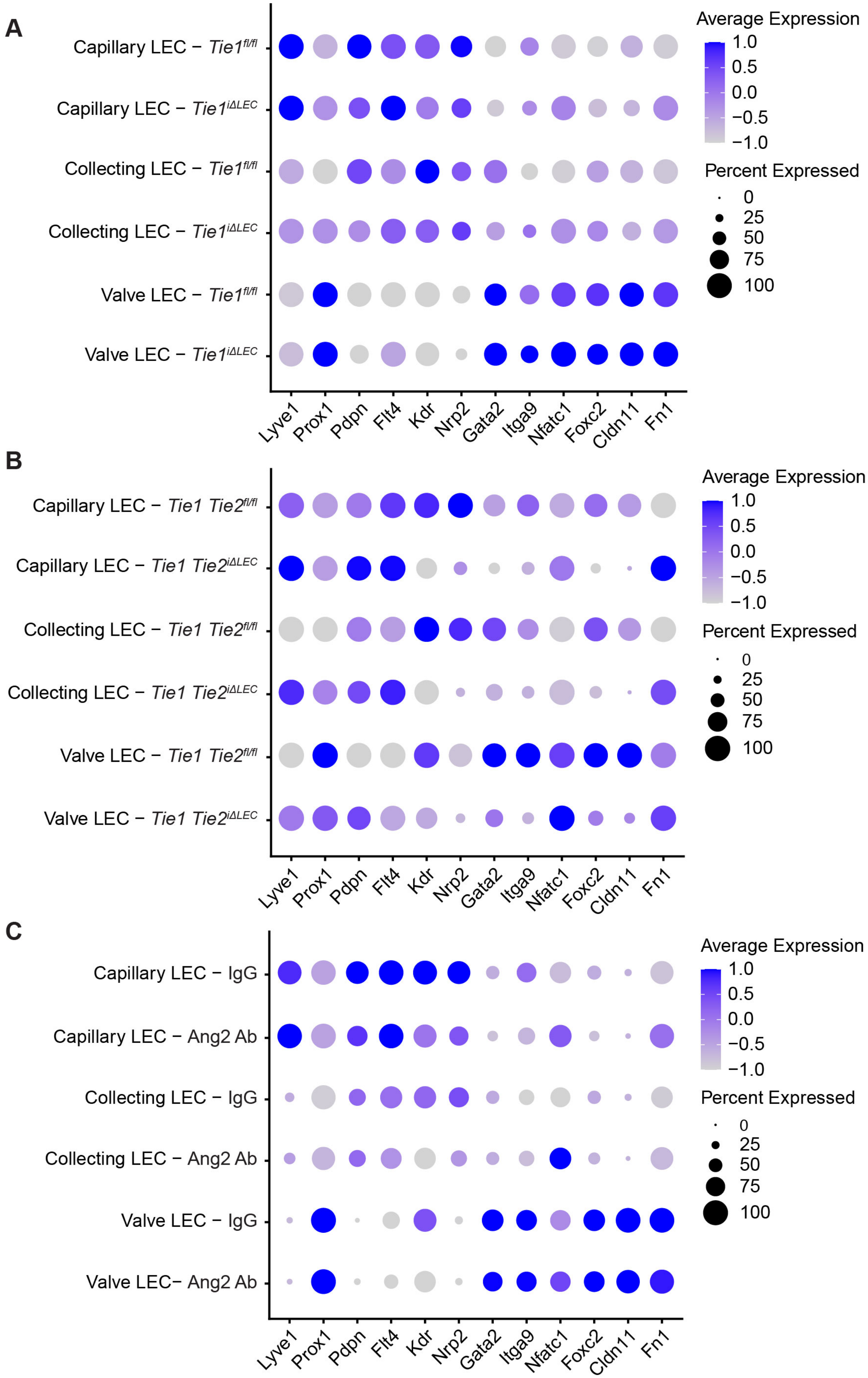

Supplemental Figure 19. Expression of common LEC marker genes in Tie1-deleted, Tie1/-2-deleted and Ang2-inhibited LECs. (**A-C**) Dot plots showing the expression of common LEC marker genes in Tie1-deleted (A), Tie1/-2-deleted (B) or Ang2-inhibited (C) LECs. Significant changes are indicated in Supplemental Table 1.

Supplemental Figure 20

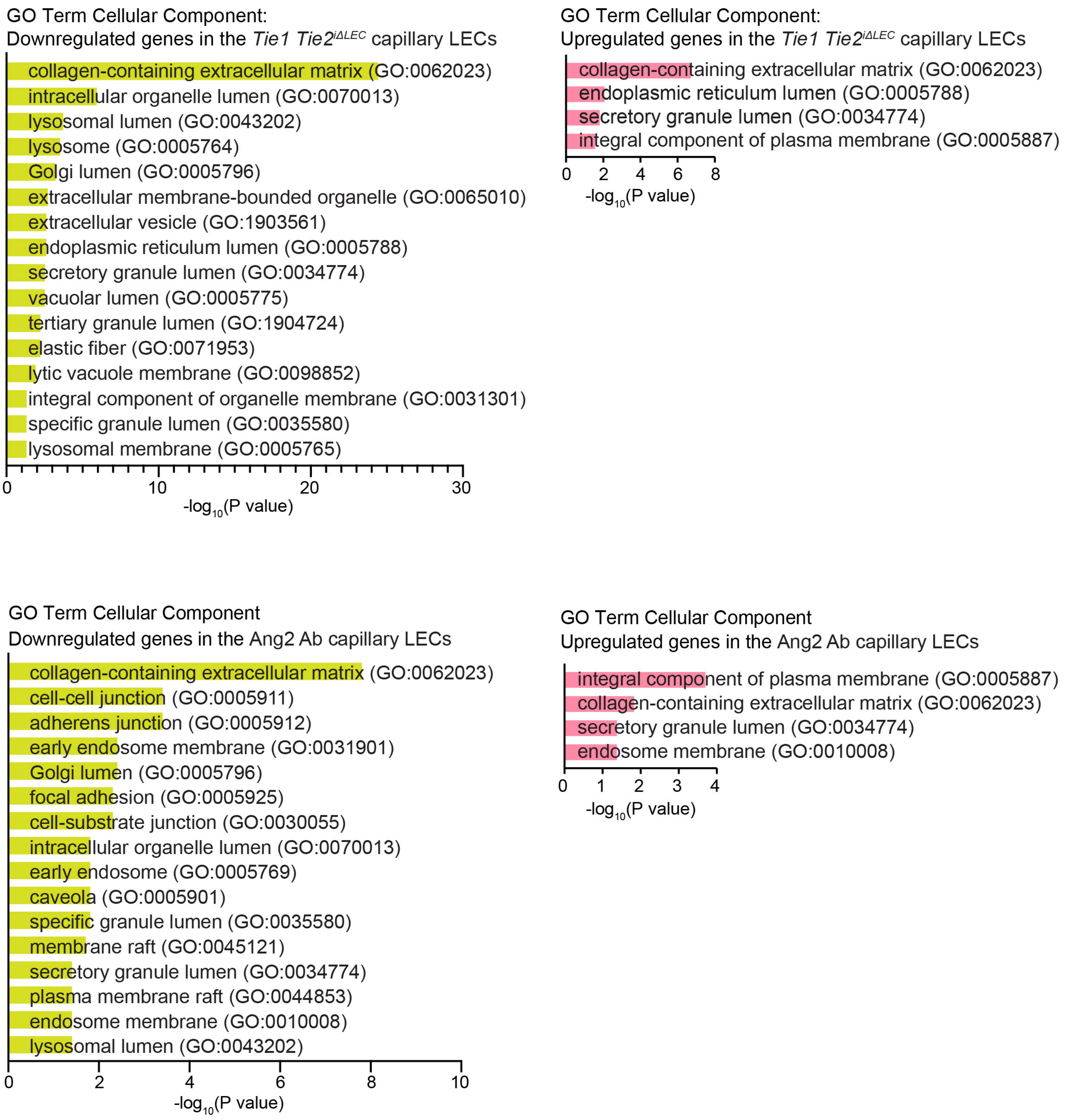

Supplemental Figure 20. Downregulated and upregulated cellular components in Tie1/-2-deleted and Ang2 Ab-treated capillary LECs. GO Cellular Component analysis of significantly (adjusted $P < 0.05$) downregulated genes and selected significantly (adjusted $P < 0.05$) upregulated genes after Tie1/-2 deletion and Ang2 Ab treatment in capillary LECs.

Supplemental Figure 21

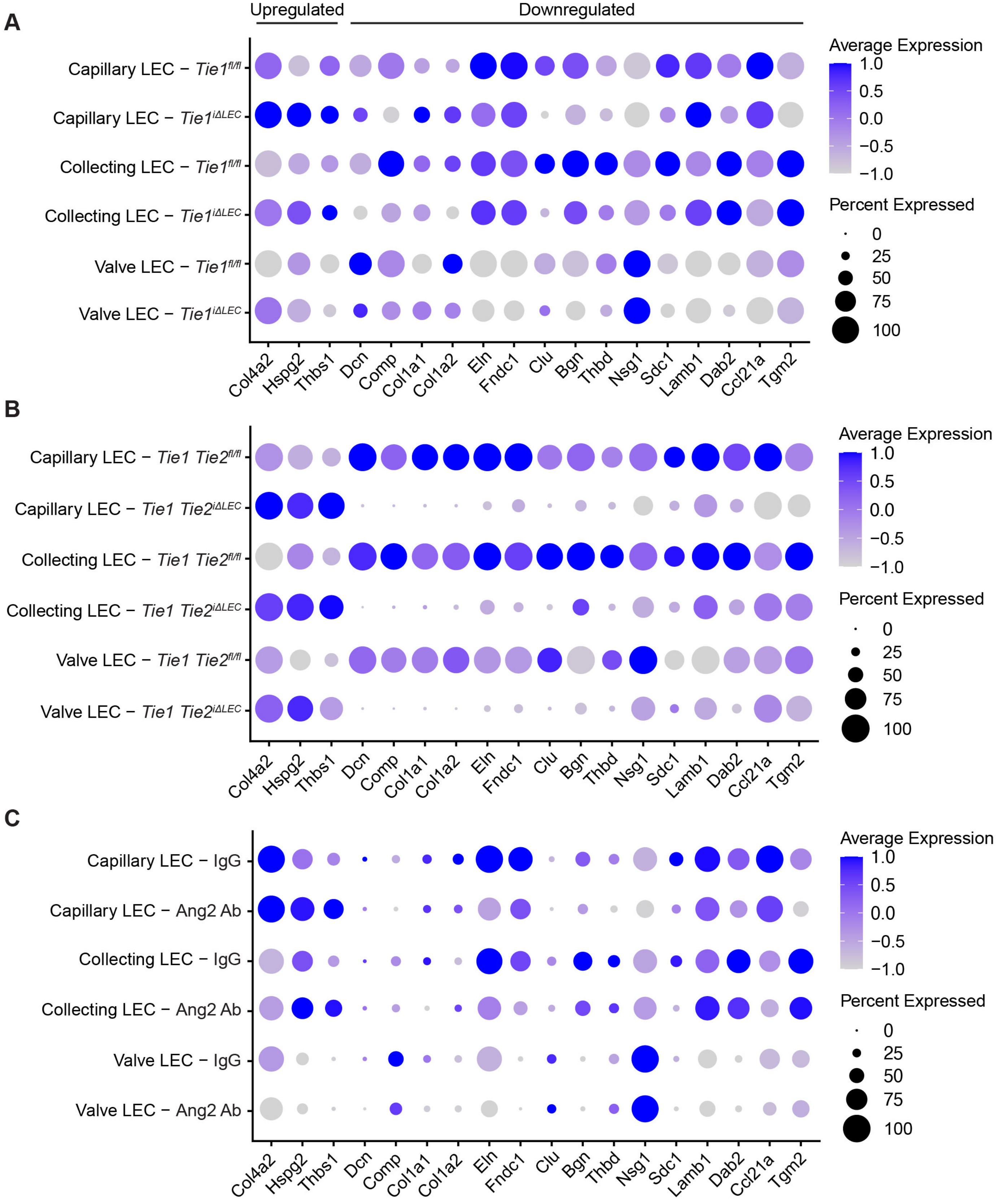

Supplemental Figure 21. Differentially expressed genes in Tie1-deleted, Tie1/-2-deleted and Ang2-inhibited LECs. (**A-C**) Dot plots showing the expression of differentially expressed genes in Tie1-deleted (**A**), Tie1/-2-deleted (**B**) or Ang2-inhibited (**C**) LECs. Significant changes are indicated in Supplemental Table 1.

Supplemental Figure 22

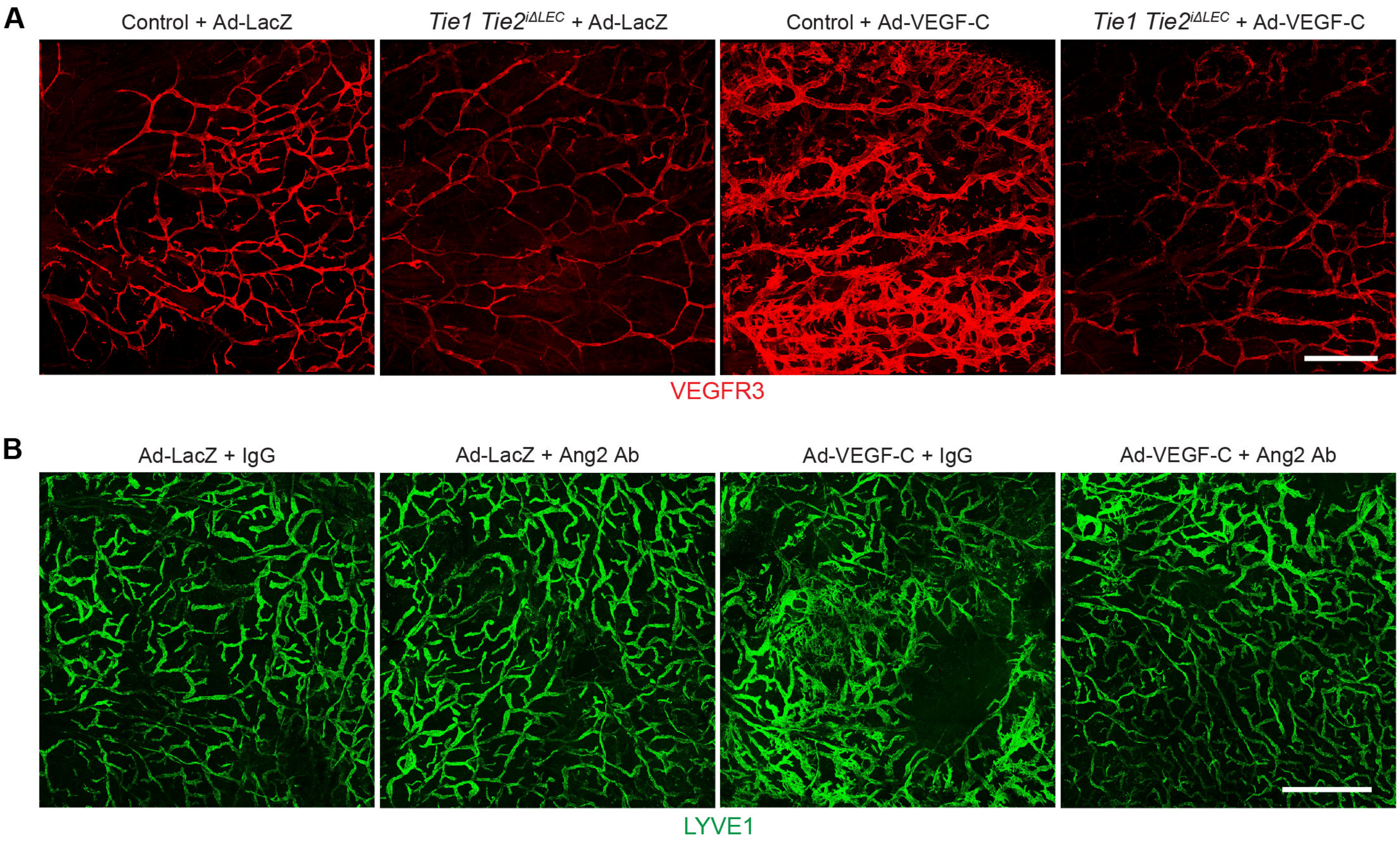

Supplemental Figure 22. Low magnification images of VEGF-C treated ears in adult mice. (**A**) VEGFR3 staining of ear skin in control and Tie1/-2-deleted mice treated with Ad-LacZ or Ad-VEGF-C for 10 days (Ad-LacZ: n=3 ears; Tie1/-2-deleted+Ad-LacZ: n=3 ears; Ad-VEGF-C: n=4 ears; Tie1/-2-deleted+Ad-VEGF-C: n=4 ears). (**B**) LYVE1 staining of ear skin in mice treated for 1.5 weeks with Ad-VEGF-C, Ang2 Ab, or both (n=8 ears per group). Scale bars, 1000 μm.
